# Fertility-Sparing Treatment of Endometrial Cancer and Atypical Endometrial Hyperplasia by Molecular Classification: A Systematic Review and Meta-Analysis Accounting for Outcome-Assessment Timing and Follow-Up Duration

**DOI:** 10.3390/cancers18182972

**Published:** 2026-09-14

**Authors:** Abeer Alatawi, Saleem Almaser, Malak Alanazi, Amirah Alatawi, Seongmin Kim

**Affiliations:** 1 Department of Obstetrics and Gynecology, Korea University Medicine, Seoul 02841, Republic of Korea; abeer.atawi@hotmail.com; 2 Department of Obstetrics and Gynecology, Maternity and Children Hospital (MCH), Tabuk 47717, Saudi Arabia; a.s.e.77@hotmail.com (S.A.); mal0oka95@hotmail.com (M.A.); 3 Department of Internal Medicine, King Fahad Specialist Hospital, Tabuk 47717, Saudi Arabia; amsaalatawi.02@gmail.com

**Keywords:** endometrial cancer, atypical endometrial hyperplasia, fertility-sparing treatment, molecular classification, POLE, mismatch repair deficiency, p53-abnormal, NSMP, complete response, recurrence, meta-analysis

## Abstract

**Background/Objectives**: Molecular classification (POLE-mutated, mismatch-repair–deficient [MMRd], no-specific-molecular-profile [NSMP], p53-abnormal [p53abn]) is used to stratify women receiving fertility-sparing treatment (FST) for early endometrial carcinoma or atypical hyperplasia, but reported subtype differences are inconsistent. We estimated subtype-specific complete response (CR) and post-CR recurrence and examined how outcome-assessment timing (fixed early timepoint versus best response) and follow-up duration affect subtype comparisons. **Methods**: PubMed and Embase (January 2015–August 2025) were searched; overlapping reports were consolidated; proportions were pooled with binomial–normal generalised linear mixed models; late response among early non-responders was analysed in paired cohorts; recurrence was meta-regressed on follow-up; risk of bias (QUIPS) and certainty (GRADE) were assessed. **Results**: Twenty reports yielded 15 cohorts (10 primary). Best CR was 85% (95% CI 70–93) for POLE, 71% (57–83) for MMRd, and 85% (77–91) for NSMP. In six paired cohorts, CR rose from the early timepoint to best response in every subtype (MMRd 44→77%, NSMP 57→90%); 55% of MMRd and 76% of NSMP early non-responders later achieved CR, and within-cohort best CR was lower for MMRd than NSMP (odds ratio [OR] 0.36, 0.17–0.79). Recurrence after CR was 34% (23–46) for MMRd, 30% (17–49) for NSMP, and 19% (4–57; 0–100% across cohorts) for POLE. NSMP recurrence tended to rise with follow-up in an exploratory study-level analysis (OR 1.02/month; *p* = 0.06, dependent on the few long-follow-up cohorts), whereas MMRd recurrence did not (adjusted OR vs. NSMP 1.7, 0.9–3.3). p53abn tumours (n = 28) showed variable CR (17/24) and recurrence in 9/17. Occult p53abn or MMRd tumours affected 17% (12–24%; range 7–47%) of conventionally selected candidates. Certainty was low or very low. **Conclusions**: Subtype comparisons in FST depend on when outcomes are assessed and how long patients are followed. Molecular classification is prognostic, not predictive; its principal value is to identify occult higher-risk tumours and to calibrate counselling and surveillance, not to justify de-escalation.

## Simple Summary

Some younger women with early cancer of the womb lining, or its precursor (atypical hyperplasia), wish to preserve the possibility of having children and can be offered hormone treatment instead of surgery to remove the womb. Doctors increasingly use a tumour’s molecular “type” to estimate how such treatment is likely to go, but published findings have been inconsistent. By combining data from 15 published patient groups, we found that comparisons between molecular types depend heavily on when the response is assessed and on how long patients are followed. In every type, many women who had not responded at six months responded later. Tumours with a mismatch-repair defect responded less often and more slowly and relapsed early; in the largest, “no specific profile” group, relapse tended to accumulate with longer follow-up, although this depended on which long-term studies were included; the rare POLE-mutated tumours responded well, but their long-term durability is uncertain; and p53-abnormal tumours raised concern about durability. About one in six women selected on conventional criteria carried a p53-abnormal or mismatch-repair–deficient tumour. Molecular testing therefore seems most useful for identifying these higher-risk tumours before treatment and for planning follow-up, rather than for relaxing monitoring in apparently favourable cases.

## 1. Introduction

Endometrial carcinoma (EC) is the most common gynecologic malignancy in high-income countries, and its incidence has risen over the past three decades, driven substantially by the global increase in obesity, insulin resistance, and metabolic syndrome [1,2]. Although EC predominantly affects postmenopausal women, a clinically important and growing proportion—approximately 4–7%—occurs in women of reproductive age, for whom the wish to preserve fertility shapes treatment decisions. Abnormal uterine bleeding is the usual presenting symptom of EC and of its precursor, atypical endometrial hyperplasia (AEH); office hysteroscopy with targeted biopsy has become a central tool in the work-up of such bleeding and provides both the histological diagnosis and the tissue on which molecular classification is performed [3].

The standard treatment for EC is total hysterectomy with bilateral salpingo-oophorectomy, which precludes future fertility. For carefully selected patients with early-stage, low-grade disease—and for women with AEH—fertility-sparing treatment (FST) with oral progestins (megestrol acetate or medroxyprogesterone acetate) or a levonorgestrel-releasing intrauterine system, with or without hysteroscopic resection, offers an alternative that has been incorporated into international guidance, including the 2023 ESGO/ESHRE/ESGE recommendations [4]. Within rigorously selected cohorts, complete response (CR) rates of roughly 70–90% are reported, supporting FST as an oncologically acceptable option in appropriate candidates.

Two limitations challenge purely clinicopathologic selection. First, long-term series consistently report relapse in 30–40% of patients who achieve an initial CR, raising concern about the durability of conservative management. Second, marked variability in response persists among patients meeting identical histopathologic and radiologic criteria, implying that morphology and imaging are insufficient surrogates for the tumour biology that governs hormonal sensitivity. The Cancer Genome Atlas (TCGA) taxonomy [5]—POLE-ultramutated, MMRd/microsatellite-unstable, p53abn, and NSMP—operationalised through the ProMisE classifier [6,7] and embedded in the 2020 WHO classification and the ESGO/ESTRO/ESP guidelines [8], has therefore been proposed as a prognostic stratifier for women considering FST.

Individual FST cohorts increasingly report outcomes by molecular subtype [9,10], yet three features of these data make naive pooling unreliable and motivate the present design. First, FST cohorts are single-arm: all patients receive conservative treatment, so molecular subtype can be evaluated only as a prognostic factor (an association with outcome among treated women), not as a predictive marker of differential benefit from FST relative to surgery [11]. The quantity of interest is therefore the subtype-specific proportion achieving CR or recurrence, which is most appropriately synthesised as a proportion rather than as a comparative effect measure requiring a control group. Second, CR is time-dependent: an assessment at a fixed early timepoint (typically 6 months) captures a different quantity from a cumulative best response, so pooling across studies that report different timepoints conflates response kinetics with response magnitude. Third, recurrence after CR accrues with follow-up, so comparing recurrence across cohorts with different follow-up durations conflates surveillance length with biology. Throughout this article we use “outcome-assessment timing” to denote the first issue (fixed early timepoint versus best/cumulative response) and “follow-up duration” to denote the second; together they constitute the “measurement timing” that this review sets out to account for.

We therefore designed a systematic review and meta-analysis that accounts explicitly for both. Data were extracted from primary sources; overlapping reports of the same cohort were consolidated; CR was pooled separately at an early fixed timepoint and at best response, and late response among early non-responders was analysed within paired cohorts; recurrence was modelled against follow-up duration; and subtype contrasts were tested formally. Our aims were to (1) estimate subtype-specific CR and recurrence using proportion-based methods appropriate to single-arm data, (2) determine which subtype differences persist after accounting for outcome-assessment timing and follow-up duration, and (3) define the findings that should inform molecular-integrated counselling and surveillance in fertility-sparing management.

## 2. Materials and Methods

### 2.1. Design, Reporting and Registration

This systematic review and meta-analysis was conducted and reported in accordance with the PRISMA 2020 statement [12]; the completed PRISMA 2020 checklist is provided as Supplementary Materials. The review was not registered in PROSPERO or another prospective registry: registration was overlooked when the review was initiated, and by the time the omission was recognised, screening and data extraction were already underway—PROSPERO does not accept registrations once data extraction has started, so registration after completion of the search was no longer possible. No publicly time-stamped protocol therefore exists; we regard this as a methodological limitation (Section 4.9) because a non-public analysis plan cannot fully exclude data-dependent analytical decisions. To mitigate it, the analysis plan (Supplementary Protocol, version 1.0 dated 2 July 2026, written in PRISMA-P format [13]) is provided, together with a version history and a table that distinguishes pre-specified analyses (proportion-based synthesis; separate pooling of CR at a fixed early timepoint and at best response; consolidation of overlapping cohorts; descriptive treatment of p53abn CR; meta-regression of recurrence on follow-up; MMR-binary, cluster-swap and leave-one-out sensitivity analyses) from analyses added post hoc during peer review (conditional late-response analysis with formal subtype-by-timepoint testing; recurrence models with a subtype-by-follow-up interaction; EC-only and mixed AEH/EC subgroup analyses; progression synthesis; QUIPS risk-of-bias assessment; GRADE re-assessment under a prognostic-factor framework; and public deposition of data and code). The extracted dataset, overlap-resolution table, risk-of-bias assessments, analysis code and figure code are deposited in a public repository (Data Availability Statement).

### 2.2. Eligibility Criteria

**Population:** Women receiving FST for histologically confirmed early-stage EC and/or AEH; cohorts combining EC and AEH were eligible provided outcomes were reported by molecular subtype, and the AEH/EC composition of every cohort was reported. **Intervention:** FST comprising progestins (oral or intrauterine), with or without hysteroscopic resection or adjuncts. **Prognostic factor:** Molecular classification into POLE-mutated, MMRd, p53abn and NSMP by a validated classifier (TCGA, ProMisE, or immunohistochemistry/sequencing surrogates); cohorts reporting only mismatch-repair status (MMR-binary) were eligible for pre-specified sensitivity analyses only. **Outcomes:** CR (primary), recurrence after CR, disease progression during treatment, and reproductive outcomes (secondary). Case reports, series with fewer than 10 participants, reviews, protocols without outcome data, and conference abstracts without full text were excluded, as were non-FST modalities and studies without extractable, subtype-resolved outcomes.

### 2.3. Search and Selection

**Figure 1:**
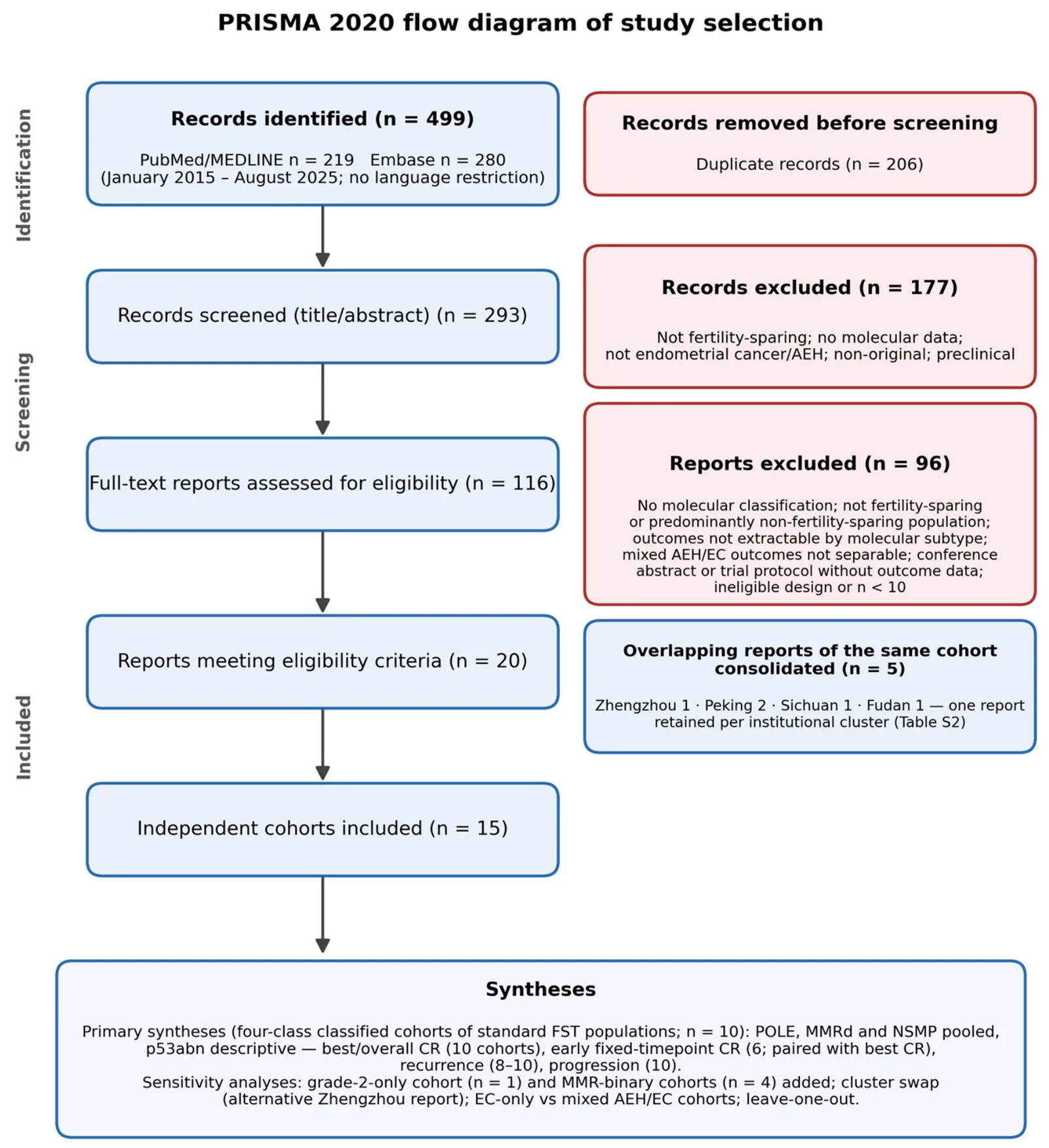
PRISMA 2020 flow diagram of study selection (record counts are those of the original database searches, August 2025). After de-duplication, screening, and full-text assessment, 20 eligible reports were identified; five overlapping reports of the same cohorts were consolidated (one report retained per institutional cluster), yielding 15 independent cohorts. Ten four-class-classified cohorts of standard fertility-sparing populations formed the primary analysis set; the grade-2-only cohort and four MMR-binary cohorts informed sensitivity analyses. Blue boxes denote records or reports carried forward, red boxes denote exclusions, and arrows indicate the flow of records through identification, screening and inclusion; institutional clusters are named by city.

PubMed (MEDLINE) and Embase were searched from 1 January 2015 to 31 August 2025 using controlled vocabulary and free-text terms for endometrial carcinoma/hyperplasia, fertility-sparing or conservative treatment, hormonal therapy, and molecular or genomic classification, combined with Boolean operators; no language restriction was applied at the search stage. The start date was chosen because the ProMisE classifier was first published in 2015 [6] and molecular classification of FST cohorts began thereafter. Reference lists of eligible studies and relevant reviews were searched backwards; forward citation tracking, Web of Science, Scopus, CENTRAL and trial registries were not searched, which we acknowledge as a limitation. Because the database strings as executed in August 2025 were not archived, the strategy was reconstructed from the concept blocks recorded in the protocol and re-executed in both databases on 19 August 2026 with the original date limits; the reconstruction retrieved every eligible report indexed within the search window (113 PubMed and 419 Embase records), and screening of its yield identified no additional eligible cohort (Supplementary Table S1a gives the strings, yields and sensitivity checks; the record counts in Figure 1 are those of the original searches). Supplementary searches of Europe PMC and Crossref during revision likewise identified no additional cohort meeting the eligibility and outcome criteria (a three-class report without POLE assessment that reported CR only at 3 months [14], and an MMR-binary consecutive series of women up to 55 years of age not restricted to fertility preservation [15], were not eligible). One eligible report published after the search date (Hirano et al., online 9 November 2025 [16]) was identified by hand-searching while the manuscript was in preparation and was included because it met all criteria; the search was not otherwise updated beyond 31 August 2025 (Section 4.9). Titles, abstracts, and full texts were screened in duplicate, with disagreements resolved by consensus.

### 2.4. Data Extraction

Data were extracted directly from primary sources by two reviewers and, during revision, re-extracted in full from the full text of every primary report (the eight reports published in subscription journals were obtained as publisher PDFs); the re-extraction identified and corrected several errors of the original extraction, which are listed in the response to reviewers and in the deposited extraction notes. For each cohort we recorded design, country, institution, enrolment period, sample size and AEH/EC composition, histological grade, FIGO stage and the method used to exclude myometrial invasion, whether standard FST eligibility criteria were applied, treatment modality (progestin type and route, hysteroscopic resection, and adjuncts such as gonadotropin-releasing hormone agonist, letrozole, metformin, or immune checkpoint inhibition), the definition of CR (including the number of negative samplings required) and the assessment schedule, the molecular classifier (method, algorithm, specimen source and its timing relative to treatment, and handling of multiple-classifier tumours), and median follow-up with its definition. Subtype-specific numerators and denominators were captured for CR at fixed early timepoints and at best/overall response, for recurrence among CR-achievers, for disease progression during treatment, and for reproductive outcomes. Recurrence was defined as histologically confirmed disease after a documented CR; persistent disease as failure to achieve CR without progression; and progression during treatment as defined by each study (typically a higher-grade lesion, myometrial invasion or extrauterine disease, or progression of AEH to carcinoma), with the study-specific definitions recorded (Supplementary Table S4). Whether denominators comprised all treated patients or evaluable patients only was recorded (Supplementary Table S1a,b). Numerators and denominators were independently recalculated for consistency; values derived arithmetically from printed percentages are flagged in the deposited dataset.

### 2.5. Resolution of Overlapping Cohorts

Four overlap clusters were identified from shared institution, senior author, and enrolment window—one each centred on Zhengzhou, Peking, Sichuan, and Fudan. To prevent double-counting, a single cohort was retained per cluster, prioritising the report with the most complete subtype distribution, dual CR timepoints, and multivariable or fertility data; the retained and consolidated reports and the reasons are listed in Supplementary Table S2. The alternative Zhengzhou report [17] was used in a pre-specified cluster-swap sensitivity analysis. Cohorts reporting only mismatch-repair status (MMR-binary) and the single grade-2-only cohort were reserved for sensitivity analyses, and a predominantly non-FST cohort was excluded.

### 2.6. Risk-of-Bias Assessment

Because molecular subtype is evaluated here as a prognostic factor within a treated population rather than as an intervention, risk of bias was assessed with the Quality In Prognosis Studies (QUIPS) tool [18] across its six domains (study participation, study attrition, prognostic-factor measurement, outcome measurement, confounding, and statistical analysis/reporting), with an overall judgement per study; ratings were made by two reviewers and disagreements resolved by consensus (Supplementary Table S3). ROBINS-I, used in the originally submitted version, was replaced because it addresses intervention effects.

### 2.7. Statistical Analysis

**Choice of effect measure.** FST cohorts are single-arm; the clinically meaningful quantity is the subtype-specific proportion achieving CR or recurrence. Proportions were therefore synthesised directly. Subtype contrasts were expressed as odds ratios (ORs) from mixed models that include subtype as a fixed effect and a cohort random intercept, so that comparisons are made within cohorts wherever possible.

**Pooling model.** Let y_i denote events among n_i patients in cohort i. Proportions were pooled with the binomial–normal random-intercept generalised linear mixed model (logit-GLMM) [19,20]: y_i | u_i ~ Binomial(n_i, p_i), logit(p_i) = μ + u_i, u_i ~ N(0, τ^2^), which uses the exact binomial likelihood and accommodates zero-event and all-event cells without continuity corrections. This one-stage approach was chosen over conventional two-stage random-effects pooling of transformed proportions (inverse-variance weighting with the DerSimonian–Laird estimator of τ^2^) because two-stage methods replace the within-cohort binomial likelihood with a normal approximation and require continuity corrections for zero-event and all-event cohorts—frequent in these data (for example, six of the ten POLE best-CR strata)—which biases pooled estimates of extreme proportions toward 50% and misestimates their uncertainty [19,20]. A post hoc side-by-side comparison with DerSimonian–Laird pooling is provided in Supplementary Table S6e: the two approaches agree to within about two percentage points wherever boundary cells are rare, and diverge exactly where the continuity corrections operate (POLE best CR 84.6% versus 74.9%; POLE recurrence 18.7% versus 34.8%). The marginal likelihood was evaluated by adaptive Gauss–Hermite quadrature with 40 nodes (integrand re-centred at each cohort’s conditional mode, located by damped Newton–Raphson iterations to a tolerance of 1 × 10^−10^, and re-scaled by its curvature [21]) and maximised over (μ, log τ) by the Nelder–Mead simplex (parameter tolerance 1 × 10^−8^, function tolerance 1 × 10^−10^, maximum 5000 iterations) from three starting values, followed by BFGS polishing (gradient tolerance 1 × 10^−8^); convergence was declared when the gradient norm was <1 × 10^−5^. τ was bounded below at 1 × 10^−4^; estimates below 1 × 10^−3^ are reported as τ = 0 (boundary solution) with standard errors from the fixed-effect fit. Standard errors were obtained from the numerically differentiated observed information matrix; 95% confidence intervals were computed on the logit scale and back-transformed, and 95% prediction intervals for a new cohort were computed as μ ± 1.96·√(SE^2^ + τ^2^) [22]. Heterogeneity is summarised by τ (between-cohort standard deviation on the logit scale) and the prediction interval, which we consider more informative than I^2^ for single-proportion meta-analysis. The implementation (Python 3.11.15; NumPy 2.4.4; SciPy 1.17.1; statsmodels 0.14.6; glmm_agq 1.2.0) was validated against metafor 4.4.0 (rma.glmm, measure “PLO”, maximum likelihood, adaptive quadrature with 25 nodes) and lme4 1.1-35 in R 4.3.3 [23,24]: for all 18 models reported here, estimates, standard errors and τ agreed to within 3 × 10^−5^ on the logit scale (Supplementary Table S6a–e).

**Outcome-assessment timing (fixed early timepoint versus best response).** CR was pooled separately at a fixed early timepoint (6 months in four cohorts; 32 weeks in one cohort, which reported 16- and 32-week assessments; and the second 3-monthly evaluation, labelled 8 months, in one cohort that reported 4-, 8- and 12-month assessments) and at best/overall response. Because best response is a cumulative endpoint that necessarily includes early responders, we did not treat the two timepoints as independent samples. Instead, in cohorts reporting both timepoints for the same patients (paired cohorts), best response was decomposed into two disjoint, conditionally independent components: CR at the early timepoint (a of N) and late CR among patients without CR at the early timepoint (b of N − a). The conditional late-response proportion b/(N − a) was pooled by subtype, and subtype differences in early CR, in conditional late CR and in best CR were tested with logit-GLMMs containing subtype fixed effects (NSMP as reference) and a cohort random intercept shared across subtypes (Wald tests and likelihood-ratio tests). This replaces the “within-patient” wording used in the original submission, which was inappropriate for aggregate data. For completeness we also report the aggregate two-timepoint model with cohort × subtype strata, a timepoint term and a subtype-by-timepoint interaction, noting that it treats the two timepoints as independent samples. Study-level medians of time to CR by subtype were tabulated where reported. Individual-patient time-to-CR analyses were not possible with aggregate data.

**Follow-up duration.** Recurrence among CR-achievers was pooled by subtype and meta-regressed on each cohort’s reported median follow-up (logit-linear, random intercept), with likelihood-ratio and Wald tests, leave-one-out analyses, and model predictions restricted to the observed follow-up range. Because follow-up is a study-level covariate, this association is ecological and exploratory [25]. A joint model of MMRd and NSMP recurrence with subtype, centred follow-up and their interaction (cohort random intercept) tested whether the follow-up dependence differed between subtypes and provided a follow-up-adjusted MMRd-versus-NSMP contrast.

**Other syntheses.** p53abn CR was summarised descriptively rather than pooled, owing to small and unstable denominators. Disease progression during treatment was pooled descriptively by subtype in cohorts reporting it, with each study’s definition tabulated. Occult higher-risk disease (p53abn and MMRd among cohorts that classified all conventionally selected candidates before treatment) was pooled as a proportion, with the number needed to test computed as the inverse of the combined prevalence; cohorts that enrolled only p53-wild-type patients, tested only responders, or classified specimens obtained after treatment were excluded from these estimates. Reproductive outcomes were summarised narratively.

**Sensitivity and subgroup analyses.** Primary syntheses comprised the four-class-classified cohorts of standard FST populations. Pre-specified sensitivity analyses comprised (i) an MMR-binary expansion adding cohorts reporting only MMR status; (ii) a cluster swap exchanging the retained Zhengzhou cohort for the alternative report; and (iii) leave-one-out across all pools and the meta-regression. Post hoc analyses added during revision comprised (iv) inclusion of the grade-2-only cohort; (v) EC-only versus mixed AEH/EC cohorts (subtype-resolved AEH/EC outcomes were not reported by the mixed cohorts, so patient-level EC-only analyses were not possible); (vi) exclusion of the cohort in which MMRd patients received immune checkpoint inhibitors; and (vii) exclusion of the two cohorts whose early assessment was not at 6 months (32 weeks; 8 months). Certainty of evidence was rated per finding with the GRADE framework for prognostic factors [26,27], in which observational evidence starts at high certainty and is rated down for risk of bias, inconsistency, indirectness, imprecision and publication bias, using the standard four categories (high, moderate, low, very low). Two-sided *p* < 0.05 was considered statistically significant; no adjustment for multiplicity was made, and *p* values are interpreted descriptively.

## 3. Results

### 3.1. Study Selection and Resolution of Overlap

After screening and full-text assessment, 20 eligible reports were identified; consolidating five overlapping reports of the same cohorts (Zhengzhou 1, Peking 2, Sichuan 1, Fudan 1; Supplementary Table S2) yielded 15 independent cohorts (Figure 1; Table 1). Ten cohorts with four-class classification of standard FST populations formed the primary analysis set (647 patients evaluable for response; one of these cohorts classified 116 patients, of whom 80 were evaluable); the grade-2-only cohort (n = 23) and four MMR-binary cohorts (n = 330) informed sensitivity analyses. All ten primary cohorts contributed to best-CR estimates, six to early-timepoint estimates and to the paired analysis, and eight to ten to recurrence estimates, depending on subtype. Cohort characteristics—including AEH/EC composition, grade, stage and myometrial-invasion assessment, treatment (including hysteroscopic resection), CR definition and timepoints, molecular classifier, specimen source and timing, follow-up and its definition—are given in Table 1 and, in full, in Supplementary Table S1a,b.

### 3.2. Risk of Bias in the Included Cohorts

On QUIPS, eight cohorts were judged at moderate and seven at high overall risk of bias (Supplementary Table S3); among the ten primary cohorts, six were moderate and four high. Study participation was rated high risk in six cohorts in which molecular data were available only for a selected subset—patients with archival tumour tissue (33 of 80 treated), patients with next-generation sequencing performed largely on hysterectomy specimens, patients with sufficient tumour DNA and consent, classifiable cases within a trial sub-study (15 of 30 enrolled), patients selected on the availability of molecular data in a multicentre grade-2 series, and complete responders with at least two years of follow-up. Prognostic-factor measurement was rated moderate in most cohorts because of surrogate classifiers (Sanger sequencing of limited POLE exons, mutation-only or immunohistochemistry-only definitions of MMRd and p53abn, unreported specimen timing) and low in cohorts using comprehensive sequencing on pre-treatment tissue. Confounding was rated high in the nine cohorts without adjustment for treatment or clinicopathological differences between subtypes, including cohorts in which treatment differed systematically by subtype (immune checkpoint inhibition in MMRd patients; non-progestin regimens). Outcome measurement was generally at low risk because histological response was assessed at protocol-defined intervals with explicit definitions; it was rated moderate in the two cohorts in which relapse after CR was either not reported by subtype or reported as crude proportions at fixed years without censoring. These features are reflected in the certainty ratings (Section 3.9).

### 3.3. Best/Overall Complete Response by Subtype

**Figure 2:**
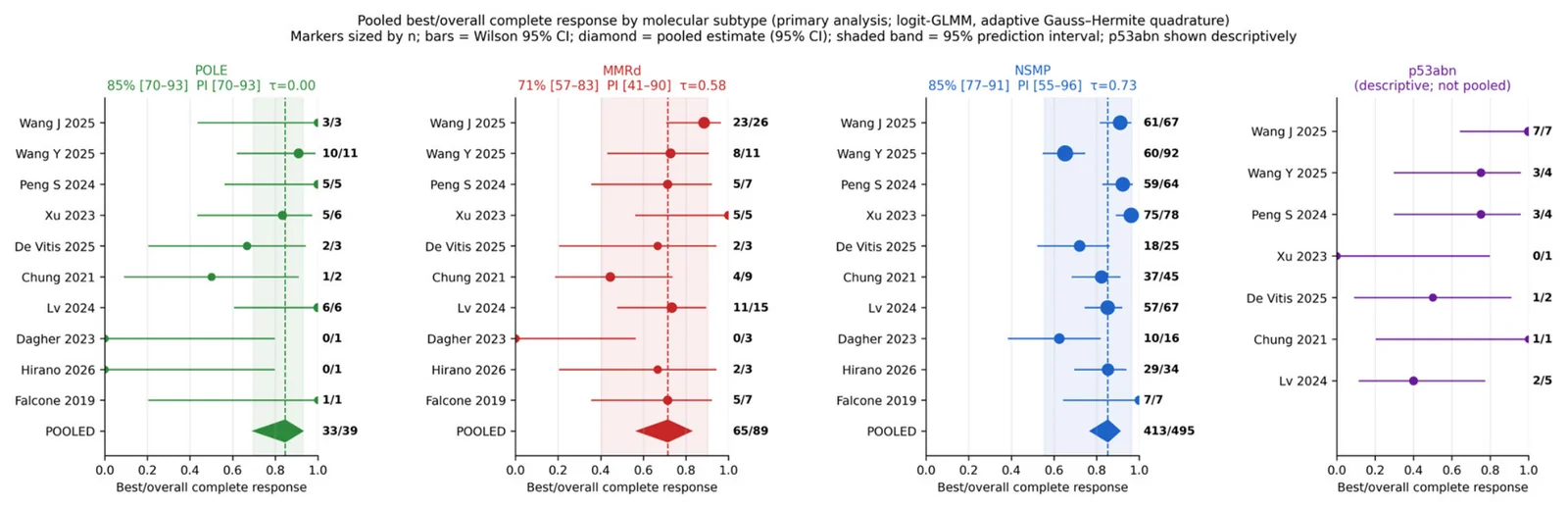
Forest plots of pooled best/overall complete response (CR) by molecular subtype in the primary analysis set (logit-GLMM with adaptive Gauss–Hermite quadrature). Study markers are sized by sample size, with Wilson 95% confidence intervals; event/total counts are printed for every cohort; pooled estimates are shown as diamonds with 95% confidence intervals, shaded 95% prediction intervals (PI) and between-study SD (τ). p53abn is shown descriptively (no pooled estimate). Cohort labels refer to Falcone 2019 [28], Chung 2021 [29], Dagher 2023 [31], Xu 2023 [32], Lv 2024 [33], Peng S. 2024 [35], De Vitis 2025 [37], Wang J. 2025 [40], Wang Y. 2025 [41] and Hirano 2026 [16]; colours denote subtype (green, POLE; red, MMRd; blue, NSMP; purple, p53abn).

At best/overall response, pooled CR in the primary set was 84.6% (95% CI 69.7–92.9; τ = 0; 33/39) for POLE, 71.5% (95% CI 57.1–82.5; prediction interval 41–90; τ = 0.58; 65/89) for MMRd, and 85.3% (95% CI 77.0–91.0; prediction interval 55–96; τ = 0.73; 413/495) for NSMP (Figure 2). Estimates were stable to leave-one-out (POLE 82–87%, MMRd 67–75%, NSMP 83–87%), to inclusion of the grade-2-only cohort (85%, 70%, 84%), and to exclusion of the cohort in which MMRd patients received immune checkpoint inhibitors (MMRd 70.9%, 95% CI 53.7–83.7). Within the six paired cohorts, the MMRd-versus-NSMP odds of best CR were 0.36 (95% CI 0.17–0.79; *p* = 0.011; likelihood-ratio test across subtypes *p* = 0.044). EC-only cohorts and mixed AEH/EC cohorts gave similar estimates for POLE (82% vs. 82%) and MMRd (68% vs. 71%), and a non-significantly higher NSMP CR in EC-only cohorts (90% vs. 80%; OR 2.10, 95% CI 0.77–5.78; *p* = 0.16). p53abn CR is shown descriptively in Figure 2 and Section 3.7.

### 3.4. Outcome-Assessment Timing: Early Fixed Timepoint Versus Best Response

**Early fixed-timepoint CR.** In the six cohorts reporting a fixed early assessment, pooled CR was 43.7% (95% CI 23.3–66.4; τ = 0.78; 28/57) for MMRd and 57.0% (95% CI 45.1–68.2; τ = 0.49; 166/286) for NSMP; POLE estimates were too sparse and heterogeneous to summarise (13/20 across six cohorts; τ = 2.4). Excluding the two cohorts assessed at 32 weeks or 8 months rather than 6 months gave 35.9% (MMRd) and 53.8% (NSMP).

**Figure 3:**
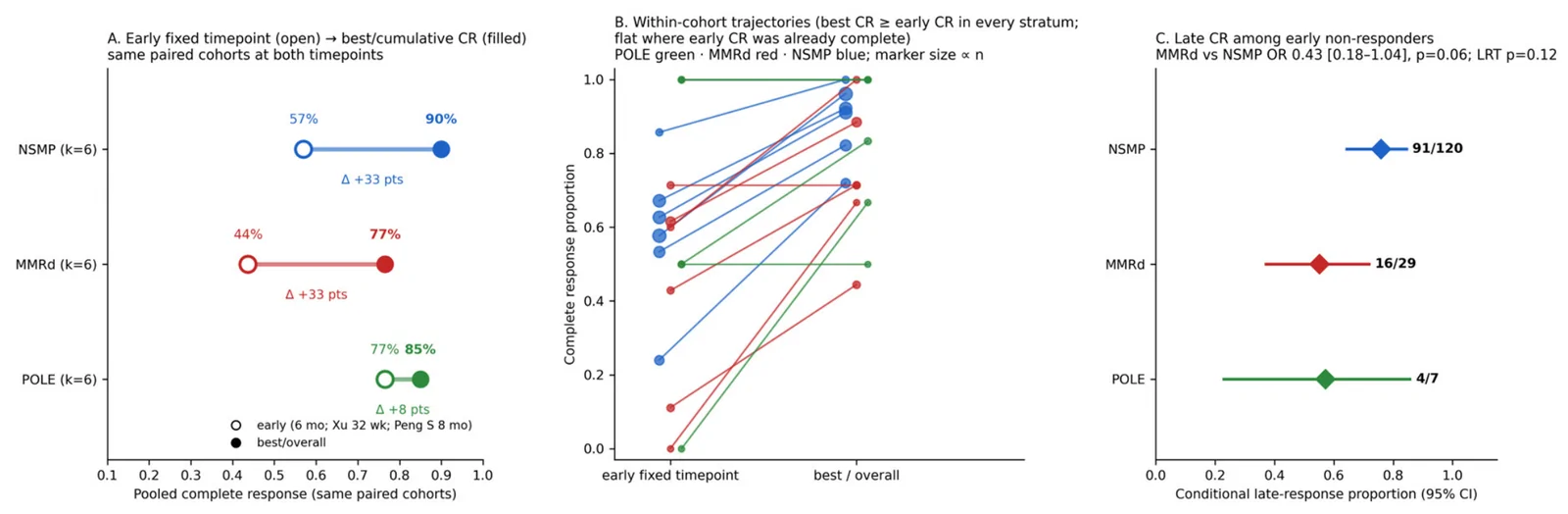
Outcome-assessment timing in the six paired cohorts (Wang J. 2025 [40], De Vitis 2025 [37], Chung 2021 [29], Falcone 2019 [28], Xu 2023 [32], Peng S. 2024 [35]) that reported complete response (CR) both at a fixed early timepoint (6 months; 32 weeks in Xu 2023 [32]; 8 months in Peng S. 2024 [35]) and at best/overall response for the same patients. (**A**) Pooled CR at the early timepoint (open) and at best response (filled), by subtype; the same cohorts contribute to both estimates. (**B**) Within-cohort trajectories; best CR is at least as high as early CR in every stratum. (**C**) Conditional late CR among patients without CR at the early timepoint (event/total shown), with the mixed-model test of subtype differences. Colours denote subtype (green, POLE; red, MMRd; blue, NSMP). In (**A**), open and filled circles denote the early-timepoint and best-response estimates, respectively; in (**B**), marker size is proportional to the number of patients; in (**C**), diamonds denote the pooled conditional late-CR proportions and horizontal lines their 95% confidence intervals.

**Paired cohorts.** In the six cohorts reporting both timepoints for the same patients (Tables S5a and S5b), CR rose from the early timepoint to best response in every subtype, and best CR was at least as high as early CR in every cohort–subtype stratum (Figure 3): pooled MMRd CR rose from 43.7% to 76.5% (28/57 → 44/57), NSMP from 57.0% to 90.0% (166/286 → 257/286) and POLE from 76.5% to 85.0% (13/20 → 17/20). The absolute increase was similar for MMRd and NSMP (+33 percentage points each), but it arose differently: MMRd started from a lower early CR, leaving a larger pool of early non-responders, whereas the conditional probability that an early non-responder subsequently achieved CR was lower for MMRd—55.2% (95% CI 37.2–71.9; 16/29) versus 75.8% (95% CI 64.4–84.5; 91/120) for NSMP and 4/7 for POLE (MMRd vs. NSMP OR 0.43, 95% CI 0.18–1.04; *p* = 0.06; likelihood-ratio test across subtypes *p* = 0.12). The aggregate two-timepoint model, which treats the two timepoints as independent samples (Section 2.7), showed no subtype-by-timepoint interaction (ratio of timepoint ORs, MMRd vs. NSMP, 0.64, 95% CI 0.23–1.81; Wald *p* = 0.40; likelihood-ratio test *p* = 0.70). MMRd early CR was lower than NSMP (OR 0.58, 95% CI 0.31–1.07; *p* = 0.08) and remained lower at best response (OR 0.36, 95% CI 0.17–0.79; *p* = 0.011). Consistent with slower response, two of the three cohorts reporting median time to CR by subtype found longer medians for MMRd than for NSMP (11 vs. 6.5 months; 7.9 vs. 3.7 months) [35,41], whereas the third found no difference (4.0 vs. 4.0 months) [40], and one cohort reported significantly lower MMRd CR both at 6 months (11% vs. 53%) and at best overall response (44% vs. 82%) [29]. Taken together, a fixed 6-month assessment understates eventual CR in every subtype; MMRd is associated with less frequent and slower response, and its early non-responders were, if anything, less likely than NSMP non-responders to convert—the data therefore do not support a distinct MMRd-specific delayed-response biology.

### 3.5. Follow-Up Duration: Recurrence After Complete Response

**Figure 4:**
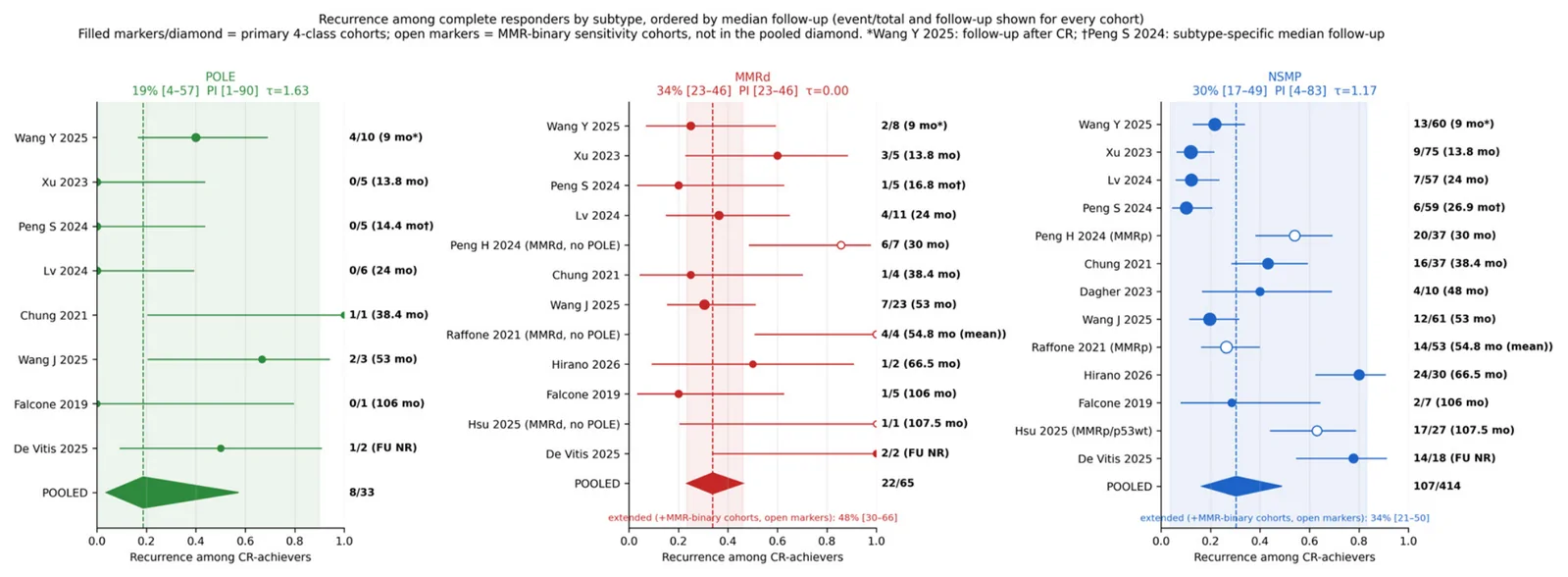
Recurrence among CR-achievers by subtype, ordered by median follow-up, with event/total counts and follow-up printed for every cohort. Filled markers and the pooled diamond are the primary four-class cohorts; open markers are the MMR-binary sensitivity cohorts, which are not included in the diamond (their addition is summarised beneath each panel). Cohort labels refer to Falcone 2019 [28], Chung 2021 [29], Raffone 2021 [30], Dagher 2023 [31], Xu 2023 [32], Lv 2024 [33], Peng H. 2024 [34], Peng S. 2024 [35], De Vitis 2025 [37], Hsu 2025 [39], Wang J. 2025 [40], Wang Y. 2025 [41] and Hirano 2026 [16]; diamonds denote pooled estimates with 95% confidence intervals, shaded bands the 95% prediction intervals, and colours the subtype (green, POLE; red, MMRd; blue, NSMP). * Wang Y. 2025 [41]: follow-up after CR; † Peng S. 2024 [35]: subtype-specific median follow-up.

**Pooled recurrence.** Among CR-achievers in the primary set (Figure 4), pooled recurrence was 33.8% (95% CI 23.4–46.1; τ = 0; 22/65; leave-one-out 32–36%) for MMRd, 30.3% (95% CI 16.6–48.8; prediction interval 4–83; τ = 1.17; 107/414) for NSMP and 18.7% (95% CI 3.8–57.3; prediction interval 1–90; τ = 1.63; 8/33) for POLE. The POLE estimate should not be read as a point estimate: recurrence was 0/5, 0/5, 0/6 and 0/1 in the four cohorts followed for 14, 14, 24 and 106 months, but 1/1 (at 66 months) and 2/3 in the cohorts followed 38–53 months, 1/2 in a cohort without reported follow-up, and 4/10 within about two years of CR in one short-follow-up cohort, so the summary is dominated by a handful of events in tiny denominators. In a subtype model with a cohort random intercept, the odds of recurrence were 1.80-fold higher for MMRd than for NSMP (95% CI 0.97–3.35; *p* = 0.065) and similar for POLE and NSMP (OR 1.19; *p* = 0.70). Adding the three MMR-binary cohorts that report recurrence by MMR status raised the MMRd estimate to 47.6% (95% CI 29.6–66.2; τ = 0.85; 33/77), driven by two small MMRd groups from cohorts consisting largely or entirely of atypical hyperplasia, in which 4/4 and 6/7 responders relapsed, and changed the NSMP/MMR-proficient estimate to 34.1% (95% CI 21.3–49.6; 158/531).

**Figure 5:**
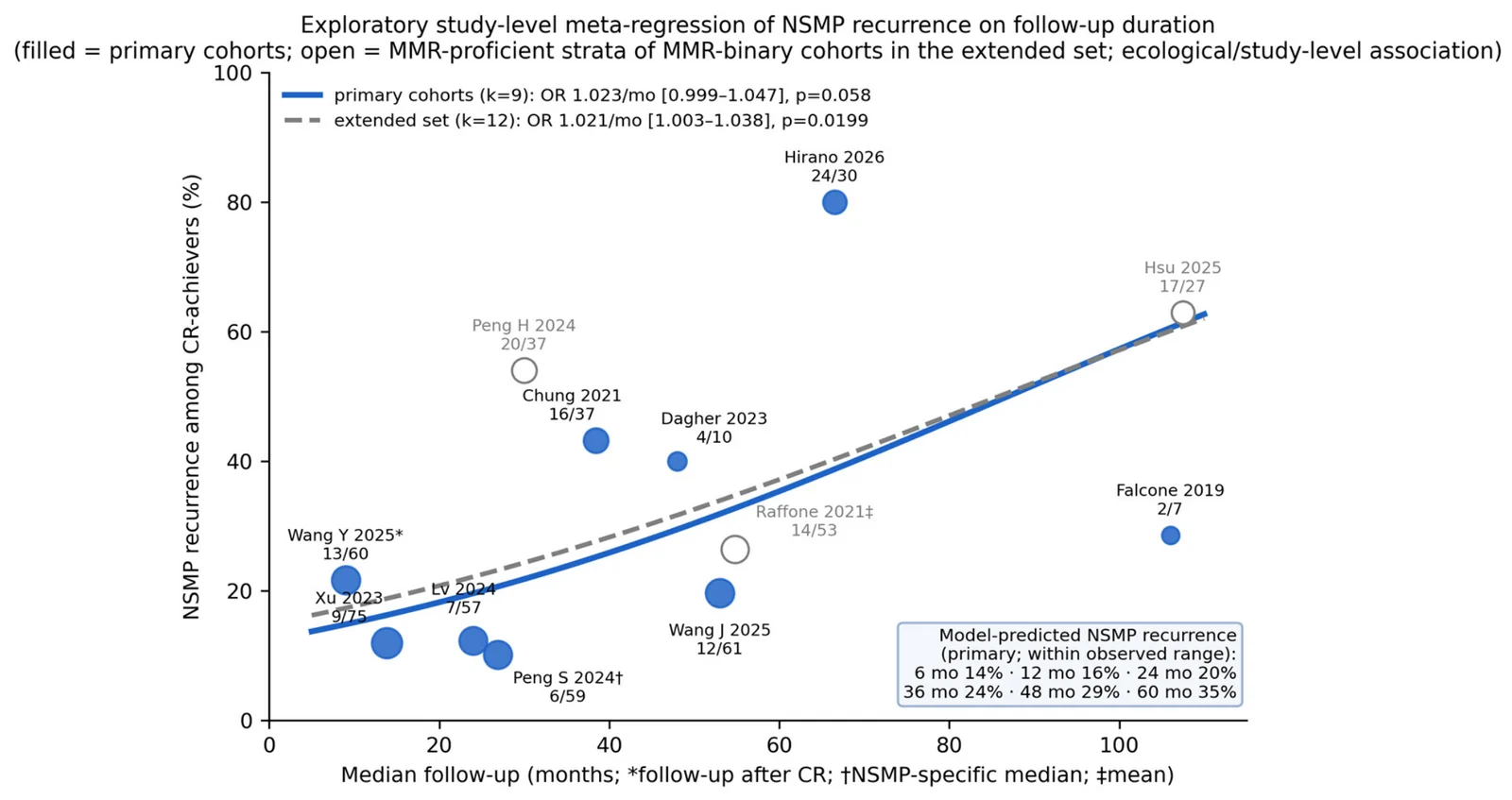
Exploratory study-level meta-regression of NSMP recurrence among CR-achievers on median follow-up (logit-GLMM). Filled markers and the solid line are the primary cohorts; open markers and the dashed line are the extended set including the MMR-proficient (p53-wild-type where tested) strata of the MMR-binary cohorts. Markers are sized by the number of CR-achievers; model predictions are shown within the observed follow-up range. Cohort labels refer to Falcone 2019 [28], Chung 2021 [29], Raffone 2021 [30], Dagher 2023 [31], Xu 2023 [32], Lv 2024 [33], Peng H. 2024 [34], Peng S. 2024 [35], Hsu 2025 [39], Wang J. 2025 [40], Wang Y. 2025 [41] and Hirano 2026 [16]. * Wang Y. 2025 [41]: follow-up after CR; † Peng S. 2024 [35]: NSMP-specific median follow-up; ‡ Raffone 2021 [30]: mean follow-up.

**Follow-up meta-regression (exploratory).** Across the nine primary cohorts with reported follow-up (9–106 months), NSMP recurrence among CR-achievers tended to increase with median follow-up (OR 1.023 per month, 95% CI 0.999–1.047; Wald *p* = 0.058; likelihood-ratio *p* = 0.09; residual τ = 1.02 → 0.80), with model-predicted recurrence of 14% at 6 months, 20% at 24 months, 24% at 36 months, 29% at 48 months and 35% at 60 months (Figure 5). The direction was positive in every leave-one-out analysis (OR 1.012–1.045 per month), but the strength of the association depended on the two cohorts followed beyond five years, which pull in opposite directions: without the cohort with the highest recurrence (24/30 at 66.5 months, ref. [16]) the slope flattened (OR 1.012, 95% CI 0.993–1.031; *p* = 0.21), whereas without the trial sub-study treated by hysteroscopic resection plus LNG-IUD, which had the longest follow-up and low recurrence (2/7 at 106 months, ref. [28]), it steepened (OR 1.045, 95% CI 1.015–1.076; *p* = 0.003), and without both it was OR 1.020 (95% CI 0.990–1.051; *p* = 0.19). In the extended set adding the MMR-proficient strata of the three MMR-binary cohorts with subtype-resolved recurrence (12 cohorts, 9–108 months), the association was OR 1.021 per month (95% CI 1.003–1.038; *p* = 0.020); it did not persist after excluding the nine-year cohort (*p* = 0.11, ref. [39]), or all cohorts followed beyond five years (*p* = 0.27). The association therefore rests on sparse long-term data and, being a study-level (ecological) association, should be regarded as hypothesis-generating. By contrast, MMRd recurrence showed no relationship with follow-up (OR 0.995 per month, 95% CI 0.974–1.016; *p* = 0.63; τ = 0). In the joint model, the follow-up-adjusted odds of recurrence for MMRd versus NSMP were 1.72 (95% CI 0.91–3.26; *p* = 0.09) in the primary set and 2.31 (95% CI 1.32–4.05; *p* = 0.003) in the extended set; the subtype-by-follow-up interaction was borderline in the primary set (ratio of follow-up ORs 0.976, 95% CI 0.952–1.001; Wald *p* = 0.06) and not significant in the extended set (Wald *p* = 0.18), the model-based MMRd-versus-NSMP contrast being larger at short follow-up (OR ≈ 3.2 at 12 months) than at 5 years (OR ≈ 1.0) in the primary set.

### 3.6. Disease Progression During Treatment

All ten primary cohorts reported progression during treatment by subtype, using heterogeneous definitions (progressive disease at a fixed-timepoint evaluation; any higher-grade lesion, myometrial invasion or extrauterine disease during treatment; appearance of more advanced disease; or progression as the initial-treatment outcome; Supplementary Table S4). Descriptively pooled progression was 3.8% (95% CI 1.9–7.4; 21/495) for NSMP, 20.4% (95% CI 12.9–30.7; 18/89) for MMRd, 6/39 for POLE (3/6, 1/2, 1/1, 1/1 and 0/1 to 0/11 in the remaining cohorts) and 4/24 for p53abn (Supplementary Figure S1). Progression among POLE-mutated tumours occurred mainly in the three cohorts with the most comprehensive sequencing (3/6, 1/1 and 1/1) [16,31,32], with one further case of progressive disease at the 6-month evaluation in a Sanger-classified cohort [29], and included histological upgrading from grade 1 to grade 2 within 6 months in one case. Because progression, persistent disease and recurrence were not uniformly distinguished across reports, these figures are descriptive; a quantitative synthesis of progression was pre-specified but was not performed in the original submission because of these definitional differences, and it is presented here as a post hoc analysis with that caveat.

### 3.7. p53-Abnormal Tumours (Descriptive)

Twenty-eight p53abn tumours were identified across seven primary cohorts (24 evaluable for response; Supplementary Table S5a,b). Best CR varied from 0/1 to 7/7 across cohorts (17 of 24 overall), CR at the early timepoint was 9/15, and recurrence after CR occurred in 9 of 17 patients (5/7, 1/3, 0/3, 0/1, 1/1 and 2/2 in the six cohorts reporting it); in the Zhengzhou cohort, p53abn status was independently associated with recurrence (multivariable hazard ratio 3.86, 95% CI 1.1–13.5) [40], and hysterectomy specimens in that cohort included a grade 2–3 endometrioid carcinoma and a synchronous stage IC grade 3 ovarian carcinoma. Progression during treatment was reported in 4 of 24. Reproductive outcomes were reported for a few p53abn patients: no live birth among five who attempted conception in one cohort [40], one pregnancy without live birth in the single patient who attempted conception in another [35], no conception attempts in a third [33], and one live birth after IVF in a fourth [41]. In the cluster-swap analysis, replacing the retained Zhengzhou cohort (7/7 CR; 5/7 recurrence) with the alternative report (3/9 CR; 3/3 recurrence; 4/9 progression) reversed the CR signal but not the durability concern. The limited evidence therefore raises concern about the oncologic durability of FST in p53abn disease, whereas response and reproductive outcomes remain insufficiently characterised.

### 3.8. Prevalence of Occult Higher-Risk Disease and Number Needed to Test

Among nine cohorts that classified all conventionally selected candidates before treatment (n = 663), pooled prevalence was 4.0% (95% CI 2.5–6.5; τ = 0.24; 28/663) for p53abn and 13.1% (95% CI 8.6–19.6; τ = 0.61; 89/663) for MMRd; combined, 17.0% (95% CI 11.7–24.0; prediction interval 6–40; leave-one-out 15–19%; 117/663), corresponding to a number needed to test of about 6 (95% CI 4–9). The combined prevalence varied widely across cohorts (7–47%; the highest value in the small trial sub-study in which only classifiable cases were counted), and was 15.2% (number needed to test about 7) after excluding the Zhengzhou cohort, the largest single contributor of MMRd cases (26 of 103 patients, 25%). Estimates including the grade-2-only and MMR-binary sensitivity cohorts were similar (p53abn 4.6%; MMRd 12.8%).

### 3.9. Sensitivity Analyses and Certainty of Evidence

**Figure 6:**
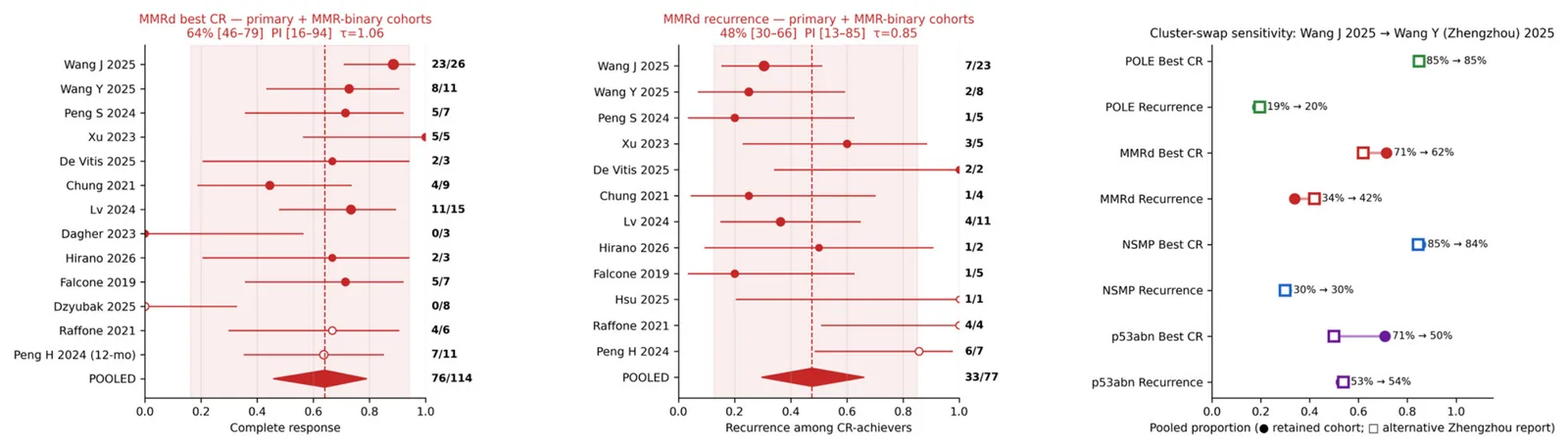
Sensitivity analyses. Left and centre: MMRd best CR and MMRd recurrence with the MMR-binary cohorts added (open markers: Raffone 2021 [30], Dzyubak 2025 [38] and Peng H. 2024 [34] for best CR; Raffone 2021 [30], Hsu 2025 [39] and Peng H. 2024 [34] for recurrence); the primary cohorts are those of Figure 2 and Figure 4. Right: cluster-swap analysis replacing the retained Zhengzhou cohort (Wang J. 2025 [40], filled circles) with the alternative Zhengzhou report (Wang Y. (Zhengzhou) 2025 [17], open squares); POLE and NSMP estimates are unchanged, whereas MMRd and p53abn estimates move (p53abn values are crude proportions). Diamonds denote pooled estimates with 95% confidence intervals; colours denote subtype (green, POLE; red, MMRd; blue, NSMP; purple, p53abn).

**MMR-binary expansion.** Adding the three MMR-binary cohorts with CR data lowered pooled MMRd best CR from 71.5% to 64.1% (95% CI 45.7–79.1; τ 0.58 → 1.06), driven by a standardised oncofertility programme with a stringent two-sampling CR definition and high baseline body-mass index in which no MMRd patient achieved CR (0/8) (Figure 6). Because CR definition, body-mass index, treatment pathway and patient selection differ jointly between that programme and the other cohorts, their individual contributions cannot be separated, and no cohort reported outcomes by subtype and body-mass index, so no formal BMI interaction analysis was possible; the pooled MMRd CR is best read as a range of roughly 60–70% that is sensitive to cohort-level characteristics. MMRd recurrence rose from 33.8% to 47.6% with the MMR-binary cohorts (Section 3.5), reflecting near-universal relapse in two small MMRd groups from cohorts consisting largely or entirely of atypical hyperplasia.

**Cluster swap.** Substituting the alternative Zhengzhou report left POLE and NSMP estimates unchanged (POLE CR 85% → 85%; NSMP CR 85% → 84%; NSMP recurrence 30% → 30%) but lowered MMRd best CR (71% → 62%) and raised MMRd recurrence (34% → 42%), and reversed the p53abn CR signal (Section 3.7).

**Leave-one-out and subgroups.** Best CR was robust for all subtypes (Section 3.3); recurrence was robust for MMRd (32–36%) and NSMP (25–34%) and, as expected, unstable for POLE (11–27%); occult prevalence was robust (Section 3.8). EC-only and mixed AEH/EC cohorts gave similar recurrence estimates for MMRd (38% vs. 32%) and NSMP (29% vs. 31%). Full leave-one-out results are given in Supplementary Table S6a–e.

**Certainty.** Under the GRADE framework for prognostic factors, certainty was low for the lower and slower CR of MMRd, for the elevated MMRd recurrence, and for the prevalence of occult p53abn/MMRd disease, and very low for the timing dependence of CR, the follow-up dependence of NSMP recurrence, POLE durability, and all p53abn findings (Table 2).

### 3.10. Reproductive Outcomes

Reproductive endpoints were reported heterogeneously and with different denominators (all treated patients, patients attempting conception or receiving fertility treatment, or pregnancies). In the cohorts reporting aggregate outcomes, 13–49% of all treated patients achieved at least one pregnancy [16,34,36,39], as did 43–56% of those attempting conception or receiving fertility treatment [35,38,40]; live births were reported for 9–18% of all patients [16,36,39] and 22–41% of those attempting conception [35,38,40], and 52–75% of pregnancies ended in a live birth [34,35,38,40]. Assisted reproduction accounted for most pregnancies where its use was reported [33,34,38,39]. Owing to this variation in denominators, follow-up and definitions, reproductive endpoints were summarised narratively rather than pooled. Subtype-specific reproductive outcomes were reported in only four primary cohorts and two sensitivity cohorts (in the AEH cohort, none of six p53abn patients and one of 11 MMRd patients had a live birth [34]); the sparse p53abn data from the primary cohorts are described in Section 3.7.

## 4. Discussion

### 4.1. Principal Findings

This systematic review and meta-analysis of 15 cohorts (10 in the primary analysis) shows that comparisons of fertility-sparing outcomes between molecular subtypes depend substantially on two timing constructs: outcome-assessment timing (when response is assessed) and follow-up duration (how long patients are followed). Three findings concern response. First, best/overall CR was high and similar for POLE-mutated (85%) and NSMP (85%) tumours and lower for MMRd tumours (71%), a difference that was consistent across sensitivity analyses and statistically significant within the paired cohorts (OR 0.36). Second, a fixed 6-month assessment understated eventual CR in every subtype: in the paired cohorts, 55% of MMRd and 76% of NSMP early non-responders subsequently achieved CR; the conditional probability of late response was, if anything, lower for MMRd, and no subtype-by-timepoint interaction was detected. MMRd tumours therefore responded less often and, where time to CR was reported, more slowly, but the data do not establish a distinct MMRd-specific delayed-response biology. Third, p53abn tumours showed unstable CR across cohorts. Three findings concern durability. MMRd recurrence after CR was elevated (about one third) and, unlike NSMP recurrence, unrelated to follow-up duration, so that the MMRd–NSMP difference is largest at short follow-up. NSMP recurrence tended to increase with follow-up in an exploratory study-level analysis that was of borderline significance and depended on which of the two cohorts followed beyond five years was included. POLE-mutated tumours did not recur in three cohorts followed for 14–24 months, but 4 of 10 relapsed within about two years in one cohort with short follow-up and 3 of 4 relapsed in the cohorts followed for three to five years (the single POLE patient in the trial sub-study, followed for 86 months, did not), so their durability under conservative management is uncertain. Finally, p53abn tumours recurred in 9 of 17 complete responders, and about one in six conventionally selected candidates carried an occult p53abn or MMRd tumour (range 7–47% across cohorts). All of these are prognostic associations within treated women; none establishes that any subtype benefits more or less from FST than from surgery.

### 4.2. Why Outcome-Assessment Timing and Effect-Measure Choice Matter

These findings illustrate why the choice of effect measure and the handling of timing are consequential in this literature. Because FST cohorts are single-arm, summarising CR as an odds ratio against the non-responders in the same cohort yields a quantity that is algebraically a proportion expressed as odds, not an interpretable treatment effect; pooling such quantities can also produce spuriously low heterogeneity, because the synthesised values are not genuinely comparable across studies. Equally, pooling CR without regard to assessment time, or comparing recurrence across cohorts with very different follow-up, conflates response kinetics and surveillance duration with biology. The genuine between-study heterogeneity we observed—τ = 1.6 for POLE recurrence and τ > 2 for POLE early CR—shows that apparent subtype gradients can be dominated by these factors. Synthesising proportions, decomposing best response into early and late components, and modelling follow-up explicitly exposes, rather than conceals, this heterogeneity and yields clinically interpretable estimates. However, decomposition of aggregate data cannot substitute for individual-patient time-to-event analyses, which remain the appropriate way to characterise response kinetics.

### 4.3. POLE-Mutated Disease: Highly Responsive but of Uncertain Durability

Our data qualify the common assumption that POLE-mutated EC is favourable on both axes under FST. That assumption conflates two phenomena measured on different time horizons. The first is supported: pooled best/overall CR was high (85%, 95% CI 70–93) and robust to leave-one-out and to the cluster swap. The second is not: the pooled POLE recurrence of 19% carried a prediction interval of 1–90% and is uninterpretable as a summary; resolving it by follow-up shows no recurrence in the three cohorts followed for 14–24 months, recurrence in three of four POLE complete responders in the cohorts followed for 38–53 months (including one relapse at 66 months after CR), no recurrence in the single POLE patient of the trial sub-study (followed for 86 months), and 4 of 10 relapses within about two years of CR in one cohort with short follow-up. The impression of low POLE recurrence therefore rests on short surveillance and very small numbers, and is consistent with the known tendency of POLE-mutated EC to relapse late.

Two factors compound the problem. POLE status determined by Sanger sequencing of exonuclease hotspots under-ascertains pathogenic mutations relative to broader panels, so several cohorts contain only one to three POLE cases, and the recurrence estimate is dominated by a handful of events. Moreover, those events are not cleanly attributable to POLE biology: in the Zhengzhou cohort, one of the two relapsing POLE tumours carried a concurrent p53 mutation—a multiple-classifier case assigned to the POLE group by convention—which the original investigators implicated in its poor prognosis [40], and progression during treatment among POLE-mutated tumours occurred mainly in the cohorts with the most comprehensive sequencing [16,31,32]. Under the hierarchical TCGA/WHO rules operationalised by ProMisE, a tumour harbouring both a pathogenic POLE mutation and abnormal p53 is classified as POLE-mutated because POLE ultramutation confers an excellent prognosis after standard surgery; in the conserved uterus, that override is untested. We therefore suggest that multiple-classifier (POLE-mutated/p53-abnormal) tumours be identified and reported separately in fertility-sparing series, so that the durability of “pure” POLE disease can be distinguished from that of co-altered tumours. Clinically, the excellent prognosis of POLE-mutated EC after hysterectomy should not be transferred uncritically to the conserved uterus: a favourable molecular label does not license early cessation of progestin or relaxed surveillance, and adequately followed POLE cohorts are needed before any de-escalation is considered.

### 4.4. MMRd: Less Frequent, Slower Response and Early Recurrence

The characterisation of MMRd tumours as poorly responsive derives largely from 6-month snapshots. When the same cohorts are read at best response, MMRd CR rises sharply but does not reach that of NSMP: within the paired cohorts, MMRd CR rose from 44% to 77% and NSMP CR from 57% to 90%, so that the absolute gap was similar at both timepoints (about 13 percentage points) and was statistically significant at best response (OR 0.36). Our conditional analysis clarifies the mechanism: the gain for MMRd reflects its lower early CR (a larger pool of early non-responders), whereas the probability that an early non-responder eventually achieved CR was lower for MMRd (55%) than for NSMP (76%). Together with the longer median times to CR reported for MMRd in two of three cohorts [35,41], the pragmatic reading is that MMRd is associated with a lower and slower response, so that a 6-month assessment exaggerates the deficit, and that roughly half of MMRd non-responders at 6 months will still respond with continued therapy. The magnitude of MMRd CR is also sensitive to cohort-level characteristics: in a standardised programme with a stringent two-sampling CR definition and high baseline obesity, no MMRd patient achieved CR [38]; because CR definition, body-mass index, treatment pathway and selection differ jointly between programmes, no single factor can be implicated. MMRd recurrence, by contrast, was the more dependable signal—about one third in the four-class cohorts, with an estimated between-cohort heterogeneity of zero (τ = 0; leave-one-out 32–36%), no relationship with follow-up, and a follow-up-adjusted excess over NSMP that was largest at short follow-up—and it was higher still (48%) when the MMR-binary cohorts, consisting largely or entirely of atypical hyperplasia, were added, consistent with a recent MMRd-focused meta-analysis reporting relapse in 41% [42]. Immune checkpoint inhibition, used in some MMRd patients in one cohort [41], is a further source of heterogeneity. MMRd patients may therefore be offered FST with realistic expectations of slower and less frequent response and a clear plan for intensified surveillance and timely escalation.

### 4.5. p53-Abnormal Disease

p53abn tumours were too few (n = 28) and too heterogeneous in their response for any pooled estimate: CR ranged from 0/1 to 7/7 across cohorts and reversed direction when the retained Zhengzhou cohort was exchanged for the alternative report. The more consistent signal was durability: recurrence after CR in 9 of 17 patients, an independent association with recurrence in the Zhengzhou cohort, high-grade or extrauterine disease at hysterectomy in several patients, and progression during treatment in 4 of 24. Reproductive outcomes were reported for very few p53abn patients (one live birth after IVF; no live birth among five attempting conception in one cohort and one pregnancy without live birth in another; no attempts in a fourth), so no conclusion about fertility can be drawn. Because p53abn tumours can present with grade 1 endometrioid morphology and minimal myometrial invasion—thereby satisfying conventional eligibility—molecular testing is the only means of identifying them before conservative management [43]. Pending prospective data, we regard p53abn status as a relative contraindication to FST that warrants explicit counselling about oncologic durability, rather than as an absolute exclusion; we did not test whether the p53abn associations depend on timing because the data do not permit it, and we no longer describe them as “timing-independent”.

### 4.6. NSMP Heterogeneity and Follow-Up

NSMP is the largest and, on both axes, the relatively favourable subtype, but only at matched follow-up. NSMP recurrence tended to rise with follow-up in our exploratory analysis (from about 14% at 6 months to about 35% at 5 years in the primary model), but the association was of borderline significance and its strength depended on the two cohorts followed beyond five years, which pull in opposite directions: a Japanese cohort treated with oral medroxyprogesterone acetate without maintenance therapy, in which 80% of NSMP responders had relapsed by a median of 5.5 years [16], and a small Italian trial cohort treated by hysteroscopic resection followed by a levonorgestrel-releasing intrauterine device, in which only two of seven NSMP responders had relapsed at a median of 9 years [28]. The contrast between these two cohorts is itself informative: it is compatible with a real, treatment-modifiable accrual of NSMP recurrence over time—hysteroscopic resection and intrauterine maintenance may lower the long-term recurrence propensity of a hormone-driven precursor state persisting in a conserved uterus—but a study-level association of this kind cannot be adjusted for cohort-level differences in treatment, maintenance therapy or surveillance intensity, and it is therefore hypothesis-generating. It is nonetheless consistent with the long-term single-cohort literature. NSMP patients require sustained surveillance and counselling, as initial response is not equivalent to cure. Emerging NSMP subclassification—estrogen-receptor expression and L1CAM [44]—may refine risk within this category and merits dedicated study.

### 4.7. Occult Higher-Risk Disease: Clinical Implications

Approximately one in six conventionally selected candidates carried an occult p53abn or MMRd tumour in the primary cohorts (combined prevalence 17%; number needed to test about 6, 95% CI 4–9), but this proportion varied from 7% to 47% across cohorts and depends on the population (AEH versus EC), the completeness of testing, and MMRd ascertainment; excluding the Zhengzhou cohort, which contributed the most MMRd cases, gave 15% (number needed to test about 7). Molecular profiling is thus most actionable not to reassure favourable subtypes into earlier de-escalation but to detect the minority whose tumour biology calls conservative management into question—recognising that MMRd status calls for counselling and surveillance rather than exclusion, and that the yield will differ between settings.

### 4.8. Comparison with Existing Literature

Our conclusions align with primary cohorts that, individually, show slower or less frequent MMRd response, elevated MMRd recurrence, and adverse p53abn behaviour, and with the subtype-stratified meta-analysis of Ferrari and colleagues [10], which pooled CR and recurrence at a single (unspecified) timepoint (CR 67%, 49%, 78% and 50%, and recurrence 14%, 43%, 18% and 33% for POLE, MMRd, NSMP and p53abn) and reached deliberately cautious conclusions because of small subtype samples. A meta-analysis restricted to MMRd tumours reported CR in 62% and relapse in 41% [42], and a more recent meta-analysis published after our search found MMRd to be the only subtype significantly associated with recurrence and reported non-significant recurrence odds for NSMP without modelling follow-up [45]. We are aware of no published meta-analysis that has examined NSMP recurrence as a function of follow-up duration; the low pooled NSMP recurrence in previous syntheses (about 18%) is what would be expected from cohorts followed for one to four years and is compatible with the follow-up dependence we describe, although our own data show that this dependence is not robust to the choice of long-follow-up cohorts. Where previous syntheses reported uniformly favourable POLE outcomes, our analysis indicates that such impressions reflect short follow-up and timepoint-blind pooling. By synthesising proportions, consolidating overlapping cohorts, decomposing best response into early and late components, and modelling follow-up, the present analysis clarifies that the favourable subtype signals are real for response but unproven for durability, and that the durability hierarchy is shaped by follow-up length.

### 4.9. Strengths and Limitations

**Strengths** include full-text data extraction from every primary report with public deposition of the dataset and its provenance, explicit consolidation of five overlapping reports, a proportion-based GLMM with exact handling of sparse cells validated against an established package, the separation of outcome-assessment timepoints with a conditional analysis that respects the cumulative nature of best response, formal subtype contrasts within cohorts, and explicit modelling of follow-up.

**Limitations of the evidence** are substantial. All evidence is retrospective, with attendant selection and ascertainment bias; in several cohorts molecular data were available only for selected patients, and in one the specimens were obtained largely after treatment. Subtype-specific samples—particularly POLE and p53abn—are small, so several estimates are imprecise. Molecular classification methods vary (next-generation sequencing to immunohistochemical surrogates; Sanger sequencing under-ascertains POLE; mutation-only or immunohistochemistry-only definitions of MMRd and p53abn), specimen timing was not always stated, and multiple-classifier tumours were handled by convention. Cohorts mixed AEH and EC (and, in some, grade 1 and 2 disease); because none reported subtype-resolved outcomes separately for AEH and EC, only cohort-level EC-only versus mixed comparisons were possible, and these showed no material differences. Denominators comprised all treated patients in most cohorts, but in one cohort only patients with at least 6 months of regular treatment were evaluable; the handling of early hysterectomy, discontinuation and loss to follow-up was rarely reported, and best response is vulnerable to unequal observation time. Because only aggregate data were available, individual-patient time-to-CR analyses—the appropriate way to characterise response kinetics—were not possible. Follow-up definitions differed (from treatment start in most cohorts, after CR in one, subtype-specific in one, and a mean rather than a median in one), and the follow-up meta-regression is a study-level association subject to ecological bias; its significance depended on the two cohorts followed beyond five years. Disease progression, persistent disease and recurrence were not uniformly distinguished; in one sensitivity cohort relapse after CR was subsumed under progressive disease and could not be extracted by subtype, and in another recurrence was not stratified by MMR status, so these cohorts contributed to CR but not to recurrence syntheses. Reproductive endpoints could not be synthesised quantitatively, and disease-specific and overall survival were rarely reported.

A further, and potentially important, source of unmeasured heterogeneity is the treatment protocol itself. The included cohorts combined oral progestins and levonorgestrel-releasing intrauterine systems, used hysteroscopic resection before or during medical therapy in a variable and often incompletely reported proportion (from none to all patients), and applied heterogeneous adjuncts (gonadotropin-releasing hormone agonists, letrozole, metformin, and, in one cohort, immune checkpoint inhibitors for MMRd disease). Hysteroscopic resection can accelerate and increase CR and may lower recurrence, so differences in its uptake could contribute to the between-study variability that our analysis attributes to measurement timing; the two long-follow-up cohorts that determine the NSMP follow-up association differ precisely in this respect (Section 4.6). Such an effect could plausibly interact with molecular subtype: debulking a lesion that responds slowly to progestin (as MMRd lesions appear to) could shorten time to CR and shift a 6-month assessment toward response, whereas cohorts relying on medical therapy alone would show a larger early deficit; conversely, resection may leave the recurrence propensity of MMRd or p53abn disease unchanged, so that resection-heavy cohorts would show a smaller response gap but a similar recurrence gap. Because subtype-specific treatment composition was not reported, a treatment-stratified analysis was not feasible, and residual confounding by treatment modality—including the possibility that part of the response heterogeneity we attribute to timing reflects resection uptake—cannot be excluded. Subtype-by-treatment interactions, especially the role of hysteroscopic resection, are a priority for individual-patient-data meta-analysis.

**Limitations of the review process** include the absence of prospective registration or a public time-stamped protocol (Section 2.1); the restriction to two databases without forward citation tracking; the fact that the database strings as executed were not archived, so that the strategy reported in Supplementary Table S1a is a reconstruction whose sensitivity was verified against the eligible reports but which cannot be assumed identical to the original searches; and the exclusion of series with fewer than 10 participants and of conference abstracts, which may disproportionately remove POLE and p53abn cases and small MMRd series and thus limit the precision of estimates for rare subtypes. The search was not updated beyond August 2025 (apart from one report identified by hand-searching before submission), and cohorts with subtype-resolved fertility-sparing outcomes have continued to appear since [46,47,48,49], so an updated synthesis will be warranted. Publication bias could not be assessed formally because of the small number of studies per subtype.

### 4.10. Clinical Implications and Future Directions

For clinical practice, molecular classification—mismatch-repair and p53 immunohistochemistry together with POLE sequencing—should be performed on the diagnostic biopsy of every fertility-sparing candidate, principally to detect the occult p53abn or MMRd tumours that conventional selection misses. Because the associations reported here are prognostic rather than predictive, a molecular result should recalibrate counselling and surveillance rather than by itself select or exclude patients from FST: p53abn status should be regarded as a relative contraindication pending prospective data; MMRd patients can be offered FST but should be counselled that response is less frequent and slower, with a plan for intensified surveillance and timely escalation rather than early abandonment; a POLE mutation should not be used to justify shorter progestin courses or relaxed monitoring; and NSMP patients should be told that initial response is not equivalent to cure. Finally, CR should be documented both at a fixed early timepoint and at best response, and recurrence reported together with follow-up duration, so that outcomes remain interpretable across studies.

For research, prospective, multicentre studies with pre-specified molecular stratification, standardised CR definitions, uniform specimen timing, and extended follow-up are the highest priority, ideally with individual-patient time-to-CR and time-to-recurrence data that can address treatment interactions (hysteroscopic resection, adjuncts) and the AEH-versus-EC question directly. Because CR criteria measurably affect subtype-specific estimates, a uniform definition, in particular the number of consecutive negative samplings required, should be agreed. Reporting of reproductive endpoints (conception attempts, use of assisted reproduction, live birth and miscarriage) should be standardised and mandated by subtype. Multiple-classifier tumours should be reported separately. Finally, the NSMP category warrants refinement using markers such as oestrogen-receptor expression and L1CAM, and adequately followed POLE cohorts are needed to characterise late recurrence directly rather than by inference from short-follow-up series.

## 5. Conclusions

Molecular classification carries prognostic information for the fertility-sparing management of endometrial cancer and atypical hyperplasia—prognostic within treated women, not predictive of benefit from FST relative to surgery—but the apparent gradients between subtypes are shaped substantially by when outcomes are assessed and how long patients are followed. A fixed 6-month assessment understates eventual CR in every subtype, and no subtype-by-timepoint interaction was detected; MMRd tumours respond less often and more slowly and recur early; NSMP recurrence tends to accrue with follow-up in an exploratory analysis whose strength depends on the few long-term cohorts and awaits confirmation; POLE-mutated tumours respond well, but their durability under conservative management is unproven; and the limited p53abn data raise concern about durability, so that p53abn status is best regarded as a relative contraindication pending prospective data. Roughly one in six conventionally selected candidates—varying widely between settings—harbours an occult p53abn or MMRd tumour. We therefore recommend molecular profiling at diagnostic biopsy to identify these tumours and to calibrate counselling and surveillance, and we caution against using a favourable molecular label, particularly POLE, to justify de-escalation. Establishing molecular-integrated fertility-sparing algorithms will require prospective, multicentre studies with standardised classification, uniform CR definitions, and extended follow-up.

## Figures and Tables

**Table 1:** Independent cohorts included after overlap resolution, ordered by year of publication and first author. Full characteristics (eligibility criteria, myometrial-invasion assessment, CR definitions, classifier details, specimen timing, follow-up definitions and denominators) are given in Supplementary Table S1a,b.

Cohort [Ref]	Country; Period	N (AEH/EC); Grade	Treatment; Hysteroscopic Resection (HR)	CR Assessment; Timepoints	Classifier; Specimen (Timing)	Median FU (mo)	Role in Analysis
Falcone 2019 [28]	Italy; 2007–2016	15 (0/15); IA G1 14, G2 1 (15 classifiable of 25 tested)	HR + LNG-IUD with adjuvant hormonal therapy (ECCo trial); HR in all	3-monthly hysteroscopy with biopsy; complete regression at 6 mo and at any time	ProMisE surrogate (MMR IHC, POLE/POLD1 NGS, p53 IHC); resection specimen (pre-progestin)	106	Primary (paired)
Chung 2021 [29]	South Korea; 2006–2018	57 (0/57); G1 52, G2 5	Oral MPA 500 mg/d (47%), MA 320 mg/d (9%) or MPA + LNG-IUS (44%); no HR	3-monthly D&C/hysteroscopic biopsy; 6 mo; best overall response	ProMisE (MMR IHC ± MSI PCR, p53 IHC, POLE Sanger exons 9/13); pre-treatment biopsy	38.4	Primary (paired)
Raffone 2021 [30]	Italy; 2004–2019	69 (47/22); G1	HR + LNG-IUD (36) or MA (33); HR in all	Regression (2 consecutive negative biopsies) within 12 mo; recurrence	MMR IHC only; pre-treatment (index) biopsy	54.8 (mean)	Sensitivity (MMR-binary)
Dagher 2023 [31]	USA; 2010–2021	20 (7/13); G1 11, G2 2	MA and/or LNG-IUD; no HR	3-monthly sampling; best response	MSK-IMPACT NGS (TCGA); mostly hysterectomy/progression specimen (post-treatment)	48	Primary (best CR, recurrence)
Xu 2023 [32]	China (Fudan); 2021–2022	90 (0/90); G1 88, G2 2	MA/MPA/LNG-IUS or GnRH-a + letrozole (21%); hysteroscopic lesion removal at evaluations	16 wk, 32 wk, overall	10-gene NGS + MSI PCR + p53 IHC; pre-treatment D&C	13.8	Primary (paired; 32-wk early timepoint)
Lv 2024 [33]	China; 2018–2021	93 (59/34); G1	LNG-IUS only; no HR	3-monthly hysteroscopy; overall CR	ProMisE (IHC + POLE NGS); FFPE (timing not explicit)	24 (2-y period)	Primary (best CR, recurrence)
Peng H 2024 [34]	China; 2015–2023	51 (51/0); AEH	Oral MA/MPA and/or LNG-IUS; no HR	3-monthly hysteroscopy/biopsy; cumulative CR at 3–12 mo; relapse and progression at 1–4 y	MMR and p53 IHC (binary); pre-treatment biopsy	30	Sensitivity (MMR-binary; AEH only)
Peng S 2024 [35]	China (Sichuan); 2012–2023	80 evaluable (0/80) of 116 classified; G1 (G2 7; superficial MI 12)	Oral MPA/MA (36%), LNG-IUS (19%), both (30%), GnRH-a + above (15%); no primary HR	3-monthly hysteroscopic evaluation; CR at 4, 8, 12 mo; overall CR; time to CR	ProMisE (MMR/p53 IHC, POLE NGS or Sanger); FFPE tumour tissue	22.0 (subtype-specific 12.4–26.9)	Primary (paired; 8-mo early timepoint)
Bogani 2025 [36]	Italy (7 centres); 2005–2024	23 (0/23); G2 only	LNG-IUD in all ± oral MA/MPA (7); HR 9/23	3-monthly hysteroscopic surveillance; 6 mo, 12 mo, best response (2 consecutive negative biopsies)	Surrogate 4-class (MMR/p53 IHC, POLE sequencing); FFPE (specimen not specified)	28.7	Sensitivity (grade 2 only)
De Vitis 2025 [37]	Italy; 2005–2021	33 (0/33) of 80 treated; G1	LNG-IUD + GnRH analogue (45%), + megestrol (30%), + megestrol + metformin (24%); no therapeutic HR	3-monthly curettage/hysteroscopic biopsy; 6 mo; best response	MMR/p53 IHC + NGS (POLE, TP53); archival FFPE	NR (treatment median 30)	Primary (paired)
Dzyubak 2025 [38]	Canada; 2019–2024	170 (107/63); G1 (p53 wild-type by design)	LNG-IUD 52 mg (162), oral MA (7) or HR only (1); HR 107/170	Two consecutive negative biopsies; time-to-CR	MMR IHC and POLE NGS in EC only (AH untested), p53 IHC; pre-treatment biopsy (implied)	18.8	Sensitivity (MMR-binary; CR only)
Hsu 2025 [39]	Taiwan; 2010–2021	40 (10/30); G1–2	Oral MPA/MA; no HR	3-monthly sampling; <6 mo, <12 mo, overall	MMR/p53/ARID1A IHC (no POLE); pre-treatment biopsy; tested only in CR-achievers	107.5	Sensitivity (MMR-binary; recurrence only)
Wang J 2025 [40]	China (Zhengzhou); 2006–2021	103 (66/37); G1	Oral MA/MPA ± LNG-IUS; no HR	3-monthly hysteroscopic sampling; 6 mo, 9 mo, overall	ProMisE (MMR/p53 IHC, POLE Sanger exons 9/13/14); pre-treatment D&C	53	Primary (paired; cluster-swap alternative available)
Wang Y 2025 [41]	China (Peking); 2020–2023	118 (8/110); G1 92, G2 18	HR in all + MPA/MA or GnRH-a + LNG-IUS; PD-1 inhibitor in 6 (5 MMRd)	3-monthly hysteroscopic biopsy; overall CR; time to CR	Targeted NGS (POLE, TP53, MSI); lesion tissue (pre-treatment)	9 (after CR)	Primary (best CR, recurrence)
Hirano 2026 [16]	Japan; 2016–2022	38 (20/18); G1	Oral MPA 600 mg/d after D&C; no HR	CR within 12 mo (total curettage)	143-gene NGS (2020 WHO criteria); MMRd/p53abn by mutation only; pre-treatment FFPE	66.5	Primary (best CR, recurrence)

AEH = atypical endometrial hyperplasia; EC = endometrial carcinoma; G = grade; HR = hysteroscopic resection; MA = megestrol acetate; MPA = medroxyprogesterone acetate; LNG-IUS/IUD = levonorgestrel-releasing intrauterine system/device; MI = myometrial invasion; IHC = immunohistochemistry; NGS = next-generation sequencing; FU = follow-up; NR = not reported. Primary = four-class-classified cohort of a standard fertility-sparing population; paired = reports CR at both a fixed early timepoint and best response for the same patients. Follow-up is measured from treatment start unless stated. Overlapping reports consolidated into these cohorts and the predominantly non-fertility-sparing cohort excluded are listed in Supplementary Table S2.

**Table 2:** Certainty of evidence (GRADE framework for prognostic factors; observational evidence starts at high certainty and is rated down for the domains shown).

Finding (Evidence Base)	Risk of Bias	Inconsistency	Indirectness	Imprecision	Certainty
Best CR lower in MMRd than NSMP (71% vs. 85%; within-cohort OR 0.36, 95% CI 0.17–0.79 in six paired cohorts); longer time to CR where reported (10 cohorts; 89 MMRd, 495 NSMP)	Serious	Not serious (τ = 0.58; direction consistent in 8/10 cohorts)	Not serious	Serious (wide CI; estimate sensitive to cohort-level characteristics)	Low
Fixed 6-month CR understates eventual CR in all subtypes; 55% (MMRd) and 76% (NSMP) of early non-responders later achieve CR; conditional late CR lower for MMRd (OR 0.43, 95% CI 0.18–1.04) (6 paired cohorts)	Serious	Not serious (best CR ≥ early CR in every stratum)	Serious (aggregate data; no individual time-to-CR)	Serious (few cohorts; wide CI)	Very low
MMRd recurrence elevated (34%, 95% CI 23–46; τ = 0; LOO 32–36%); FU-adjusted OR vs. NSMP 1.7 (0.9–3.3) primary, 2.3 (1.3–4.0) extended (9–12 cohorts)	Serious	Not serious (τ = 0)	Not serious	Serious (primary CI includes 1)	Low
NSMP recurrence rises with follow-up (OR 1.02/month; ≈14% at 6 mo to ≈35% at 5 y); borderline (*p* = 0.06) and dependent on which long-follow-up cohorts are included (9 cohorts)	Serious	Serious (residual τ = 0.80)	Serious (study-level, ecological)	Serious (CI includes 1; LOO *p* 0.003–0.21)	Very low
POLE: high best CR (85%, 95% CI 70–93) but recurrence uninterpretable as a single estimate (0/5 to 1/1 by cohort: no relapse in three cohorts followed 14–24 months or in one patient followed 86 months, but 4/10 within about two years in a short-follow-up cohort and 3/4 in cohorts followed 38–53 months) (10 cohorts; 39 patients)	Serious	Serious for recurrence (τ = 1.63)	Serious (Sanger under-ascertainment; multiple classifiers)	Very serious	Very low
p53abn: variable CR (0/1 to 7/7; 17/24), recurrence 9/17, progression 4/24, one live birth (after IVF) among 12 p53abn patients in the three cohorts reporting live births (7 cohorts; 28 patients)	Serious	Serious	Serious	Very serious	Very low
Occult p53abn or MMRd among conventionally selected candidates ≈17% (95% CI 12–24; range 7–47%; NNT ≈ 6) (9 cohorts; 663 candidates)	Serious (variable ascertainment)	Serious (range 7–47%)	Not serious	Not serious	Low

CR = complete response; FU = follow-up; LOO = leave-one-out; NNT = number needed to test; OR = odds ratio. Publication bias could not be assessed formally (few studies per subtype) and was not used to rate down; no upgrading criteria were applied (the follow-up–recurrence gradient was consistent in direction across leave-one-out analyses but not robust).

## Data Availability

The extracted dataset (with provenance for every value), the cohort-characteristics and overlap-resolution tables, the risk-of-bias assessments, the analysis code (Python), the validation script (R/metafor), the figure code and all results are openly available at https://github.com/naiad515/fst-endometrial-molecular-meta (accessed on 11 September 2026) and archived at Zenodo (DOI: 10.5281/zenodo.22006102; this version: 10.5281/zenodo.22006103).

## Supplementary Materials

The following supporting information can be downloaded at: https://www.mdpi.com/article/10.3390/cancers18182972/s1, Table S1a: Search strategy; Table S1b: Full characteristics of the included cohorts (one panel per cohort); Table S2: Overlap-resolution table (institutional clusters) and excluded related reports; Table S3. Risk of bias (QUIPS) by cohort; Table S4: Disease progression during treatment by molecular subtype, with each study’s definition; Table S5a: Paired cohorts: complete response at the early fixed timepoint (a), late CR among early non-responders (b of N − a) and best response (a + b of N); Table S5b: p53-abnormal tumours: per-cohort data; Table S6a: Leave-one-out analyses (pooled estimate after omitting each cohort); Table S6b: Meta-regression of recurrence on median follow-up: all analysis sets and leave-one-out; Table S6c: Validation of the Python adaptive-quadrature GLMM against metafor 4.4.0 (rma.glmm, ML, nAGQ = 25) and lme4 1.1-35 (glmer, nAGQ = 25) in R 4.3.3; Table S6d: Other sensitivity and subgroup analyses (pooled proportions, logit-GLMM); Table S6e: Post hoc comparison of the one-stage logit-GLMM (primary method) with two-stage DerSimonian–Laird pooling of logit proportions; Figure S1: Disease progression during fertility-sparing treatment by molecular subtype in the cohorts reporting it (Wilson 95% confidence intervals; event/total printed); the Pre-specified Analysis Plan (protocol, version 1.0 of 2 July 2026 with version history and a table distinguishing pre-specified from post hoc analyses); and the completed PRISMA 2020 checklist. References [50,51,52,53,54,55,56,57,58,59,60,61,62,63,64,65,66] are cited in the Supplementary Materials.

## Abbreviations

The following abbreviations are used in this manuscript:

AEH	Atypical endometrial hyperplasia
AGQ	Adaptive Gauss–Hermite quadrature
CI	Confidence interval
CR	Complete response
EC	Endometrial carcinoma
FST	Fertility-sparing treatment
GLMM	Generalized linear mixed model
GRADE	Grading of Recommendations, Assessment, Development, and Evaluations
IHC	Immunohistochemistry
LNG-IUS	Levonorgestrel-releasing intrauterine system
MMRd	Mismatch-repair–deficient
NGS	Next-generation sequencing
NNT	Number needed to test
NSMP	No specific molecular profile
OR	Odds ratio
p53abn	p53-abnormal
PI	Prediction interval
PRISMA	Preferred Reporting Items for Systematic Reviews and Meta-Analyses
QUIPS	Quality In Prognosis Studies
TCGA	The Cancer Genome Atlas
WHO	World Health Organization
