# Supplementary material for: Fertility-Sparing Treatment of Endometrial Cancer and Atypical Endometrial Hyperplasia by Molecular Classification: A Systematic Review and Meta-Analysis Accounting for Outcome-Assessment Timing and Follow-Up Duration
Source: Cancers (Basel). 2026 Sep 14;18(18):2972. doi: 10.3390/cancers18182972 (PMC13605972; doi:10.3390/cancers18182972)
Supplement: Supplementary file 1 [file cancers-18-02972-s001.zip › PRISMA_2020_Checklist.pdf]

PRISMA 2020 Checklist

Fertility-Sparing Treatment of Endometrial Cancer and Atypical Endometrial Hyperplasia by Molecular Classification: A Systematic Review and Meta-Analysis Accounting for Outcome-Assessment Timing and Follow-Up Duration

Locations refer to sections of the revised manuscript (R1). Reference: Page MJ, et al. BMJ 2021;372:n71.

Section and Topic	Item #	Checklist Item	Location Where Item Is Reported
**TITLE**			
	1	Identify the report as a systematic review.	Title
**ABSTRACT**			
	2	See the PRISMA 2020 for Abstracts checklist.	Abstract (structured: Background/Objectives, Methods, Results, Conclusions)
**INTRODUCTION**			
	3	Describe the rationale for the review in the context of existing knowledge.	Section 1 (Introduction)
	4	Provide an explicit statement of the objective(s) or question(s) the review addresses.	Section 1 (final paragraph: aims 1–3)
**METHODS**			
	5	Specify the inclusion and exclusion criteria for the review and how studies were grouped for the syntheses.	Section 2.2 (Eligibility criteria; primary vs sensitivity cohorts)
	6	Specify all databases, registers, websites, organisations, reference lists and other sources searched or consulted, and the date when each was last searched.	Section 2.3 (PubMed/MEDLINE and Embase, 1 Jan 2015–31 Aug 2025; backward reference searching; supplementary searches Aug 2026)
	7	Present the full search strategies for all databases, registers and websites, including any filters and limits used.	Supplementary Table S1a (reconstructed PubMed and Embase strategies with date limits, re-run yields and sensitivity checks; Section 2.3)
	8	Specify the methods used to decide whether a study met the inclusion criteria, including how many reviewers screened each record, whether they worked independently.	Section 2.3 (screened in duplicate; consensus)
	9	Specify the methods used to collect data from reports, including how many reviewers collected data, whether they worked independently.	Section 2.4 (two reviewers; re-verified during revision; provenance in deposited dataset)
	10a	List and define all outcomes for which data were sought. Specify whether all results that were compatible with each outcome domain were sought.	Sections 2.2 and 2.4 (CR at fixed early timepoint and best response; recurrence after CR; progression; reproductive outcomes)
	10b	List and define all other variables for which data were sought.	Section 2.4; Table 1; Supplementary Table S1b
	11	Specify the methods used to assess risk of bias in the included studies, including details of the tool(s) used and how many reviewers assessed each study.	Section 2.6 (QUIPS; two reviewers); Supplementary Table S3
	12	Specify for each outcome the effect measure(s) used in the synthesis or presentation of results.	Section 2.7 (proportions; ORs from mixed models for subtype contrasts)
	13a	Describe the processes used to decide which studies were eligible for each synthesis.	Sections 2.5 and 2.7 (overlap consolidation; primary vs sensitivity analysis sets)
	13b	Describe any methods required to prepare the data for presentation or synthesis, such as handling of missing summary statistics, or data conversions.	Sections 2.4 and 2.7 (recalculated numerators/denominators; exact binomial likelihood; derived values flagged)
	13c	Describe any methods used to tabulate or visually display results of individual studies and syntheses.	Section 2.7; Figures 2–6; Tables 1–2; Supplementary Tables S4–S6
	13d	Describe any methods used to synthesize results and provide a rationale for the choice(s). If meta-analysis was performed, describe the model(s), method(s) to identify heterogeneity, and	Section 2.7 (logit-GLMM, adaptive Gauss–Hermite quadrature; τ and prediction intervals; software

		software package(s) used.	versions; validation)
	13e	Describe any methods used to explore possible causes of heterogeneity among study results (e.g., subgroup analysis, meta-regression).	Section 2.7 (conditional late-response analysis and subtype-by-timepoint tests; follow-up meta-regression; subtype-by-follow-up interaction; EC-only vs mixed cohorts)
	13f	Describe any sensitivity analyses conducted to assess robustness of the synthesized results.	Section 2.7 (MMR-binary expansion; cluster swap; leave-one-out; grade-2 inclusion; ICI-cohort and 32-week-cohort exclusion)
	14	Describe any methods used to assess risk of bias due to missing results in a synthesis (arising from reporting biases).	Section 2.7 and 4.9 (not formally assessable; discussed narratively)
	15	Describe any methods used to assess certainty (or confidence) in the body of evidence for an outcome.	Section 2.7 (GRADE for prognostic factors); Section 3.9; Table 2
**RESULTS**			
	16a	Describe the results of the search and selection process, from the number of records identified to the number of studies included, ideally using a flow diagram.	Section 3.1; Figure 1
	16b	Cite studies that might appear to meet the inclusion criteria, but which were excluded, and explain why they were excluded.	Section 3.1; Figure 1; Supplementary Table S2 (overlapping and excluded related reports)
	17	Cite each included study and present its characteristics.	Table 1; Supplementary Table S1b
	18	Present assessments of risk of bias for each included study.	Section 3.2; Supplementary Table S3
	19	For all outcomes, present, for each study: (a) summary statistics for each group (where appropriate) and (b) an effect estimate and its precision, ideally using structured tables or plots.	Figures 2–6 (event/total counts and Wilson 95% CIs for every cohort); Supplementary Tables S4–S5
	20a	For each synthesis, briefly summarise the characteristics and risk of bias among contributing studies.	Sections 3.3–3.9
	20b	Present results of all statistical syntheses conducted. If meta-analysis was done, present for each the summary estimate and its precision (e.g., CI) and measures of statistical heterogeneity.	Sections 3.3–3.9; Figures 2–6 (pooled estimates, 95% CI, prediction intervals, τ)
	20c	Present results of all investigations of possible causes of heterogeneity among study results.	Sections 3.4, 3.5 and 3.9
	20d	Present results of all sensitivity analyses conducted to assess the robustness of the synthesized results.	Section 3.9; Supplementary Table S6
	21	Present assessments of risk of bias due to missing results (arising from reporting biases) for each synthesis assessed.	Section 4.9 (narrative)
	22	Present assessments of certainty (or confidence) in the body of evidence for each outcome assessed.	Section 3.9; Table 2
**DISCUSSION**			
	23a	Provide a general interpretation of the results in the context of other evidence.	Sections 4.1 and 4.8
	23b	Discuss any limitations of the evidence included in the review.	Section 4.9
	23c	Discuss any limitations of the review processes used.	Section 4.9
	23d	Discuss implications of the results for practice, policy, and future research.	Sections 4.10 and 5
**OTHER INFORMATION**			
	24a	Provide registration information for the review, including the register name and registration number, or state that the review was not registered.	Section 2.1 (not registered; stated as a methodological limitation)
	24b	Indicate where the review protocol can be accessed, or state that a protocol was not prepared.	Section 2.1 (analysis plan v1.0 dated 2 July 2026 provided as Supplementary Protocol with version history)
	24c	Describe and explain any amendments to information provided at registration or in the protocol.	Section 2.1 and Supplementary Protocol (pre-specified vs post hoc analyses)
	25	Describe sources of financial or non-financial support for the	Funding statement

		review, and the role of the funders or sponsors in the review.	
	26	Declare any competing interests of review authors.	Conflicts of Interest statement
	27	Report which of the following are publicly available and where they can be found: template data collection forms; data extracted from included studies; data used for all analyses; analytic code; any other materials used in the review.	Data Availability Statement (public repository: extracted dataset with provenance, cohort characteristics, overlap table, QUIPS ratings, analysis and figure code, results)
