# Supplementary material for: Fertility-Sparing Treatment of Endometrial Cancer and Atypical Endometrial Hyperplasia by Molecular Classification: A Systematic Review and Meta-Analysis Accounting for Outcome-Assessment Timing and Follow-Up Duration
Source: Cancers (Basel). 2026 Sep 14;18(18):2972. doi: 10.3390/cancers18182972 (PMC13605972; doi:10.3390/cancers18182972)
Supplement: Supplementary file 1 [file cancers-18-02972-s001.zip › Supplementary_Materials.pdf]

Supplementary Materials

Fertility-Sparing Treatment of Endometrial Cancer and Atypical Endometrial Hyperplasia by Molecular Classification: A Systematic Review and Meta-Analysis Accounting for Outcome-Assessment Timing and Follow-Up Duration

Alatawi A, Almaser S, Alanazi M, Alatawi A, Kim S. Cancers (manuscript cancers-4446521, revised version).

Contents: Table S1 (S1a search strategy; S1b full cohort characteristics); Table S2 (overlap resolution); Table S3 (QUIPS risk of bias); Table S4 (progression by subtype and definitions); Table S5 (paired-cohort data and p53abn data); Table S6 (leave-one-out results, software validation and a comparison with two-stage DerSimonian–Laird pooling); Figure S1 (progression by subtype); references cited in the Supplementary Materials (numbered as in the main text). The complete dataset and code are available in the public repository cited in the Data Availability Statement.

Table S1a. Search strategy

Databases: PubMed (MEDLINE) and Embase (Embase.com), 1 January 2015 – 31 August 2025 (original searches run in August 2025); no language restriction; reference lists of eligible studies and reviews searched backwards. The strategy combined concept blocks for (1) endometrial carcinoma/atypical hyperplasia, (2) fertility-sparing or conservative treatment, (3) hormonal (progestin/LNG-IUS) therapy and (4) molecular or genomic classification, using controlled vocabulary (MeSH/Emtree) and free-text terms.

Reconstruction and re-execution during revision. The database-specific strings as executed in August 2025 were not archived and could not be retrieved during revision. The strategy was therefore reconstructed from the concept blocks recorded in the protocol (version 1.0) and re-executed by the corresponding author on 19 August 2026 with the original date limits, and its sensitivity was verified against the 20 eligible reports (15 retained cohorts and 5 consolidated overlapping reports; 19 are indexed in PubMed and Embase, one—Wang Y et al., Gynecol Obstet Clin Med 2023 [63]—is not). Reconstruction A combines blocks 1, 2 and 4 (the hormonal-therapy concept is implicit in fertility-sparing treatment); reconstruction B additionally requires block 3, as the protocol describes. Reconstruction A retrieved every eligible report published within the search window in both databases and is reported as the reconstructed strategy; reconstruction B was less sensitive (it missed Wang J 2025 [40] and Xue 2023 [66] in PubMed and Wang J 2025 [40] in Embase). The record counts of the original searches (PubMed 219, Embase 280; Figure 1) cannot be reproduced exactly because the original strings differed from the reconstruction; the yields of the reconstruction are given below.

Database / strategy	Re-run yield (19 Aug 2026)	Eligible reports captured
PubMed — reconstruction A (blocks 1 AND 2 AND 4; publication date 1 Jan 2015 – 31 Aug 2025)	113 records	18 of the 18 PubMed-indexed eligible reports published within the window; the 19th (Hirano 2026 [16], published online 9 Nov 2025) lies outside the date limit
PubMed — reconstruction B (blocks 1 AND 2 AND 3 AND 4; same date limit)	59 records	16 of 18 (missed Wang J 2025 [40] and Xue 2023 [66])
Embase — reconstruction A (blocks 1 AND 2 AND 4; publication years 2015–2025)	419 records	18 of 18 (Hirano 2026 [16] carries publication year 2026); restricting further to records entered into Embase before 1 September 2025 (NOT [1-9-2025]/sd, approximating the original search date) gives 377 records and 14 of 18 — Wang J 2025 [40], Bogani 2025 [36] and Hsu 2025 [39] were entered into Embase after that date
Embase — reconstruction B (blocks 1 AND 2 AND 3 AND 4; publication years 2015–2025)	185 records	17 of 18 (missed Wang J 2025 [40])

Screening of the re-run yield. All records retrieved by reconstruction A in either database (PubMed 113; Embase 419, of which 21 were the eligible reports and their conference-abstract or correspondence duplicates) were screened by title, and by abstract or full text where the title suggested a fertility-sparing cohort with molecular data. No additional eligible cohort was identified. Records examined in detail and excluded were: a Lynch-syndrome series [50] (Gynecol Oncol Rep 2025;59:101768; carriers treated with progestins, n = 7, below the 10-participant threshold); Chaudhari et al. [51], Gynecol Oncol Rep 2023;49:101257 (Mirena vs Liletta; MMR status available only for a small grade-1 carcinoma subset and reported only as the proportion of responders who were MMR-proficient; not extractable); Jang et al. [52], Gynecol Oncol 2024;191:19-24 (recurrence risk factors after CR; no molecular classification); two Peking University People's Hospital immunohistochemistry series [53,54] (Arch Gynecol Obstet 2023;307:2025-2031; Oncol Lett 2023;25:52; ER/PR/Ki-67/PTEN/p53 expression without molecular classification, and overlapping the retained Peking cohort); and the long-term follow-up report of a phase II LNG-IUD trial [55] (Clin Cancer Res 2024; PMID 38922360; spatial transcriptomics of relapse; no subtype-resolved outcomes). The reports already listed in Table S2 as consolidated or ineligible were also retrieved: Zhai 2025 [56] (POLE subset, n = 9); Yang X 2025 [57] (immune checkpoint inhibitors, n = 4); Yang L 2025 [14]; Zakhour 2017 [15]; Puechl 2021 [58]; Catena 2022 [59]; van Gent 2016 [60]; and the Chinese-language reports of the Peking and Fudan programmes [61,62].

PubMed — reconstruction A (as run)

("Endometrial Neoplasms"[Mesh] OR "Endometrial Hyperplasia"[Mesh] OR (endometri*[tiab] AND (cancer*[tiab] OR carcinoma*[tiab] OR neoplasm*[tiab] OR adenocarcinoma*[tiab] OR malignan*[tiab] OR tumor*[tiab] OR tumour*[tiab] OR hyperplasia[tiab] OR "intraepithelial neoplasia"[tiab]))) AND ("Fertility Preservation"[Mesh] OR "Organ Sparing Treatments"[Mesh] OR "Conservative Treatment"[Mesh] OR fertility-sparing[tiab] OR "fertility sparing"[tiab] OR fertility-preserving[tiab] OR "fertility preserving"[tiab] OR "fertility preservation"[tiab] OR "conservative treatment"[tiab] OR "conservative management"[tiab] OR "conservative therapy"[tiab] OR conservatively[tiab] OR uterus-preserving[tiab] OR "uterine preservation"[tiab] OR uterus-sparing[tiab] OR uterine-sparing[tiab] OR "non-surgical"[tiab] OR nonsurgical[tiab]) AND ("DNA Mismatch Repair"[Mesh] OR "Microsatellite Instability"[Mesh] OR "Tumor Suppressor Protein p53"[Mesh] OR "Genes, p53"[Mesh] OR "DNA Polymerase II"[Mesh] OR "Genomics"[Mesh] OR "High-Throughput Nucleotide Sequencing"[Mesh] OR "molecular classification"[tiab] OR "molecular subtype"[tiab] OR "molecular subtypes"[tiab] OR "molecular subtyping"[tiab] OR "molecular subgroup"[tiab] OR "molecular subgroups"[tiab] OR "molecular class"[tiab] OR "molecular classes"[tiab] OR "molecular classifier"[tiab] OR "molecular profile"[tiab] OR "molecular profiles"[tiab] OR "molecular profiling"[tiab] OR "molecular characterization"[tiab] OR "molecular characteristics"[tiab] OR "molecular marker"[tiab] OR "molecular markers"[tiab] OR "molecular features"[tiab] OR "molecular alterations"[tiab] OR ProMisE[tiab] OR TCGA[tiab] OR "Cancer Genome Atlas"[tiab] OR "mismatch repair"[tiab] OR MMR[tiab] OR MMRd[tiab] OR dMMR[tiab] OR "microsatellite instability"[tiab] OR "microsatellite unstable"[tiab] OR MSI[tiab] OR "MSI-H"[tiab] OR p53[tiab] OR TP53[tiab] OR POLE[tiab] OR "polymerase epsilon"[tiab] OR NSMP[tiab] OR "no specific molecular profile"[tiab] OR "copy number"[tiab] OR "copy-number"[tiab] OR genomic*[tiab] OR "next-generation sequencing"[tiab] OR "next generation sequencing"[tiab] OR "gene panel"[tiab] OR "multigene panel"[tiab] OR sequencing[tiab] OR mutation*[tiab] OR "Lynch syndrome"[tiab]) AND ("2015/01/01"[Date - Publication] : "2025/08/31"[Date - Publication])

PubMed — reconstruction B (four AND-ed blocks; for comparison)

("Endometrial Neoplasms"[Mesh] OR "Endometrial Hyperplasia"[Mesh] OR (endometri*[tiab] AND (cancer*[tiab] OR carcinoma*[tiab] OR neoplasm*[tiab] OR adenocarcinoma*[tiab] OR malignan*[tiab] OR tumor*[tiab] OR tumour*[tiab] OR hyperplasia[tiab] OR "intraepithelial neoplasia"[tiab]))) AND ("Fertility Preservation"[Mesh] OR "Organ Sparing Treatments"[Mesh] OR "Conservative Treatment"[Mesh] OR fertility-sparing[tiab] OR "fertility sparing"[tiab] OR fertility-preserving[tiab] OR "fertility preserving"[tiab] OR "fertility preservation"[tiab] OR "conservative treatment"[tiab] OR "conservative management"[tiab] OR "conservative therapy"[tiab] OR conservatively[tiab] OR uterus-preserving[tiab] OR "uterine preservation"[tiab] OR uterus-sparing[tiab] OR uterine-sparing[tiab] OR "non-surgical"[tiab] OR nonsurgical[tiab]) AND ("Progestins"[Mesh] OR "Medroxyprogesterone Acetate"[Mesh] OR "Megestrol Acetate"[Mesh] OR "Levonorgestrel"[Mesh] OR "Intrauterine Devices, Medicated"[Mesh] OR "Hysteroscopy"[Mesh] OR progestin*[tiab] OR progestogen*[tiab] OR progesterone[tiab] OR progestational[tiab] OR medroxyprogesterone[tiab] OR megestrol[tiab] OR levonorgestrel[tiab] OR LNG-IUS[tiab] OR LNG-IUD[tiab] OR "intrauterine device"[tiab] OR "intrauterine devices"[tiab] OR "intrauterine system"[tiab] OR "intrauterine systems"[tiab] OR hysteroscop*[tiab] OR "hormonal therapy"[tiab] OR "hormone therapy"[tiab] OR "hormonal treatment"[tiab] OR "hormone treatment"[tiab] OR "endocrine therapy"[tiab]) AND ("DNA Mismatch Repair"[Mesh] OR "Microsatellite Instability"[Mesh] OR "Tumor Suppressor Protein p53"[Mesh] OR "Genes, p53"[Mesh] OR "DNA Polymerase II"[Mesh] OR "Genomics"[Mesh] OR "High-Throughput Nucleotide Sequencing"[Mesh] OR "molecular classification"[tiab] OR "molecular subtype"[tiab] OR "molecular subtypes"[tiab] OR "molecular subtyping"[tiab] OR "molecular subgroup"[tiab] OR "molecular subgroups"[tiab] OR "molecular class"[tiab] OR "molecular classes"[tiab] OR "molecular classifier"[tiab] OR "molecular profile"[tiab] OR "molecular profiles"[tiab] OR "molecular profiling"[tiab] OR "molecular characterization"[tiab] OR "molecular characteristics"[tiab] OR "molecular marker"[tiab] OR "molecular markers"[tiab] OR "molecular features"[tiab] OR "molecular alterations"[tiab] OR ProMisE[tiab] OR TCGA[tiab] OR "Cancer Genome Atlas"[tiab] OR "mismatch repair"[tiab] OR MMR[tiab] OR MMRd[tiab] OR dMMR[tiab] OR "microsatellite instability"[tiab] OR "microsatellite unstable"[tiab] OR MSI[tiab] OR "MSI-H"[tiab] OR p53[tiab] OR TP53[tiab] OR POLE[tiab] OR "polymerase epsilon"[tiab] OR NSMP[tiab] OR "no specific molecular profile"[tiab] OR "copy number"[tiab] OR "copy-number"[tiab] OR genomic*[tiab] OR "next-generation sequencing"[tiab] OR "next generation sequencing"[tiab] OR "gene panel"[tiab] OR "multigene panel"[tiab] OR sequencing[tiab] OR mutation*[tiab] OR "Lynch syndrome"[tiab]) AND ("2015/01/01"[Date - Publication] : "2025/08/31"[Date - Publication])

Embase (Embase.com) — reconstruction A (as run)

('endometrium cancer'/exp OR 'endometrium carcinoma'/exp OR 'endometrium hyperplasia'/exp OR ((endometri* NEAR/3 (cancer* OR carcinoma* OR neoplasm* OR adenocarcinoma* OR malignan* OR tumor* OR tumour* OR hyperplasia OR 'intraepithelial neoplasia')):ti,ab,kw)) AND ('fertility preservation'/exp OR 'conservative treatment'/exp OR ('fertility sparing' OR 'fertility-sparing' OR 'fertility preserving' OR 'fertility-preserving' OR 'fertility preservation' OR 'conservative treatment' OR 'conservative management' OR 'conservative therapy' OR conservatively OR 'uterus preserving' OR 'uterus-preserving' OR 'uterine preservation' OR 'uterus sparing' OR 'uterus-

sparing' OR 'uterine sparing' OR 'uterine-sparing' OR 'non-surgical' OR nonsurgical):ti,ab,kw) AND ('mismatch repair'/exp OR 'microsatellite instability'/exp OR 'protein p53'/exp OR 'genomics'/exp OR 'high throughput sequencing'/exp OR ('molecular classification' OR 'molecular subtype' OR 'molecular subtypes' OR 'molecular subtyping' OR 'molecular subgroup' OR 'molecular subgroups' OR 'molecular class' OR 'molecular classes' OR 'molecular classifier' OR 'molecular profile' OR 'molecular profiles' OR 'molecular profiling' OR 'molecular characterization' OR 'molecular characteristics' OR 'molecular marker' OR 'molecular markers' OR 'molecular features' OR 'molecular alterations' OR promise OR tcga OR 'cancer genome atlas' OR 'mismatch repair' OR mmr OR mmrd OR dmmr OR 'microsatellite instability' OR 'microsatellite unstable' OR msi OR 'msi-h' OR p53 OR tp53 OR pole OR 'polymerase epsilon' OR nsmp OR 'no specific molecular profile' OR 'copy number' OR 'copy-number' OR genomic* OR 'next generation sequencing' OR 'next-generation sequencing' OR 'gene panel' OR 'multigene panel' OR sequencing OR mutation* OR 'lynch syndrome'):ti,ab,kw) AND [2015-2025]/py

Variant approximating the original search date: append NOT [1-9-2025]/sd (records entered before 1 September 2025).

Embase — reconstruction B (four AND-ed blocks; for comparison)

('endometrium cancer'/exp OR 'endometrium carcinoma'/exp OR 'endometrium hyperplasia'/exp OR ((endometri* NEAR/3 (cancer* OR carcinoma* OR neoplasm* OR adenocarcinoma* OR malignan* OR tumor* OR tumour* OR hyperplasia OR 'intraepithelial neoplasia')):ti,ab,kw)) AND ('fertility preservation'/exp OR 'conservative treatment'/exp OR ('fertility sparing' OR 'fertility-sparing' OR 'fertility preserving' OR 'fertility-preserving' OR 'fertility preservation' OR 'conservative treatment' OR 'conservative management' OR 'conservative therapy' OR conservatively OR 'uterus preserving' OR 'uterus-preserving' OR 'uterine preservation' OR 'uterus sparing' OR 'uterus-sparing' OR 'uterine sparing' OR 'uterine-sparing' OR 'non-surgical' OR nonsurgical):ti,ab,kw) AND ('gestagen'/exp OR 'medroxyprogesterone acetate'/exp OR 'megestrol acetate'/exp OR 'levonorgestrel'/exp OR 'levonorgestrel releasing intrauterine system'/exp OR 'hysteroscopy'/exp OR (progestin* OR progestogen* OR progesterone OR progestational OR gestagen* OR medroxyprogesterone OR megestrol OR levonorgestrel OR 'lng-ius' OR 'lng ius' OR 'lng-iud' OR 'lng iud' OR 'intrauterine device' OR 'intrauterine devices' OR 'intrauterine system' OR 'intrauterine systems' OR hysteroscop* OR 'hormonal therapy' OR 'hormone therapy' OR 'hormonal treatment' OR 'hormone treatment' OR 'endocrine therapy'):ti,ab,kw) AND ('mismatch repair'/exp OR 'microsatellite instability'/exp OR 'protein p53'/exp OR 'genomics'/exp OR 'high throughput sequencing'/exp OR ('molecular classification' OR 'molecular subtype' OR 'molecular subtypes' OR 'molecular subtyping' OR 'molecular subgroup' OR 'molecular subgroups' OR 'molecular class' OR 'molecular classes' OR 'molecular classifier' OR 'molecular profile' OR 'molecular profiles' OR 'molecular profiling' OR 'molecular characterization' OR 'molecular characteristics' OR 'molecular marker' OR 'molecular markers' OR 'molecular features' OR 'molecular alterations' OR promise OR tcga OR 'cancer genome atlas' OR 'mismatch repair' OR mmr OR mmrd OR dmmr OR 'microsatellite instability' OR 'microsatellite unstable' OR msi OR 'msi-h' OR p53 OR tp53 OR pole OR 'polymerase epsilon' OR nsmp OR 'no specific molecular profile' OR 'copy number' OR 'copy-number' OR genomic* OR 'next generation sequencing' OR 'next-generation sequencing' OR 'gene panel' OR 'multigene panel' OR sequencing OR mutation* OR 'lynch syndrome'):ti,ab,kw) AND [2015-2025]/py

Table S1b. Full characteristics of the included cohorts (one panel per cohort)

Falcone 2019 [28]

Field	Value
Cohort	Falcone 2019
Reference	Eur J Obstet Gynecol Reprod Biol 2019;240:220-225 [28]
DOI	10.1016/j.ejogrb.2019.07.013
Country	Italy
Institution	Istituto Nazionale Tumori Fondazione G. Pascale, Naples
Design	Retrospective molecular sub-study of a prospective trial (ECCo, EudraCT 2010-018581-23)
Enrolment period	2007-2016
N total	15
N AEH	0
N EC	15
Histology / grade	EEC G1 (14) / G2 (1), stage IA intramucous
Stage; myometrial-invasion assessment	Stage IA (resectoscopic specimen)
Standard FST eligibility applied	Yes (trial criteria)
Treatment	Hysteroscopic resection (tumour, adjacent endometrium, underlying myometrium) + LNG-IUD 20 ug/d inserted at resection; adjuvant hormonal therapy per protocol 3-60 mo (agent per patient not tabulated)
Hysteroscopic resection	Yes (all; ECCo protocol)
CR definition	Complete regression: no EC/AH at follow-up sampling (3-monthly hysteroscopy with biopsy); persistent disease = no regression within 6 mo
CR timepoints reported	6 mo (80%); 9 mo (86.6%); overall
Molecular classifier	ProMisE surrogate (MMR IHC MLH1/MSH2/MSH6/PMS2, POLE exons 9-13 and POLD1 exons 7-12 by NGS, p53 IHC; MSI Idylla)

Specimen	Hysteroscopic resection specimen (FFPE whole sections)
Specimen timing	At start of FST (resection precedes progestin; implied by protocol)
Median follow-up (months)	106.0
Follow-up definition	Median 106 mo (24-134) from hysteroscopic resection
Outcome denominators	15 fully classifiable of 25 tested (30 ECCo patients; 22 with MMR IHC)
Role in analysis	Primary (distinct)
Population group	EC only
Notes	One p53-abnormal case assigned to MMRd (multiple classifier); 2 further POLE/POLD1 mutations in MMRd cases

Chung 2021 [29]

Field	Value
Cohort	Chung 2021
Reference	Am J Obstet Gynecol 2021;224(4):370.e1-370.e13 [29]
DOI	10.1016/j.ajog.2020.10.003
Country	South Korea
Institution	Yonsei University Health System (Severance Hospital)
Design	Retrospective single-centre
Enrolment period	2006-2018
N total	57
N AEH	0
N EC	57
Histology / grade	EEC grade 1 52 (91%), grade 2 5 (9%); FIGO IA/IB
Stage; myometrial-invasion assessment	Stage IA/IB on MRI (MI in 7/57: <= 1/2 in 6, > 1/2 in 1)
Standard FST eligibility applied	Extended (grade 2 and stage IB permitted)
Treatment	Oral MPA 500 mg/d (47%); MA 320 mg/d (9%); MPA + LNG-IUS (44%)
Hysteroscopic resection	No (diagnostic D&C 79% / hysteroscopic biopsy 21%)
CR definition	No hyperplasia/carcinoma on D&C or hysteroscopic biopsy every 3 months
CR timepoints reported	6 mo; best overall response (PR = 0, so best CR = best response)
Molecular classifier	ProMisE (MMR IHC; MSI PCR in 47 — 2/9 MMRd assigned by MSI-H with intact IHC; POLE Sanger exons 9 and 13; p53 IHC)
Specimen	Pre-treatment endometrial biopsy (tissue microarray)
Specimen timing	Before hormone therapy
Median follow-up (months)	38.4
Follow-up definition	Median 38.4 mo (2.9-163.5) from start of progestin
Outcome denominators	All 57
Role in analysis	Primary (distinct)
Population group	EC only
Notes	4/9 MMRd immediate hysterectomy for treatment failure, 3 upstaged (IB, G3, carcinosarcoma); POLE recurrence at 66 mo after CR

Raffone 2021 [30]

Field	Value
Cohort	Raffone 2021
Reference	Gynecol Oncol 2021;161(3):795-801 [30]
DOI	10.1016/j.ygyno.2021.03.029
Country	Italy
Institution	Two referral centres (University of Naples Federico II; Catholic University, Rome)
Design	Retrospective multicentre

Enrolment period	Jan 2004-Jul 2019
N total	69
N AEH	47
N EC	22
Histology / grade	AEH / G1 EEC
Stage; myometrial-invasion assessment	No MI/extrauterine disease on ultrasound/MRI and hysteroscopic resection specimen
Standard FST eligibility applied	Yes (age <= 45)
Treatment	Hysteroscopic resection + LNG-IUD (36) or megestrol acetate (33)
Hysteroscopic resection	Yes (all)
CR definition	Regression = no pre-treatment lesion on 2 consecutive follow-up hysteroscopic biopsies; resistance = no regression within 12 mo
CR timepoints reported	Regression within 12 mo; recurrence after regression
Molecular classifier	MMR IHC only (MLH1/MSH2/MSH6/PMS2 or MSH6/PMS2)
Specimen	Paraffin blocks of the pre-treatment (index) endometrial biopsy
Specimen timing	Before treatment
Median follow-up (months)	54.8
Follow-up definition	MEAN 54.8 +/- 34.4 mo (median not reported); follow-up < 1 year excluded
Outcome denominators	All 69
Role in analysis	Sensitivity only (MMR-binary)
Population group	Mixed AEH/EC
Notes	MMRd 6 (3 AEH/3 EEC): 4 regressed, all 4 recurred (12-39 mo)

Dagher 2023 [31]

Field	Value
Cohort	Dagher 2023
Reference	Gynecol Oncol 2023;179:180-187 [31]
DOI	10.1016/j.ygyno.2023.11.006
Country	USA
Institution	Memorial Sloan Kettering Cancer Center
Design	Retrospective single-centre
Enrolment period	Jan 2010-Jun 2021
N total	20
N AEH	7
N EC	13
Histology / grade	AEH 7; EEC G1 11; G2 2
Stage; myometrial-invasion assessment	No radiological myoinvasion (MRI preferred)
Standard FST eligibility applied	Yes (age <=45)
Treatment	MA 160-320 mg/d (25%); LNG-IUD (25%); both (50%)
Hysteroscopic resection	No
CR definition	Absence of cancerous or hyperplastic lesions on follow-up biopsy (3-monthly D&C/biopsy)
CR timepoints reported	Best response; median time to CR 9 mo (no fixed 6-mo rate)
Molecular classifier	MSK-IMPACT tumour-normal NGS; TCGA-surrogate (POLE, MSI-H, CN-H, CN-L)
Specimen	Mostly hysterectomy specimen (EC) or biopsy at progression (AEH)
Specimen timing	Largely after/at end of FST (selection caveat)
Median follow-up (months)	48.0
Follow-up definition	Overall follow-up
Outcome denominators	20 patients with NGS data and >= 6 mo follow-up
Role in analysis	Primary (distinct)

Population group	Mixed AEH/EC
Notes	Only patients with NGS data (mainly hysterectomised) included; specimen timing differs from other cohorts

Xu 2023 [32]

**Field**	**Value**
Cohort	Xu 2023
Reference	Front Oncol 2023;13:1282356 [32]
DOI	10.3389/fonc.2023.1282356
Country	China
Institution	Obstetrics and Gynecology Hospital of Fudan University
Design	Retrospective single-centre
Enrolment period	Jan 2021-Jan 2022 (follow-up to Dec 2022)
N total	90
N AEH	0
N EC	90
Histology / grade	EEC (88 G1, 2 G2); some progressed from AEH during FST
Stage; myometrial-invasion assessment	Endometrium-confined on enhanced MRI/TVS
Standard FST eligibility applied	Yes (age 18-45)
Treatment	MA 160 mg/d (69%); MPA; LNG-IUS; MA + LNG-IUS; GnRH-a + letrozole (21%); metformin/statin for metabolic disorders
Hysteroscopic resection	Lesion removal under hysteroscopy at each 3-monthly evaluation
CR definition	Absence of hyperplasia or cancer at hysteroscopic evaluation
CR timepoints reported	16 wk; 32 wk; overall (end of follow-up)
Molecular classifier	Targeted NGS (10 genes incl. POLE, TP53, MMR genes) + MSI PCR (Promega); TCGA-surrogate
Specimen	Pre-treatment diagnostic D&C FFPE
Specimen timing	Before FST
Median follow-up (months)	13.8
Follow-up definition	Median 59.8 weeks (19.1-104.0)
Outcome denominators	All 90 (13/115 excluded for insufficient DNA; 8 prior progestin > 1 mo; 4 transferred)
Role in analysis	Primary (retained, Fudan cluster)
Population group	EC only
Notes	1 MSI-H tumour with concurrent TP53 mutation; 32-week assessment used as early fixed timepoint (~7.4 mo)

Lv 2024 [33]

**Field**	**Value**
Cohort	Lv 2024
Reference	J Gynecol Oncol 2024;35(3):e27 [33]
DOI	10.3802/jgo.2024.35.e27
Country	China
Institution	Tongji Hospital, Wuhan
Design	Retrospective single-centre
Enrolment period	Feb 2018-Feb 2021
N total	93
N AEH	59
N EC	34
Histology / grade	AEH / EEC grade 1
Stage; myometrial-invasion assessment	Endometrium-confined on MRI

Standard FST eligibility applied	Yes (age <=40)
Treatment	LNG-IUS (Mirena) alone
Hysteroscopic resection	No (hysteroscopy with curettage for diagnosis and 3-monthly evaluation)
CR definition	Complete disappearance of lesions (no cancer or atypical hyperplasia)
CR timepoints reported	Overall CR (Kaplan-Meier cumulative CR curves; no fixed-timepoint rates)
Molecular classifier	ProMisE (IHC + POLE NGS)
Specimen	FFPE endometrial tissue
Specimen timing	Not explicit (diagnostic tissue implied)
Median follow-up (months)	24.0
Follow-up definition	2-year follow-up period (median not reported)
Outcome denominators	All 93 (patients with incomplete follow-up excluded by design)
Role in analysis	Primary (distinct)
Population group	Mixed AEH/EC
Notes	AEH/EC split from the flow diagram

Peng H 2024 [34]

Field	Value
Cohort	Peng H 2024
Reference	Gynecol Oncol 2024;186:154-160 [34]
DOI	10.1016/j.ygyno.2024.04.013
Country	China
Institution	Second Hospital of Hebei Medical University
Design	Retrospective single-centre
Enrolment period	Jan 2015-Nov 2023
N total	51
N AEH	51
N EC	0
Histology / grade	EAH only (age < 40)
Stage; myometrial-invasion assessment	
Standard FST eligibility applied	Yes (AEH)
Treatment	Oral MA or MPA and/or LNG-IUS (doses not reported); hysteroscopy and biopsy every 3 months
Hysteroscopic resection	Not described as treatment
CR definition	Absence of hyperplasia or cancer on endometrial specimen
CR timepoints reported	3, 6, 9, 12 mo cumulative CR; relapse and progression at 1-4 y
Molecular classifier	MMR IHC and p53 IHC (binary markers; 2 patients both)
Specimen	Pre-treatment endometrial biopsy
Specimen timing	Before hormone therapy
Median follow-up (months)	30.0
Follow-up definition	Median 30 mo (6-80) from treatment start
Outcome denominators	All 51; Table 2 denominators (MMRd 12, p53abn 7) exceed the text counts (11, 6) by one — text-consistent denominators used
Role in analysis	Sensitivity only (MMR-binary; AEH-only)
Population group	AEH only
Notes	DOI corrected during revision

Peng S 2024 [35]

Field	Value

Cohort	Peng S 2024
Reference	Gynecol Oncol 2024;191:240-248 [35]
DOI	10.1016/j.ygyno.2024.10.012
Country	China
Institution	West China Second Hospital of Sichuan University
Design	Retrospective single-centre (part from a prospective RCT, NCT03463252)
Enrolment period	Jan 2012-Sep 2023
N total	80
N AEH	0
N EC	80
Histology / grade	EEC G1 (G2 in 7; superficial MI in 12); 18 with high-risk features
Stage; myometrial-invasion assessment	FIGO 2009 stage I; no MI or superficial (<50%) MI on MRI/pathology
Standard FST eligibility applied	Extended (age <=40; G1-2; superficial MI allowed)
Treatment	Oral MPA 250-500 mg/d or MA 160-480 mg/d (36%); LNG-IUS (19%); both (30%); GnRH-a + above (15%)
Hysteroscopic resection	Hysteroscopic evaluation every 3 months with at least one repeat biopsy via hysteroscopic resection (no primary therapeutic resection described)
CR definition	Absence of endometrial lesions at 3-monthly hysteroscopic evaluation
CR timepoints reported	4, 8, 12 mo; overall CR; median time to CR
Molecular classifier	ProMisE (MMR IHC, p53 IHC, POLE NGS or Sanger exons 9-14)
Specimen	FFPE tumour tissue
Specimen timing	Not explicitly stated (diagnostic tissue implied)
Median follow-up (months)	22.0
Follow-up definition	Median 22.0 mo (6.3-107.1) overall; by subtype p53wt 26.9, MMRd 16.8, POLE 14.4, p53abn 12.4
Outcome denominators	80 evaluable of 116 classified (excluded: 3 irregular treatment, 33 follow-up < 6 mo)
Role in analysis	Primary (retained, Sichuan cluster)
Population group	EC only
Notes	148 treated; 116 classified (POLE 10, MMRd 10, p53abn 8, p53wt 88); paired at the 8-month timepoint

Bogani 2025 [36]

Field	Value
Cohort	Bogani 2025
Reference	Int J Gynecol Cancer 2025;35(10):102035 [36]
DOI	10.1016/j.ijgc.2025.102035
Country	Italy (7 referral centres)
Institution	Multi-institutional (IRCCS Istituto Nazionale Tumori Milan and others; Mayo Clinic author affiliation)
Design	Retrospective multicentre
Enrolment period	Jan 2005-Dec 2024
N total	23
N AEH	0
N EC	23
Histology / grade	EEC grade 2 only, apparent stage IA
Stage; myometrial-invasion assessment	Apparent stage IA (clinical/radiological); micro-invasion found at resection in some
Standard FST eligibility applied	No (grade 2 only)
Treatment	LNG-IUD 52 mg in all; + oral MA 160-320 mg/d or MPA 400-600 mg/d in 7 (30%)
Hysteroscopic resection	Yes in 9/23 (39%)
CR definition	CR required on 2 consecutive endometrial biopsies >= 3 months apart (3-monthly hysteroscopic evaluation)
CR timepoints reported	6 mo; 12 mo; best response
Molecular classifier	Surrogate 4-class (MMR and p53 IHC; POLE targeted sequencing)

Specimen	FFPE tissue blocks (specimen type not specified)
Specimen timing	Not stated
Median follow-up (months)	28.7
Follow-up definition	Median 28.7 mo (8.2-91.1)
Outcome denominators	All 23 starting FST
Role in analysis	Sensitivity only (grade-2 population)
Population group	EC only (grade 2)
Notes	Progression 10/23 during surveillance (PD as best response 3/23; 6 after CR, labelled progressive disease); relapse after CR by class not reported (>= 1 POLE and >= 1 NSMP CR patient developed EC after CR); hysterectomy 19/23; micro-invasion in 3/9 resection specimens

De Vitis 2025 [37]

Field	Value
Cohort	De Vitis 2025
Reference	Int J Gynecol Cancer 2025;35(1):100024 [37]
DOI	10.1016/j.ijgc.2024.100024
Country	Italy
Institution	European Institute of Oncology, Milan
Design	Retrospective single-centre
Enrolment period	Feb 2005-Jul 2021
N total	33
N AEH	0
N EC	33
Histology / grade	EEC grade 1, stage IA
Stage; myometrial-invasion assessment	Stage IA (institutional criteria); Table 1: MI < 50% on MRI in 3/24 and on US in 3/25 (>= 50% in 1/25)
Standard FST eligibility applied	Yes (grade 1, stage IA by institutional criteria)
Treatment	LNG-IUD + GnRH analogue (45%); LNG-IUD + megestrol (30%); LNG-IUD + megestrol + metformin (24%); median treatment 30 mo (IQR 10.5-69.5)
Hysteroscopic resection	No therapeutic resection (diagnostic hysteroscopic polypectomy 42% / hysteroscopic biopsy 42%)
CR definition	Absence of any hyperplastic or cancerous lesion on 3-monthly curettage/hysteroscopic biopsy
CR timepoints reported	6 mo (primary endpoint); best overall response
Molecular classifier	MMR and p53 IHC + NGS for POLE and TP53 (multiple classifiers: POLEmut-p53abn -> POLEmut; MMRd-p53abn -> MMRd)
Specimen	Representative FFPE tumour tissue blocks (specimen type not specified)
Specimen timing	Not explicitly stated (diagnostic tissue implied)
Median follow-up (months)	
Follow-up definition	Cohort median not reported (case-level follow-up 47-175 in Table 2; treatment median 30 mo)
Outcome denominators	33 classified of 80 treated (41%): 44 excluded for lack of tumour tissue, 3 for undeterminable POLE status; all 33 in denominators
Role in analysis	Primary (distinct)
Population group	EC only
Notes	One POLEmut/p53abn double classifier assigned to POLEmut; synchronous ovarian cancer in 5/33 (Table 1)

Dzyubak 2025 [38]

Field	Value
Cohort	Dzyubak 2025
Reference	Gynecol Oncol 2025;199:72-78 [38]
DOI	10.1016/j.ygyno.2025.06.015
Country	Canada
Institution	Princess Margaret Cancer Centre oncofertility programme, Toronto

Design	Retrospective programme cohort
Enrolment period	Jan 2019-May 2024
N total	170
N AEH	107
N EC	63
Histology / grade	AH / EEC grade 1 (p53 wild-type)
Stage; myometrial-invasion assessment	No MI or extrauterine disease on MRI (57%) or ultrasound (40%) (of 206 referred)
Standard FST eligibility applied	Yes (p53 wild-type required)
Treatment	LNG-IUD 52 mg (162) or oral MA 40-80 mg bid (7) or hysteroscopic resection only (1); standardized pathway
Hysteroscopic resection	Yes in 107/170 (63%)
CR definition	Two consecutive negative endometrial biopsies
CR timepoints reported	Time-to-CR (KM: 86.6% at 24 mo); overall CR 69%
Molecular classifier	EC only (87 referred EC): ER/PR, MMR IHC (MMRd 8/86 tested), p53 IHC (wild-type by design), POLE NGS (1/68); AH not tested
Specimen	Not stated (pre-treatment implied: p53 status was an eligibility criterion)
Specimen timing	Before FST (implied)
Median follow-up (months)	18.8
Follow-up definition	Median 18.8 mo (1.1-62.0)
Outcome denominators	170 treated of 206 referred (24 ineligible, 12 lost to follow-up); MMR-tested denominator among treated EC not reported
Role in analysis	Sensitivity only (MMR-binary; p53wt-only population)
Population group	Mixed AEH/EC
Notes	Median BMI 32.7 (206 referred); no MMRd patient achieved CR (0/8); recurrence 27/117 among all CR-achievers is not stratified by MMR status (descriptive only)

Hsu 2025 [39]

Field	Value
Cohort	Hsu 2025
Reference	Acta Obstet Gynecol Scand 2025;104(10):1994-2005 [39]
DOI	10.1111/aogs.70028
Country	Taiwan
Institution	National Taiwan University Hospital
Design	Retrospective single-centre
Enrolment period	Jan 2010-Dec 2021
N total	40
N AEH	10
N EC	30
Histology / grade	EIN 10; EEC G1-2 30
Stage; myometrial-invasion assessment	No MI on MRI/TVS
Standard FST eligibility applied	Yes (age <=45)
Treatment	Oral MPA 250-500 mg/d or MA 160-320 mg/d
Hysteroscopic resection	No
CR definition	No intrauterine malignancy or hyperplastic tissue on 3-monthly sampling
CR timepoints reported	<6 mo; <12 mo; overall (not by molecular status)
Molecular classifier	IHC only (MMR, p53, ARID1A); no POLE
Specimen	Diagnostic endometrial biopsy
Specimen timing	Before FST
Median follow-up (months)	107.5
Follow-up definition	Median follow-up
Outcome denominators	Molecular data only for 28 CR-achievers with >= 2 y follow-up

Role in analysis	Sensitivity only (MMR-binary; molecular data restricted to CR-achievers)
Population group	Mixed AEH/EC
Notes	CR by MMR status not estimable

Wang J 2025 [40]

Field	Value
Cohort	Wang J 2025
Reference	Biomol Biomed 2025;26(3):471-482 [40]
DOI	10.17305/bb.2025.12445
Country	China
Institution	First Affiliated Hospital of Zhengzhou University
Design	Retrospective single-centre
Enrolment period	2006-2021
N total	103
N AEH	66
N EC	37
Histology / grade	AEH / EEC grade 1
Stage; myometrial-invasion assessment	Endometrium-confined on MRI (expert TVS acceptable)
Standard FST eligibility applied	Yes (AEH or G1 EEC, no MI, age 18-40)
Treatment	Oral MA/MPA (60%); MA/MPA + LNG-IUS (37%); other (3%; GnRH-a + AI or TCM); metformin for PCOS/obese
Hysteroscopic resection	No (diagnostic hysteroscopy/curettage and 3-monthly hysteroscopic sampling only)
CR definition	Absence of AEH/EEC on hysteroscopic sampling every 3 months (single negative histology)
CR timepoints reported	6 mo; 9 mo; overall (any time)
Molecular classifier	ProMisE surrogate: MMR IHC (MLH1/MSH2/MSH6/PMS2), p53 IHC, POLE Sanger (exons 9, 13, 14)
Specimen	Pre-treatment diagnostic hysteroscopy/D&C FFPE
Specimen timing	Before FST
Median follow-up (months)	53.0
Follow-up definition	Median 53 mo (12-201) from start of progestin
Outcome denominators	All 103 treated patients (14/117 excluded for insufficient tissue)
Role in analysis	Primary (retained, Zhengzhou cluster)
Population group	Mixed AEH/EC
Notes	1 POLE-mutated tumour with concurrent p53 mutation assigned to POLE (multiple classifier)

Wang Y 2025 [41]

Field	Value
Cohort	Wang Y 2025
Reference	Curr Oncol 2025;32(6):317 [41]
DOI	10.3390/curroncol32060317
Country	China
Institution	Peking University People's Hospital
Design	Retrospective single-centre
Enrolment period	Jun 2020-Sep 2023 (follow-up to Dec 2023)
N total	118
N AEH	8
N EC	110
Histology / grade	AEH 8; EEC G1 92; EEC G2 18

Stage; myometrial-invasion assessment	Stage IA; no or superficial MI on MRI; ER/PR positive
Standard FST eligibility applied	Extended (G1-2; superficial MI allowed)
Treatment	Hysteroscopic resection in all, then MPA 250-500 mg/d or MA 160-320 mg/d, or GnRH-a + LNG-IUS; +/- metformin, letrozole; PD-1 inhibitor in 6 (5 MMRd, 1 POLE)
Hysteroscopic resection	Yes (all patients)
CR definition	Absence of hyperplasia/carcinoma on 3-monthly hysteroscopic biopsy (two pathologists)
CR timepoints reported	Overall CR; median time to CR (no fixed 6-mo rate)
Molecular classifier	Targeted NGS (POLE, TP53, MSI at 55 loci); TCGA-surrogate hierarchy POLE > MSI-H > TP53mut > NSMP
Specimen	FFPE endometrial lesion tissue (>30% tumour)
Specimen timing	Before FST (diagnostic tissue; implied)
Median follow-up (months)	9.0
Follow-up definition	Median follow-up after CR to last visit 9 mo (4-18.5); overall follow-up not reported
Outcome denominators	All 118 (2 still on treatment counted as non-CR)
Role in analysis	Primary (retained, Peking cluster)
Population group	Mixed AEH/EC (EC-predominant)
Notes	Immune checkpoint inhibitor used in 5/11 MMRd; very short follow-up

Hirano 2026 [16]

Field	Value
Cohort	Hirano 2026
Reference	Cancer Sci 2026;117(1):236-245 [16]
DOI	10.1111/cas.70240
Country	Japan
Institution	Keio University
Design	Retrospective single-centre
Enrolment period	Sep 2016-Aug 2022
N total	38
N AEH	20
N EC	18
Histology / grade	AEH 20; EEC G1 18
Stage; myometrial-invasion assessment	Stage IA without MI
Standard FST eligibility applied	Yes
Treatment	Oral MPA 600 mg/d after D&C; MPA stopped at pathological CR
Hysteroscopic resection	No (D&C removal of lesions before MPA)
CR definition	No hyperplasia/carcinoma on total endometrial curettage within 1 year
CR timepoints reported	CR within 12 mo (= best); median time to disappearance 7 mo
Molecular classifier	143-gene NGS panel (PleSSision-Rapid); WHO 2020 algorithm; MMRd and p53abn by mutation (no MMR IHC)
Specimen	Pre-treatment FFPE
Specimen timing	Before/at start of MPA
Median follow-up (months)	66.5
Follow-up definition	Median follow-up (overall)
Outcome denominators	38 patients with sufficient tumour DNA and consent
Role in analysis	Primary (distinct)
Population group	Mixed AEH/EC
Notes	No maintenance therapy after CR; high recurrence

Wang Y (Zhengzhou) 2025 [17]

Field	Value
Cohort	Wang Y (Zhengzhou) 2025
Reference	Front Oncol 2025;15:1506639 [17]
DOI	10.3389/fonc.2025.1506639
Country	China
Institution	First Affiliated Hospital of Zhengzhou University
Design	Retrospective single-centre
Enrolment period	Jan 2010-Dec 2022
N total	116
N AEH	71
N EC	45
Histology / grade	AEH / EC
Stage; myometrial-invasion assessment	
Standard FST eligibility applied	Yes
Treatment	MA 160-320 mg/d +/- metformin +/- LNG-IUS
Hysteroscopic resection	
CR definition	
CR timepoints reported	12 mo evaluation
Molecular classifier	ProMisE (IHC + POLE sequencing)
Specimen	
Specimen timing	
Median follow-up (months)	
Follow-up definition	
Outcome denominators	
Role in analysis	Overlapping (Zhengzhou cluster) — used only in cluster-swap sensitivity analysis
Population group	Mixed AEH/EC
Notes	Same institution/senior author/pathologist; enrolment nested in retained cohort

Table S2. Overlap-resolution table (institutional clusters) and excluded related reports

cluster	report	institution	enrolment	n	classifier	decision
Zhengzhou	Wang J, Jiang G, Yan S, ... Guo R. Biomol Biomed 2025;26(3):471-482 (10.17305/bb.2025.12445) [40]	First Affiliated Hospital of Zhengzhou University	2006-2021	103 (66 AEH/37 EC)	ProMisE (MMR/p53 IHC, POLE Sanger)	RETAINED — most complete subtype outcomes (6-mo, 9-mo, overall CR; recurrence; multivariable Cox; fertility)
Zhengzhou	Wang Y, Lai T, Chu D, Bai J, Yan S, Guo R. Front Oncol 2025;15:1506639 (10.3389/fonc.2025.1506639) [17]	Same institution; same senior author (Guo R) and pathologist (Yan S)	2010-2022	116 (71 AEH/45 EC)	ProMisE (IHC + POLE sequencing)	Consolidated (overlapping enrolment window and programme); used as the alternative report in the cluster-swap sensitivity analysis
Peking	Wang Y, Bo L, Fan X, ... Wang J. Curr Oncol 2025;32(6):317 (10.3390/curroncol32060317) [41]	Peking University People's Hospital	Jun 2020-Sep 2023	118 (8 AEH/110 EC)	Targeted NGS (POLE, TP53, MSI)	RETAINED — largest and most recent report of the programme with case-level outcomes
Peking	Wang Y, Kang N, Li L, ... Wang J. Gynecol Obstet Clin Med 2023;3(1):38-43 (10.1016/j.gocm.2023.01.006) [63]	Same institution/authors	Jun 2020-Dec 2022	52 (6 AEH/46 EC)	NGS (TCGA surrogate)	Consolidated (interim report of the same cohort)
Peking	Zhang X, Chen D, Zhao X, ... Wang J, Shen D. Pathol Res Pract 2023;241:154278 (10.1016/j.prp.2022.154278) [64]	Same institution/senior authors	2013-2021	59 (EEC/EIN)	NGS (TCGA algorithm)	Consolidated (partial overlap 2020-2021; earlier patients not separable by subtype-

						outcome)
Peking	Wang YQ et al. Zhonghua Fu Chan Ke Za Zhi 2022;57(9):692-700 (Chinese) [61] and Zhai Z et al. J Gynecol Oncol 2025;36(4):e101 (POLE subset, n=9) [56]	Same institution	2020-2021; 2020-2023	46; 9	NGS	Not counted as eligible reports (Chinese-language duplicate of the same cohort; n<10 subset) — listed for completeness
Sichuan	Peng S, Zheng Y, Liu J, et al. Gynecol Oncol 2024;191:240-248 (10.1016/j.ygyno.2024.10.012) [35]	West China Second University Hospital	2012-2023	80 EC (of 116 classified)	ProMisE (IHC + POLE sequencing)	RETAINED — full four-class outcomes
Sichuan	Ran X, Hu T, Li Z. Front Oncol 2022;12:810631 (10.3389/fonc.2022.810631) [65]	Same institution	2010-2020	13 institutional patients pooled with 93 published cases	ProMisE (IHC + Sanger POLE)	Consolidated (institutional patients nested in the retained window; pooled analysis double-counts published series)
Fudan	Xu Y, Zhao M, Zhang L, ... Chen X, Wang C. Front Oncol 2023;13:1282356 (10.3389/fonc.2023.1282356) [32]	Obstetrics & Gynecology Hospital of Fudan University	Jan 2021-Jan 2022	90 EC	NGS (10 genes) + MSI PCR + p53 IHC	RETAINED — pre-treatment classification with fixed-timepoint and overall outcomes
Fudan	Xue Y, Dong Y, Lou Y, ... Chen X. J Gynecol Oncol 2023;34(4):e53 (10.3802/jgo.2023.34.e53) [66]	Same institution/senior author	Apr 2017-Jul 2021	135 (82 EEC/53 EAH)	NGS (13-gene / ECG10 panels)	Consolidated (overlapping enrolment; PTEN-focused report)
Fudan	Shao WY et al. Zhonghua Fu Chan Ke Za Zhi 2023;58(10):742-754 (Chinese) [62]; Yang X et al. J Gynecol Oncol 2025;36(4):e59 (Lynch/ICI, n=4) [57]	Same institution	2021-2023; n/a	171; 4	NGS	Not counted as eligible reports (Chinese-language report of the same programme; non-progestin ICI series n<10) — listed for completeness

Also excluded (not an overlapping report): Puechl AM et al., Cancers 2021;13:2847 [58] (Duke LNG-IUS cohort; only 14/58 treated for fertility preservation — predominantly non-fertility-sparing population). Identified in supplementary searches during revision and not eligible: Yang L et al., Technol Cancer Res Treat 2025 [14] (three-class classification without POLE; CR reported only at 3 months); Zakhour M et al., BJOG 2017 [15] (MMR-binary; women ≤ 55 years, not restricted to fertility preservation); Catena U et al., Front Med 2022;9:948509 [59] (Lynch-syndrome work-up of six MMR-deficient patients from a multicentre MMR-immunohistochemistry series; MMR-only, likely overlapping the retained Raffone 2021 cohort [30]); van Gent MD et al., Int J Gynecol Cancer 2016;26:483-490 [60] (n = 11; Wnt/PI3K–Akt mutation profiling without POLE, MMR or p53 classification).

Table S3. Risk of bias (QUIPS) by cohort

Cohort	Participation	Attrition	Prognostic-factor measurement	Outcome measurement	Confounding	Analysis / reporting	Overall	Justification
Wang J 2025	Moderate	Moderate	Moderate	Low	Moderate	Low	Moderate	Consecutive single-centre series 2006-2021; 14/117 (12%) excluded for insufficient tissue; explicit eligibility. FU 12-201 mo, no explicit loss-to-follow-up statement. ProMisE surrogate on pre-treatment tissue; POLE Sanger limited to exons 9/13/14; one POLE/p53 multiple classifier assigned to POLE. Response by 3-monthly hysteroscopic sampling with explicit CR/PD definitions. Multivariable Cox for recurrence (BMI, regimen, duration); CR unadjusted. Complete subtype tabulation.
Wang Y 2025	Moderate	Moderate	Low	Low	High	Moderate	Moderate	Single-centre 2020-2023; 8 AEH/110 EC incl. 18 grade-2. Very short follow-up (median 9 mo after CR; 2 patients still on treatment). NGS classification (POLE/TP53/MSI) on lesion tissue. CR by 3-monthly hysteroscopic biopsy, two pathologists. Treatment differed by subtype (PD-1 inhibitor in 5/11 MMRd; GnRH-a/LNG-IUS second line); no adjustment. Univariable analyses only.
Peng S 2024	Moderate	Moderate	Moderate	Low	Moderate	Low	Moderate	Single centre 2012-2023: 148 treated, 116 classified, 80 analysed for efficacy (3 irregular treatment, 33 follow-up < 6 mo); exclusion differed by subtype (POLE 10->5, p53abn 8->4, MMRd 10->7, p53wt 88->64) with reasons not given by subtype; extended eligibility (G2, superficial MI). Median FU 22 mo overall but 14.4 (POLE) and 12.4 mo (p53abn). ProMisE (MMR/p53 IHC, POLE NGS or Sanger exons 9-14) on FFPE tumour tissue; specimen timing and multiple-classifier handling not stated. Uniform 3-monthly hysteroscopic evaluation with explicit CR/PR/SD/PD/recurrence definitions, two pathologists. Regimens tabulated by subtype (no significant differences); multivariable Cox for cumulative CR (MMRd, POLE, superficial MI, insulin resistance);

								recurrence/PD unadjusted. Complete n/N, KM and Cox.
Xu 2023	Moderate	Moderate	Low	Low	Moderate	Low	Moderate	Single-centre 2021-2022; includes EEC that progressed from AEH during FST; 13/115 excluded for insufficient DNA. Short follow-up (median 13.8 mo). NGS panel + MSI PCR + p53 IHC on pre-treatment tissue. Hysteroscopic evaluation every 3 months, explicit definitions. Regimen differed by subtype (GnRH-a + letrozole in 21%); multivariable Cox for CR adjusted for BMI/PIK3CA/regimen. Full tabulation at 16 wk, 32 wk and overall.
De Vitis 2025	High	Moderate	Moderate	Low	High	Moderate	High	Single centre 2005-2021: 33 of 80 treated patients (41%) classifiable (44 no tumour tissue, 3 POLE not determinable); included vs excluded compared only in a supplement. No cohort median follow-up (treatment median 30 mo; case-level follow-up 47-175). MMR/p53 IHC + NGS (POLE, TP53) with pre-specified multiple-classifier rules on archival FFPE (specimen timing not stated). Uniform 3-monthly biopsy with explicit CR/PR/SD/PD/recurrence definitions; 6-month and best response reported. Descriptive only; heterogeneous regimens over 16 years, no adjustment. Complete case-level tabulation, no CIs; very small subgroups (3/3/25/2).
Chung 2021	Moderate	Moderate	Moderate	Low	High	Moderate	Moderate	Single centre 2006-2018, 57 EC (grade 2 in 5; MI on MRI in 7); explicit criteria, no flow diagram. Follow-up from 2.9 mo; the only MMRd 6-month responder was lost to follow-up. ProMisE on pre-treatment biopsy tissue microarray, blinded pathologists; POLE Sanger exons 9 and 13 only; 2/9 MMRd assigned by MSI-H with intact IHC. 3-monthly pathological response with explicit definitions; hysterectomies for treatment failure counted as non-CR. Unadjusted (regimen, grade, MI, PgR); 4/9 MMRd immediate hysterectomy (informative censoring for recurrence). Pre-specified endpoints with CIs; POLE/p53abn descriptive.
Lv 2024	Moderate	Moderate	Moderate	Low	Moderate	Moderate	Moderate	Single-centre 2018-2021; patients with incomplete follow-up excluded by design; uniform LNG-IUS-only regimen (limits treatment confounding); ProMisE (IHC + POLE NGS) with sparse assay reporting; 3-monthly hysteroscopy with curettage; univariable comparisons only; no median follow-up (2-year period).
Dagher 2023	High	Moderate	High	Low	High	Moderate	High	Only patients with MSK-IMPACT data included (n=20 of a larger treated population); sequencing mostly on hysterectomy specimens (EC) or at progression (AEH) — selection toward non-responders and post-treatment specimen; response defined by 3-monthly sampling; no adjustment; univariable comparison (0/3 MMRd vs 10/16 NSMP).
Hirano 2026	High	Low	Moderate	Low	Moderate	Moderate	High	Selected on availability of sufficient tumour DNA and consent (38 of a larger MPA-treated population); long follow-up (median 66.5 mo). MMRd and p53abn defined by mutations only (no MMR IHC; MLH1-methylated MMRd not captured; TP53 by sequencing). CR = disappearance within 1 year on total curettage. Multivariable Cox for recurrence (histology, PIK3CA).
Falcone 2019	High	Low	Moderate	Low	High	Moderate	High	Molecular sub-study of a prospective trial (ECCo): 15 of 30 enrolled (25 tested) fully classifiable, failures mostly due to low tumour volume; no included/excluded comparison. All 15 followed >= 24 mo (median 106 mo), no loss to follow-up. ProMisE surrogate on whole sections of the resection specimen (MMR IHC, POLE/POLD1 NGS, p53 IHC), blinded pathologist, hierarchical assignment (3 multiple classifiers assigned MMRd). Protocol 3-monthly hysteroscopy with biopsy and explicit definitions; fixed 6-month outcome per case. No adjustment (BMI >= 30 in 3/7 MMRd vs 1/7 NSMP; the only G2 tumour was MMRd). Case-level tables; no tests or CIs; very small subgroups (1/7/0/7).
Bogani 2025	High	Moderate	Moderate	Moderate	High	Moderate	High	Multi-institutional grade-2 EC series 2005-2024 (n=23, 7 centres); availability of molecular data was an inclusion criterion; no screening flow. Follow-up 8.2-91.1 mo; text and flow-chart denominators differ at 12 and 18 months. Surrogate 4-class classification (MMR/p53 IHC, POLE sequencing) without assay, specimen or timing details. 3-monthly hysteroscopic surveillance with 6-month and best response by class; disease after CR labelled progressive disease and not reported by class. Heterogeneous physician-chosen treatment (LNG-IUD in all, oral progestin in 7, resection in 9), no adjustment. Full cross-tabulations but internal inconsistencies, no CIs.
Hsu 2025	High	Low	Moderate	Low	Moderate	Moderate	High	Molecular data available only for 28 CR-achievers with >=2 years of follow-up (CR by

								MMR status not estimable; recurrence cohort selected on survival to 2 years). IHC only (MMR, p53, ARID1A; no POLE). Long follow-up (median 107.5 mo). Multivariable Cox for recurrence.
Dzyubak 2025	Moderate	Moderate	Moderate	Low	High	Moderate	Moderate	Standardized oncofertility programme 2019-2024: 170 treated of 206 referred (24 ineligible, 12 lost to follow-up); MMR/p53/POLE tested in referred EC only (AH untested), baseline table for the 206 referred. Median FU 18.8 mo (1.1-62.0), Kaplan-Meier with censoring. MMR IHC (8/86 EC), p53 wild-type by design, POLE NGS (1/68); no 4-class classifier; specimen not stated. Stringent CR (two consecutive negative biopsies) with explicit definitions. Crude comparison; molecular status excluded from the Cox model; recurrence not stratified by MMR status. MMRd result reported as a single count (0/8 CR).
Raffone 2021	Moderate	Low	Moderate	Low	High	Moderate	Moderate	Two-centre series 2004-2019 (n=69; 47 AEH/22 G1 EEC), hysteroscopic resection + LNG-IUD or megestrol in all; consecutive with explicit criteria but exclusion of 'patients who underwent hysterectomy' ambiguous, no flow numbers. Follow-up >= 12 mo in all (mean 54.8 mo). Ad hoc MMR IHC on the index biopsy (4-protein panel in 33, MSH6/PMS2 in 36), outcome-blinded pathologists; only 6 MMRd. Pre-specified regression/resistance/recurrence definitions (2 consecutive negative biopsies). Crude relative risks; no adjustment for route (recurrence 52% MA vs 13% LNG-IUD), histology, BMI or centre. MMR-proficient counts derived from totals.
Peng H 2024	Moderate	Moderate	Moderate	Moderate	High	High	High	Single-centre EAH series 2015-2023 (n=51, age < 40); explicit criteria, no flow diagram. Median FU 30 mo (6-80); relapse/progression at fixed years as crude proportions without censoring. MMR and p53 IHC on pre-treatment biopsy (binary markers, no POLE; 2 patients both MMRd and p53abn; heterogeneous-loss assignment not stated). 3-monthly hysteroscopy/biopsy with CR defined as absence of hyperplasia/cancer. No adjustment; regimen not reported by marker. Multiple text-vs-table inconsistencies (Table 2 denominators exceed text counts by one; abstract 1-year relapse p-value corresponds to the 2-year value).

Ratings: Low / Moderate / High risk of bias (Hayden et al., Ann Intern Med 2013 [18]). All 15 cohorts were re-rated from the full text of the primary report during revision. Overall rating rule: High if two or more domains were rated High, or if study participation or outcome measurement was rated High; otherwise Moderate if any domain was Moderate; Low otherwise.

Table S4. Disease progression during treatment by molecular subtype, with each study's definition

Cohort	POLE	MMRd	NSMP (MMRp)	p53abn	Whole cohort	Definition of progression used
Wang J 2025	0/3	2/26	2/67	1/7	—	PD = increase in histopathological grade and cellular heterogeneity at the 6-month evaluation (fixed-timepoint response category; some patients later achieved CR)
Wang Y 2025	0/11	3/11	1/92	0/4	—	PD = progression to higher grade or progressive disease during treatment (Table 1 of the report)
Peng S 2024	0/5	1/7	0/64	0/4	—	PD = appearance of more advanced disease during FST (Table 2 of the report; the single MMRd case: myometrial invasion at 9.3 months)
Xu 2023	3/6	2/5	7/78	0/1	—	PD = higher histological grade, deep myometrial invasion and/or extrauterine lesions during FST (Table 1 of the report; some patients later achieved CR)
De Vitis 2025	0/3	0/3	2/25	1/2	—	PD at the 6-month evaluation = progression to a lesion of higher grade or clinically progressive disease (myometrial invasion, extra-uterine disease or lymph-node metastasis); PD as best response 0/3, 0/3, 3/25, 1/2
Chung 2021	1/2	2/9	1/45	0/1	—	PD at the 6-month evaluation (4 patients: 2 MMRd, 1 p53wt, 1 POLE)
Lv 2024	0/6	4/15	2/67	2/5	—	PD = increase in pathological grade or stage / increased cellular atypia (Table 3 of the report)
Dagher 2023	1/1	2/3	3/16	—	—	PD = higher-grade lesion or radiographic advancement (myoinvasion or measurable increase); for baseline AEH, low-grade EC on treatment = PD
Hirano 2026	1/1	1/3	3/34	—	—	PD = worsening of histological type or surgical stage compared with pre-treatment (initial-treatment outcome,

						within 1 year)
Falcone 2019	0/1	1/7	0/7	—	—	Progression = MMRd case 7, grade 3 with myometrial invasion at 3 months (case-level Table 2 of the report)
Bogani 2025	0/1	0/3	2/17	1/2	10/23	PD as best response (= PD at 6 months) by class; whole-cohort progression during hysteroscopic surveillance 10/23 (6 after CR, labelled progressive disease)
Peng H 2024	—	6/11	7/40	4/6	—	Progression to endometrial carcinoma by 4 years among all patients (Table 4 of the report; MMR-binary AEH cohort, sensitivity only)
Wang Y (Zhengzhou) 2025	1/10	6/16	8/81	4/9	—	PD at the 12-month evaluation (alternative Zhengzhou report; not in primary syntheses)

Descriptively pooled (10 primary cohorts; logit-GLMM): NSMP 3.8% (95% CI 1.9–7.4; 21/495); MMRd 20.4% (12.9–30.7; 18/89); POLE 6/39 (unstable; τ = 3.2); p53abn 4/24. Definitions differ (fixed-timepoint category vs any-time progression vs initial-treatment outcome), so these figures are descriptive.

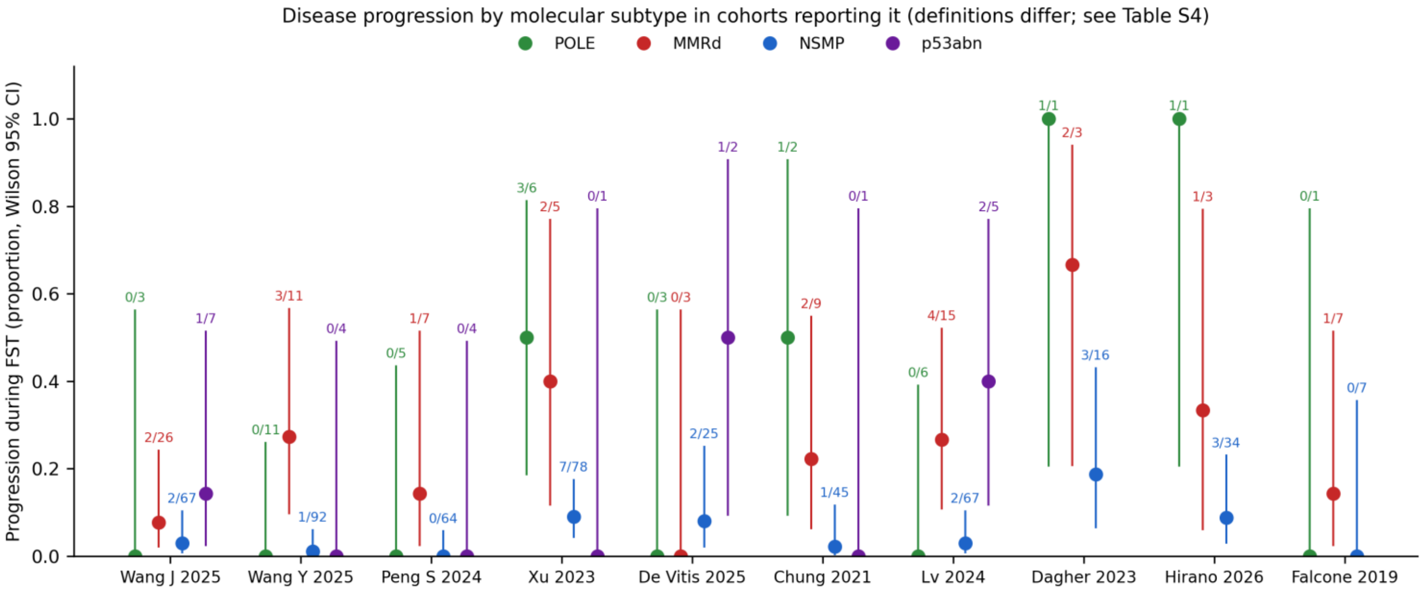

Figure S1. Disease progression during fertility-sparing treatment by molecular subtype in the cohorts reporting it (Wilson 95% confidence intervals; event/total printed).

Table S5a. Paired cohorts: complete response at the early fixed timepoint (a), late CR among early non-responders (b of N – a) and best response (a + b of N)

Cohort	Subtype	N	a (early CR)	Early CR	Late CR among early non-responders	Best CR	Early timepoint
Wang J 2025	POLE	3	3	3/3 (100%)	0/0 (—)	3/3 (100%)	6 mo
Wang J 2025	MMRd	26	16	16/26 (62%)	7/10 (70%)	23/26 (88%)	6 mo
Wang J 2025	NSMP	67	42	42/67 (63%)	19/25 (76%)	61/67 (91%)	6 mo
De Vitis 2025	POLE	3	0	0/3 (0%)	2/3 (67%)	2/3 (67%)	6 mo
De Vitis 2025	MMRd	3	0	0/3 (0%)	2/3 (67%)	2/3 (67%)	6 mo

De Vitis 2025	NSMP	25	6	6/25 (24%)	12/19 (63%)	18/25 (72%)	6 mo
Chung 2021	POLE	2	1	1/2 (50%)	0/1 (0%)	1/2 (50%)	6 mo
Chung 2021	MMRd	9	1	1/9 (11%)	3/8 (38%)	4/9 (44%)	6 mo
Chung 2021	NSMP	45	24	24/45 (53%)	13/21 (62%)	37/45 (82%)	6 mo
Xu 2023	POLE	6	3	3/6 (50%)	2/3 (67%)	5/6 (83%)	7.4 mo
Xu 2023	MMRd	5	3	3/5 (60%)	2/2 (100%)	5/5 (100%)	7.4 mo
Xu 2023	NSMP	78	45	45/78 (58%)	30/33 (91%)	75/78 (96%)	7.4 mo
Peng S 2024	POLE	5	5	5/5 (100%)	0/0 (—)	5/5 (100%)	8 mo
Peng S 2024	MMRd	7	3	3/7 (43%)	2/4 (50%)	5/7 (71%)	8 mo
Peng S 2024	NSMP	64	43	43/64 (67%)	16/21 (76%)	59/64 (92%)	8 mo
Falcone 2019	POLE	1	1	1/1 (100%)	0/0 (—)	1/1 (100%)	6 mo
Falcone 2019	MMRd	7	5	5/7 (71%)	0/2 (0%)	5/7 (71%)	6 mo
Falcone 2019	NSMP	7	6	6/7 (86%)	1/1 (100%)	7/7 (100%)	6 mo

Pooled (logit-GLMM): early CR — MMRd 43.7%, NSMP 57.0%, POLE 76.5%; best CR — MMRd 76.5%, NSMP 90.0%, POLE 85.0%; late CR among early non-responders — MMRd 55.2% (95% CI 37.2–71.9), NSMP 75.8% (64.4–84.5), POLE 57.1%. Mixed-model tests (subtype fixed effects, cohort random intercept): late CR MMRd vs NSMP OR 0.43 (95% CI 0.18–1.04; p = 0.06; LRT p = 0.12); early CR OR 0.58 (0.31–1.07); best CR OR 0.36 (0.17–0.79). Aggregate two-timepoint model (cohort × subtype strata; timepoints treated as independent samples): timepoint OR NSMP 7.20, MMRd 4.61, POLE 5.63; ratio MMRd/NSMP 0.64 (95% CI 0.23–1.81; p = 0.40). Median time to CR by subtype (months) where reported: Wang J 2025 — POLE 3.0, MMRd 4.0, p53abn 4.0, NSMP 4.0; Wang Y 2025 — 7, 11, 9, 6.5; Peng S 2024 — 3.0, 7.9, 3.5, 3.7; Dagher 2023 — NSMP 9; Hirano 2026 — 7 (all).

Table S5b. p53-abnormal tumours: per-cohort data

Cohort	n p53abn / N tested	Early CR	Best CR	Recurrence after CR	Progression	Live births	Notes
Wang J 2025	7/103	4/7	7/7	5/7	1/7	0/7	Table 2, 6-month response; Table 2, overall CR (any time); recurrence after CR; median FU 53 mo; PD at 6-month evaluation (fixed-timepoint response category); 0/5 attempting pregnancy; 0 live births; attempting
Wang Y 2025	4/118	—	3/4	1/3	0/4	1/4	Table 1 therapy outcomes (2 patients still on treatment counted as non-CR); recurrence after CR; median post-CR FU 9 mo (subtype-specific 5-10.5 mo); PD (progression to higher grade/progressive disease) during treatment,
Peng S 2024	8/116	3/4	3/4	0/3	0/4	0/1	among 116 molecularly classified FST patients (Results 3.1); 80 evaluable; Table 2: 8-month CR (second evaluation); 4-month CR 3/4; Table 2 overall CR; Table 2 RR among all who ever reached CR; median FU overall 22.0 mo
Xu 2023	1/90	0/1	0/1	—	0/1	—	CR at 32 weeks (Table 1 'CR rate 32w' row; an alternative Table 1 block gives POLE 4/6, NSMP 49/78); overall CR at end of follow-up (Table 1); progression during FST (higher grade/deep MI/extrauterine); Table 1
De Vitis 2025	2/33	1/2	1/2	0/1	1/2	—	Table 1: CR at 6 months (primary endpoint); Table 1: best overall response CR; Table 1: recurrence after CR (cohort median FU not reported; treatment median 30 mo); Table 1: PD at 6 months; PD as best response 1/2
Chung 2021	1/57	1/1	1/1	1/1	0/1	—	Table 2 / Table 3: CR at 6 months (POLE case 3 CR, case 15 PD; p53abn case 32 CR); best overall response (PR = 0 in cohort, so 'CR or PR' = CR): MMRd 4/9, p53wt 37/45; POLE 1/2 (case 15 never responded); p53abn 1/1; recu
Lv 2024	5/93	—	2/5	2/2	2/5	—	Table 3 overall CR; Table 3 recurrence after CR; 2-year follow-up period; Table 3 PD; no conception attempts (IVF-ET 0, natural 0)
Falcone 2019	0/15	—	—	—	—	—	ProMisE classes among 15 fully evaluable (p53 IHC abnormal in 1 MMRd case, assigned MMRd)
Dagher 2023	0/20	—	—	—	—	—	
Hirano 2026	0/38	—	—	—	—	—	

Bogani 2025	2/23	0/2	0/2	—	1/2	—	Table 2: 6-month CR; Table 2: best response CR (CR requires 2 consecutive negative biopsies); Table 2: PD as best response (whole-cohort progression during surveillance 10/23)
Peng H 2024	6/51	4/6	—	4/4	4/6	—	2 patients had both MMRd and p53abn; 12-month CR; Table 2 prints 4/7 but the text gives 6 p53abn of 51 and Table 3 lists 4 p53abn CR-achievers -> 4/6; Table 3: all relapsed by 2 years; Table 4: progression to EC by 4 yea
Wang Y (Zhengzhou) 2025	9/116	—	3/9	3/3	4/9	—	12-month CR (%): 90/37.5/33.33/85.19; recurrence %: 33.33/83.33/100/17.39; PD %: 10/37.5/44.4/9.9

Table S6a. Leave-one-out analyses (pooled estimate after omitting each cohort)

Synthesis	Full estimate	Leave-one-out range	Estimate omitting each cohort
Best CR POLE	84.6%	81.8–86.8%	Wang J 2025: 83.3%; Wang Y 2025: 82.2%; Peng S 2024: 82.4%; Xu 2023: 84.6%; De Vitis 2025: 86.1%; Chung 2021: 86.5%; Lv 2024: 81.8%; Dagher 2023: 86.8%; Hirano 2026: 86.8%; Falcone 2019: 84.2%
Best CR MMRd	71.5%	66.7–75.5%	Wang J 2025: 66.7%; Wang Y 2025: 70.9%; Peng S 2024: 71.2%; Xu 2023: 69.0%; De Vitis 2025: 71.6%; Chung 2021: 75.5%; Lv 2024: 70.7%; Dagher 2023: 75.2%; Hirano 2026: 71.6%; Falcone 2019: 71.2%
Best CR NSMP	85.3%	82.8–87.2%	Wang J 2025: 84.4%; Wang Y 2025: 87.2%; Peng S 2024: 84.1%; Xu 2023: 82.8%; De Vitis 2025: 86.4%; Chung 2021: 85.7%; Lv 2024: 85.4%; Dagher 2023: 86.7%; Hirano 2026: 85.3%; Falcone 2019: 84.5%
Recurrence POLE	18.7%	11.1–27.3%	Wang J 2025: 12.7%; Wang Y 2025: 11.1%; Peng S 2024: 26.1%; Xu 2023: 26.1%; De Vitis 2025: 12.8%; Chung 2021: 14.2%; Lv 2024: 27.3%; Falcone 2019: 20.6%
Recurrence MMRd	33.8%	31.7–35.7%	Wang J 2025: 35.7%; Wang Y 2025: 35.1%; Peng S 2024: 35.0%; Xu 2023: 31.7%; De Vitis 2025: 31.7%; Chung 2021: 34.4%; Lv 2024: 33.3%; Hirano 2026: 33.3%; Falcone 2019: 35.0%
Recurrence NSMP	30.3%	25.0–33.8%	Wang J 2025: 32.0%; Wang Y 2025: 31.6%; Peng S 2024: 33.8%; Xu 2023: 33.5%; De Vitis 2025: 25.7%; Chung 2021: 29.0%; Lv 2024: 33.3%; Dagher 2023: 29.6%; Hirano 2026: 25.0%; Falcone 2019: 30.7%
Occult p53abn	4.0%	3.5–4.7%	Wang J 2025: 3.5%; Wang Y 2025: 4.1%; Peng S 2024: 3.5%; Xu 2023: 4.7%; De Vitis 2025: 3.8%; Chung 2021: 4.4%; Lv 2024: 3.6%; Hirano 2026: 4.4%; Falcone 2019: 4.2%
Occult MMRd	13.1%	11.6–14.5%	Wang J 2025: 11.7%; Wang Y 2025: 13.8%; Peng S 2024: 13.9%; Xu 2023: 14.5%; De Vitis 2025: 13.6%; Chung 2021: 12.8%; Lv 2024: 12.7%; Hirano 2026: 13.8%; Falcone 2019: 11.6%
Occult combined	17.0%	15.2–18.9%	Wang J 2025: 15.2%; Wang Y 2025: 17.7%; Peng S 2024: 17.2%; Xu 2023: 18.9%; De Vitis 2025: 17.2%; Chung 2021: 16.9%; Lv 2024: 16.4%; Hirano 2026: 18.2%; Falcone 2019: 15.5%

Table S6b. Meta-regression of recurrence on median follow-up: all analysis sets and leave-one-out

Analysis set	k	OR per month (95% CI)	Wald p	LRT p	τ null → residual	FU range (mo)	Leave-one-out
Primary (9 cohorts)	9	1.023 (0.999–1.047)	0.0575	0.0905	1.02 → 0.80	9–106	Wang J 2025: OR 1.025 (p=0.047); Wang Y 2025: OR 1.026 (p=0.062); Peng S 2024: OR 1.021 (p=0.090); Xu 2023: OR 1.020 (p=0.139); Chung 2021: OR 1.023 (p=0.057); Lv 2024: OR 1.021 (p=0.104); Dagher 2023: OR 1.022 (p=0.079); Hirano 2026: OR 1.012 (p=0.211); Falcone 2019: OR 1.045 (p=0.003)
Primary excl. Wang Y (post-CR FU)	8	1.026 (0.999–1.054)	0.0617	0.0997	1.09 → 0.85	13.8–106	Wang J 2025: OR 1.027 (p=0.059); Peng S 2024: OR 1.022 (p=0.128); Xu 2023: OR 1.023 (p=0.176); Chung 2021: OR 1.027 (p=0.048); Lv 2024: OR 1.023 (p=0.137); Dagher 2023: OR 1.025 (p=0.082); Hirano 2026: OR 1.016 (p=0.143); Falcone 2019: OR 1.059 (p=0.001)
Primary excl. Hirano (66.5 mo)	8	1.012 (0.993–1.031)	0.2107	0.2352	0.54 → 0.47	9–106	Wang J 2025: OR 1.014 (p=0.199); Wang Y 2025: OR 1.016 (p=0.143); Peng S 2024: OR 1.011 (p=0.249); Xu 2023: OR 1.009 (p=0.381); Chung 2021: OR 1.009 (p=0.212); Lv 2024: OR 1.011 (p=0.283); Dagher 2023: OR 1.010 (p=0.298); Falcone 2019: OR 1.020 (p=0.189)
Primary excl. Falcone (106 mo)	8	1.045 (1.015–1.076)	0.0033	0.0133	1.07 → 0.68	9–66.5	Wang J 2025: OR 1.057 (p=0.000); Wang Y 2025: OR 1.059 (p=0.001); Peng S 2024: OR 1.042 (p=0.005); Xu 2023: OR 1.044 (p=0.013); Chung 2021: OR 1.044 (p=0.005); Lv

							2024: OR 1.043 (p=0.008); Dagher 2023: OR 1.045 (p=0.006); Hirano 2026: OR 1.020 (p=0.189)
Primary excl. Hirano and Falcone	7	1.020 (0.990–1.051)	0.1890	0.2017	0.56 → 0.49	9–53	Wang J 2025: OR 1.038 (p=0.067); Wang Y 2025: OR 1.033 (p=0.074); Peng S 2024: OR 1.019 (p=0.201); Xu 2023: OR 1.017 (p=0.358); Chung 2021: OR 1.009 (p=0.378); Lv 2024: OR 1.019 (p=0.242); Dagher 2023: OR 1.015 (p=0.351)
Extended (+ MMR-proficient strata of Hsu, Raffone, Peng H; 12 cohorts)	12	1.021 (1.003–1.038)	0.0199	0.0381	1.01 → 0.79	9–107.5	Wang J 2025: OR 1.021 (p=0.017); Wang Y 2025: OR 1.022 (p=0.031); Peng S 2024: OR 1.018 (p=0.036); Xu 2023: OR 1.018 (p=0.059); Chung 2021: OR 1.021 (p=0.019); Lv 2024: OR 1.019 (p=0.041); Dagher 2023: OR 1.020 (p=0.025); Hirano 2026: OR 1.017 (p=0.027); Falcone 2019: OR 1.027 (p=0.005); Hsu 2025: OR 1.019 (p=0.109); Raffone 2021: OR 1.021 (p=0.023); Peng H 2024: OR 1.023 (p=0.005)
Extended excl. Hsu (107.5 mo)	11	1.019 (0.996–1.043)	0.1086	0.1355	0.97 → 0.84	9–106	Wang J 2025: OR 1.021 (p=0.086); Wang Y 2025: OR 1.020 (p=0.138); Peng S 2024: OR 1.017 (p=0.160); Xu 2023: OR 1.016 (p=0.233); Chung 2021: OR 1.019 (p=0.113); Lv 2024: OR 1.017 (p=0.176); Dagher 2023: OR 1.019 (p=0.132); Hirano 2026: OR 1.010 (p=0.358); Falcone 2019: OR 1.037 (p=0.019); Raffone 2021: OR 1.020 (p=0.113); Peng H 2024: OR 1.022 (p=0.047)
Extended excl. all cohorts followed > 5 y	9	1.018 (0.986–1.050)	0.2698	0.2806	0.70 → 0.65	9–54.8	Wang J 2025: OR 1.026 (p=0.150); Wang Y 2025: OR 1.024 (p=0.247); Peng S 2024: OR 1.015 (p=0.333); Xu 2023: OR 1.012 (p=0.521); Chung 2021: OR 1.015 (p=0.339); Lv 2024: OR 1.015 (p=0.368); Dagher 2023: OR 1.015 (p=0.374); Raffone 2021: OR 1.022 (p=0.267); Peng H 2024: OR 1.019 (p=0.124)
MMRd recurrence vs FU (primary)	8	0.995 (0.974–1.016)	0.6273	0.6235	0.00 → 0.00	9–106	Wang J 2025: OR 0.995 (p=0.652); Wang Y 2025: OR 0.991 (p=0.450); Peng S 2024: OR 0.993 (p=0.513); Xu 2023: OR 0.999 (p=0.938); Chung 2021: OR 0.995 (p=0.625); Lv 2024: OR 0.996 (p=0.686); Hirano 2026: OR 0.993 (p=0.549); Falcone 2019: OR 0.999 (p=0.936)

Joint recurrence model (MMRd and NSMP; subtype, follow-up centred at 36 months, interaction; cohort random intercept). Primary set (9 cohorts, 17 subtype-observations): MMRd vs NSMP at 36 months OR 1.79 (95% CI 0.97–3.34); follow-up OR per month NSMP 1.023 (1.002–1.045), MMRd 0.999; interaction ratio 0.976 (0.952–1.001; Wald p = 0.06; LRT p = 0.06); additive model MMRd vs NSMP OR 1.72 (0.91–3.26; p = 0.093). Extended set (12 cohorts): additive MMRd vs NSMP OR 2.31 (1.32–4.05; p = 0.003); interaction p = 0.19.

Table S6c. Validation of the Python adaptive-quadrature GLMM against metafor 4.4.0 (rma.glmm, ML, nAGQ = 25) and lme4 1.1-35 (glmer, nAGQ = 25) in R 4.3.3

Model	k	Estimate (Python)	Estimate (R)	Δ	SE (Python)	SE (R)	Δ	τ (Python)	τ (R)	Δ
A1_best_cr_POLE_primary	10	1.70475	1.70475	-7.22e-09	0.44381	0.44381	-7.26e-09	0.00000	0.00000	+0.00e+00
A2_early_cr_POLE_primary	6	1.18109	1.18110	-4.77e-06	1.39746	1.39747	-1.04e-05	2.38473	2.38473	-3.69e-06
A4_recur_POLE_primary	8	-1.46784	-1.46784	-7.31e-07	0.89842	0.89843	-3.86e-07	1.63125	1.63126	-1.58e-06
A1_best_cr_MMRd_primary	10	0.91895	0.91895	-4.59e-06	0.32369	0.32369	+4.89e-07	0.58058	0.58058	+1.40e-06
A2_early_cr_MMRd_primary	6	-0.25409	-0.25409	-2.37e-06	0.47755	0.47755	-1.16e-06	0.78482	0.78482	-6.10e-06
A4_recur_MMRd_primary	9	-0.67016	-0.67016	+1.18e-08	0.26213	0.26213	+5.29e-09	0.00000	0.00000	+0.00e+00
A1_best_cr_NSMP_primary	10	1.75862	1.75861	+1.21e-05	0.28042	0.28042	+2.70e-06	0.73386	0.73385	+6.49e-06
A2_early_cr_NSMP_primary	6	0.28173	0.28173	+1.53e-06	0.24455	0.24455	-9.72e-07	0.48505	0.48505	-2.65e-06
A4_recur_NSMP_primary	10	-0.83099	-0.83099	+3.00e-06	0.39996	0.39996	+1.09e-06	1.17178	1.17178	+3.82e-06
A8_occult_p53abn_primary	9	-3.16530	-3.16530	-1.38e-06	0.25213	0.25213	+4.32e-07	0.24012	0.24012	+4.69e-06
A8_occult_MMRd_primary	9	-1.88995	-1.88995	+1.11e-06	0.24378	0.24378	-1.28e-06	0.61231	0.61231	-4.26e-06
A8_occult_combined	9	-1.58692	-1.58692	-1.65e-06	0.22279	0.22279	+5.90e-07	0.56138	0.56138	+1.96e-06
A1_MMRd_mmr_binary_expansion	13	0.57891	0.57891	-2.35e-06	0.38346	0.38347	-1.89e-06	1.06327	1.06328	-7.87e-06
A4_MMRd_mmr_binary_expansion	12	-0.09778	-0.09779	+8.29e-06	0.39273	0.39272	+3.17e-06	0.85250	0.85249	+7.86e-06
A5_metareg_primary_slope	9	0.02245	0.02245	-2.93e-07	0.01182	0.01182	+4.10e-09	0.80309	0.80309	-1.88e-06
A5_metareg_sens_all_slope	12	0.02032	0.02032	-5.46e-07	0.00873	0.00873	-1.84e-07	0.79018	0.79020	-2.42e-05
A3_lateCR_MMRd_vs_NSMP	6	-0.83567	-0.83567	-5.94e-07	0.44830	0.44830	+9.33e-08	—	0.45896	—

A5b_primary_interaction	9	-0.02408	-0.02408	+4.11e-08	0.01282	0.01282	+9.06e-09	—	0.71097	—

Software: Python 3.11.15, NumPy 2.4.4, pandas 3.0.2, SciPy 1.17.1, statsmodels 0.14.6, glmm_agq 1.2.0; R 4.3.3 with metafor 4.4.0 [23] and lme4 1.1-35 [24]. Estimates are on the logit scale (intercepts, slopes or log-odds ratios). Maximum absolute differences: estimate 1.2e-05, SE 1.0e-05, τ 2.4e-05.

Table S6d. Other sensitivity and subgroup analyses (pooled proportions, logit-GLMM)

Synthesis	Primary	+ grade-2-only cohort	EC-only cohorts	Mixed AEH/EC cohorts	Excl. ICI cohort	+ MMR-binary cohorts / other
Best CR — POLE	84.6% (69.7–92.9); τ 0.00; 33/39; k = 10	85.0% (70.4–93.1); τ 0.00; 34/40; k = 11	82.4% (57.3–94.2); τ 0.00; 14/17; k = 5	82.5% (9.1–99.5); τ 3.50; 19/22; k = 5	—	—
Early CR — POLE	76.5% (17.4–98.1); τ 2.38; 13/20; k = 6	81.1% (19.6–98.7); τ 2.39; 14/21; k = 7	—	—	—	excl. non-6-month cohorts: 71.0% (3.1–99.5); τ 3.19; 5/9; k = 4
Recurrence — POLE	18.7% (3.8–57.3); τ 1.63; 8/33; k = 8	— (no recurrence by class in the grade-2 cohort)	9.5% (0.2–83.9); τ 2.64; 2/14; k = 5	29.6% (8.1–66.7); τ 0.90; 6/19; k = 3	—	—
Best CR — MMRd	71.5% (57.1–82.5); τ 0.58; 65/89; k = 10	69.5% (54.7–81.2); τ 0.62; 66/92; k = 11	68.1% (47.4–83.4); τ 0.19; 21/31; k = 5	71.4% (46.4–87.8); τ 0.76; 44/58; k = 5	70.9% (53.7–83.7); τ 0.72; 57/78; k = 9	64.1% (45.7–79.1); τ 1.06; 76/114; k = 13
Early CR — MMRd	43.7% (23.3–66.4); τ 0.78; 28/57; k = 6	37.6% (17.1–63.8); τ 0.97; 28/60; k = 7	—	—	—	excl. non-6-month cohorts: 35.9% (9.7–74.4); τ 1.27; 22/45; k = 4
Recurrence — MMRd	33.8% (23.4–46.1); τ 0.00; 22/65; k = 9	— (no recurrence by class in the grade-2 cohort)	38.1% (20.3–59.8); τ 0.00; 8/21; k = 5	31.8% (19.8–46.8); τ 0.00; 14/44; k = 4	—	47.6% (29.6–66.2); τ 0.85; 33/77; k = 12
Best CR — NSMP	85.3% (77.0–91.0); τ 0.73; 413/495; k = 10	84.1% (75.7–90.0); τ 0.74; 424/512; k = 11	89.8% (79.2–95.3); τ 0.73; 196/219; k = 5	80.4% (68.6–88.5); τ 0.60; 217/276; k = 5	—	—
Early CR — NSMP	57.0% (45.1–68.2); τ 0.49; 166/286; k = 6	57.2% (47.0–66.8); τ 0.42; 176/303; k = 7	—	—	—	excl. non-6-month cohorts: 53.8% (34.0–72.4); τ 0.69; 78/144; k = 4
Recurrence — NSMP	30.3% (16.6–48.8); τ 1.17; 107/414; k = 10	— (no recurrence by class in the grade-2 cohort)	29.4% (11.6–56.9); τ 1.21; 47/196; k = 5	31.3% (13.6–56.9); τ 1.13; 60/218; k = 5	—	34.1% (21.3–49.6); τ 1.10; 158/531; k = 13

EC-only vs mixed AEH/EC cohorts, best CR (logit-GLMM with a subgroup fixed effect): POLE: OR 0.74 (95% CI 0.13–4.21; LRT p = 0.73); MMRd: OR 0.85 (95% CI 0.22–3.34; LRT p = 0.82); NSMP: OR 2.10 (95% CI 0.77–5.78; LRT p = 0.16). Recurrence subtype model (primary; subtype fixed effects, cohort random intercept): MMRd vs NSMP OR 1.80 (95% CI 0.97–3.35; p = 0.065); POLE vs NSMP OR 1.19 (p = 0.70).

Cluster-swap sensitivity analysis (Wang J 2025 replaced by the alternative Zhengzhou report): POLE best CR 84.8% (39/46), recurrence 19.6% (9/39); MMRd best CR 61.9% (48/79), recurrence 42.0% (20/48); NSMP best CR 84.4% (421/509), recurrence 30.0% (107/422); p53abn best CR 50.0% (13/26), recurrence 53.8% (7/13) (p53abn values are crude proportions).

Occult higher-risk disease (cohorts classifying all conventionally selected candidates before treatment): p53abn 4.0% (95% CI 2.5–6.5; 28/663); MMRd 13.1% (8.6–19.6; 89/663); combined 17.0% (11.7–24.0; PI 6–40; NNT 5.9, 95% CI 4.2–8.6); combined excluding Wang J 2025 15.2% (NNT 6.6); with the sensitivity cohorts p53abn 4.6%, MMRd 12.8%.

Table S6e. Post hoc comparison of the one-stage logit-GLMM (primary method) with two-stage DerSimonian–Laird pooling of logit proportions

Synthesis	k	Boundary cells (0 or all events)	One-stage logit-GLMM (95% CI)	Two-stage DerSimonian–Laird (95% CI)	Δ (pct points)
Best CR - POLE (primary)	10	6	84.6% (69.7–92.9); τ 0.0	74.9% (58.3–86.5); τ 0.0	-9.7
Best CR - MMRd (primary)	10	2	71.5% (57.1–82.5); τ 0.58	69.1% (56.8–79.2); τ 0.35	-2.4
Best CR - NSMP (primary)	10	1	85.3% (77.0–91.0); τ 0.73	83.4% (74.5–89.6); τ 0.73	-1.9
Early CR - MMRd (paired)	6	1	43.7% (23.3–66.4); τ 0.78	48.2% (30.3–66.6); τ 0.57	4.6
Early CR - NSMP (paired)	6	0	57.0% (45.1–68.2); τ 0.49	56.7% (45.7–67.1); τ 0.43	-0.3

Recurrence - MMRd (primary)	9	1	33.8% (23.4–46.1); τ 0.0	35.4% (25.1–47.4); τ 0.0	1.6
Recurrence - NSMP (primary)	10	0	30.3% (16.6–48.8); τ 1.17	31.3% (18.2–48.2); τ 1.07	0.9
Recurrence - POLE (primary)	8	5	18.7% (3.8–57.3); τ 1.63	34.8% (19.0–54.8); τ 0.32	16.1
Occult p53abn or MMRd (combined)	9	0	17.0% (11.7–24.0); τ 0.56	18.1% (12.6–25.4); τ 0.55	1.2

Two-stage analysis: logit-transformed proportions with a 0.5 continuity correction applied to every cohort (required for cohorts with zero or all events), inverse-variance pooling, DerSimonian–Laird τ^2; Wald confidence intervals on the logit scale, back-transformed (code: dl_comparison.py in the repository). The two approaches agree to within about 2 percentage points wherever boundary cells are absent or rare; for the sparse POLE syntheses (6/10 and 5/8 boundary cells) the continuity corrections pull the two-stage estimates toward 50% (best CR 84.6% → 74.9%; recurrence 18.7% → 34.8%) and collapse the estimated heterogeneity, illustrating why the exact-likelihood one-stage model was pre-specified as the primary method.

References cited in the Supplementary Materials (numbered as in the main text)

Reference numbers in Tables S1a, S1b, S2, S3 and S6c refer to the reference list of the main text; the entries cited in this file are reproduced here for convenience. References 50–66 are cited only in the Supplementary Materials.

14. Yang, L.; Nie, Y.; Guo, H. Comprehensive Genomic and Immunohistochemical Profiling to Predict Prognosis and Recurrence in Fertility-Sparing Therapy Based on Progesterone for Endometrial Carcinoma. Technol. Cancer Res. Treat. 2025, 24, 15330338251349972. https://doi.org/10.1177/15330338251349972

15. Zakhour, M.; Cohen, J.G.; Gibson, A.; Walts, A.; Karimian, B.; Baltayan, A.; Aoyama, C.; Garcia, L.; Dhaliwal, S.; Elashoff, D.; et al. Abnormal Mismatch Repair and Other Clinicopathologic Predictors of Poor Response to Progestin Treatment in Young Women with Endometrial Complex Atypical Hyperplasia and Well-Differentiated Endometrial Adenocarcinoma: A Consecutive Case Series. BJOG 2017, 124, 1576–1583. https://doi.org/10.1111/1471-0528.14491

16. Hirano, T.; Sakai, K.; Yoshimura, T.; Chiyoda, T.; Nakamura, K.; Aimono, E.; Kawaida, M.; Nishihara, H.; Susumu, N.; Aoki, D.; et al. Prognostic Biomarker of Fertility-Preserving Hormonal Therapy Based on Multigene Panel Testing for Endometrial Cancer. Cancer Sci. 2026, 117, 236–245. https://doi.org/10.1111/cas.70240

17. Wang, Y.; Lai, T.; Chu, D.; Bai, J.; Yan, S.; Guo, R. The Association of Molecular Classification with Fertility-Sparing Treatment of Atypical Endometrial Hyperplasia and Endometrial Cancer: A Retrospective Study. Front. Oncol. 2025, 15, 1506639. https://doi.org/10.3389/fonc.2025.1506639

18. Hayden, J.A.; van der Windt, D.A.; Cartwright, J.L.; Côté, P.; Bombardier, C. Assessing Bias in Studies of Prognostic Factors. Ann. Intern. Med. 2013, 158, 280–286. https://doi.org/10.7326/0003-4819-158-4-201302190-00009

23. Viechtbauer, W. Conducting Meta-Analyses in R with the metafor Package. J. Stat. Softw. 2010, 36, 1–48. https://doi.org/10.18637/jss.v036.i03

24. Bates, D.; Mächler, M.; Bolker, B.; Walker, S. Fitting Linear Mixed-Effects Models Using lme4. J. Stat. Softw. 2015, 67, 1–48. https://doi.org/10.18637/jss.v067.i01

28. Falcone, F.; Normanno, N.; Losito, N.S.; Scognamiglio, G.; Esposito Abate, R.; Chicchinelli, N.; Casella, G.; Laurelli, G.; Scaffa, C.; Greggi, S. Application of the Proactive Molecular Risk Classifier for Endometrial Cancer (ProMisE) to Patients Conservatively Treated: Outcomes from an Institutional Series. Eur. J. Obstet. Gynecol. Reprod. Biol. 2019, 240, 220–225. https://doi.org/10.1016/j.ejogrb.2019.07.013

29. Chung, Y.S.; Woo, H.Y.; Lee, J.Y.; Park, E.; Nam, E.J.; Kim, S.; Kim, S.W.; Kim, Y.T. Mismatch Repair Status Influences Response to Fertility-Sparing Treatment of Endometrial Cancer. Am. J. Obstet. Gynecol. 2021, 224, 370.e1–370.e13. https://doi.org/10.1016/j.ajog.2020.10.003

30. Raffone, A.; Catena, U.; Travaglino, A.; Masciullo, V.; Spadola, S.; Della Corte, L.; Piermattei, A.; Insabato, L.; Zannoni, G.F.; Scambia, G.; et al. Mismatch Repair-Deficiency Specifically Predicts Recurrence of Atypical Endometrial Hyperplasia and Early Endometrial Carcinoma after Conservative Treatment: A Multi-Center Study. Gynecol. Oncol. 2021, 161, 795–801. https://doi.org/10.1016/j.ygyno.2021.03.029

31. Dagher, C.; Manning-Geist, B.; Ellenson, L.H.; Weigelt, B.; Rios-Doria, E.; Barry, D.; Abu-Rustum, N.R.; Leitao, M.M.; Mueller, J.J. Molecular Subtyping in Endometrial Cancer: A Promising Strategy to Guide Fertility Preservation. Gynecol. Oncol. 2023, 179, 180–187. https://doi.org/10.1016/j.ygyno.2023.11.006

32. Xu, Y.; Zhao, M.; Zhang, L.; Wang, T.; Wang, B.; Xue, Y.; Xu, Z.; Shao, W.; Chen, X.; Wang, C. Outcomes of Fertility Preservation Treatments in Patients with Endometrial Cancer with Different Molecular Classifications Based on an NGS Panel. Front. Oncol. 2023, 13, 1282356. https://doi.org/10.3389/fonc.2023.1282356

33. Lv, X.; Guo, L.; Wang, C. Efficacy of Fertility-Sparing Treatment with LNG-IUS Is Associated with Different ProMisE Subtypes of Endometrial Carcinoma or Atypical Endometrial Hyperplasia. J. Gynecol. Oncol. 2024, 35, e27. https://doi.org/10.3802/jgo.2024.35.e27

34. Peng, H.; Jiang, J.; Li, L.; Hao, Z.; Lian, H.; Du, H.; Wang, W. Mismatch Repair Deficiency and Abnormal p53 Expression Has Significant Predictive Value for Progesterone Resistance and Endometrial Tumorigenesis in Patients with Endometrial Atypical Hyperplasia Receiving Fertility-Preserving Treatment. Gynecol. Oncol. 2024, 186, 154–160. https://doi.org/10.1016/j.ygyno.2024.04.013

35. Peng, S.; Zheng, Y.; Liu, J.; Chen, S.; Yang, K.; Wang, W.; Ning, G.; Tang, X.; Li, L.; Ye, Z.; et al. Molecular Classification in Fertility-Sparing Treatment of Early-Stage Endometrial Cancer: A Potential Tool for Optimizing Patient Selection. Gynecol. Oncol. 2024, 191, 240–248. https://doi.org/10.1016/j.ygyno.2024.10.012

36. Bogani, G.; De Vitis, L.; Mariani, A.; Multinu, F.; Vizzielli, G.; Ghezzi, F.; Borso, C.; Galli, L.; Caia, C.; Ferrari, F.; et al. Fertility-Sparing Approach in Endometrioid Grade II Endometrial Cancer: The Role of Molecular Classification. Int. J. Gynecol. Cancer 2025, 35, 102035. https://doi.org/10.1016/j.ijgc.2025.102035

37. De Vitis, L.A.; Schivardi, G.; Delfrati, S.; Biffi, B.; Viscardi, A.; Rosanu, M.; Ribero, L.; Caruso, G.; Rappa, A.; Marinucci, L.; et al. The Prognostic Impact of Molecular Classification in Endometrial Cancer That Undergoes Fertility-Sparing Treatment. Int. J. Gynecol. Cancer 2025, 35, 100024. https://doi.org/10.1016/j.ijgc.2024.100024

38. Dzyubak, O.; Glass, K.; Watts, M.; Tarafdar, O.; Simpson, A.N.; Maganti, M.; Deljoomanesh, S.; Laframboise, S.; Bernardini, M.Q.; Pakbaz, S.; et al. Oncologic and Fertility Outcomes of Progestin Therapy for Atypical Hyperplasia and Grade 1 Cancer of the Endometrium: Results of a Specialized Oncofertility Program. Gynecol. Oncol. 2025, 199, 72–78. https://doi.org/10.1016/j.ygyno.2025.06.015

39. Hsu, Y.T.; Hsu, H.C.; Lee, C.Y.; Hung, W.T.; Chen, C.H. Long-Term Outcomes of Fertility-Sparing Treatment in Endometrial Carcinoma and Endometrial Intraepithelial Neoplasia: Recurrence Risk Factors over a 9-Year Follow-Up. Acta Obstet. Gynecol. Scand. 2025, 104, 1994–2005. https://doi.org/10.1111/aogs.70028

40. Wang, J.; Jiang, G.; Yan, S.; Tian, Y.; Jin, Y.; Fu, H.; Si, L.; Cai, M.; Liu, X.; Guo, R. Molecular Classification and Fertility-Sparing Outcomes in Endometrial Cancer and Atypical Endometrial Hyperplasia. Biomol. Biomed. 2025, 26, 471–482. https://doi.org/10.17305/bb.2025.12445

41. Wang, Y.; Bo, L.; Fan, X.; Kang, N.; Zhang, X.; Tian, L.; Zhou, R.; Wang, J. Molecular Classification Guides Fertility-Sparing Treatment for Endometrial Cancer and Atypical Hyperplasia Patients. Curr. Oncol. 2025, 32, 317. https://doi.org/10.3390/curroncol32060317

50. Reid, H.W.; Young, A.N.; Yin, S.H.; Covelli Velez, I.; Abel, M.K.; Kolin, D.L.; Ginsburg, E.S.; Yurgelun, M.B.; Feltmate, C.; St Laurent, J.D. Clinical and Fertility Outcomes in Lynch Syndrome Patients with Endometrial Carcinoma or Endometrial Intraepthelial Neoplasia Treated with Fertility Sparing Management. Gynecol. Oncol. Rep. 2025, 59, 101768. https://doi.org/10.1016/j.gore.2025.101768

51. Chaudhari, S.R.; Lai, T.S.; Zakhour, M.; Myung Shin, S.; Baltayan, A.; Tan, H.; Cohen, J.G. Comparison of Mirena and Liletta Levonorgestrel Intrauterine Devices for the Treatment of Endometrial Intraepithelial Neoplasia and Grade 1 Endometrioid Endometrial Cancer. Gynecol. Oncol. Rep. 2023, 49, 101257. https://doi.org/10.1016/j.gore.2023.101257

52. Jang, E.B.; Lee, A.J.; So, K.A.; Lee, S.J.; Lee, J.Y.; Kim, T.J.; Park, E.; Kang, S.B.; Shim, S.H. Risk Factors for the Recurrence in Patients with Early Endometrioid Endometrial Cancer Achieving Complete Remission for Fertility-Sparing Hormonal Treatment. Gynecol. Oncol. 2024, 191, 19–24. https://doi.org/10.1016/j.ygyno.2024.09.015

53. Li, L.; Xiao, Z.; Wang, Y.; He, Y.; Wang, Z.; Shen, D.; Wang, J. Analysis of Immunohistochemical Characteristics and Recurrence after Complete Remission with Fertility Preservation Treatment in Patients with Endometrial Carcinoma and Endometrial Atypical Hyperplasia. Arch. Gynecol. Obstet. 2023, 307, 2025–2031. https://doi.org/10.1007/s00404-022-06398-3

54. Zhang, X.; Zhao, X.; Wang, C.; Lu, S.; Wang, Y.; He, Y.; Wang, J.; Shen, D. Use of Clinicopathological Factors to Predict Prognosis of Fertility-Sparing Treatment for Endometrial Endometrioid Carcinoma and Atypical Hyperplasia. Oncol. Lett. 2023, 25, 52. https://doi.org/10.3892/ol.2022.13638

55. Bowen, M.B.; Melendez, B.; Zhang, Q.; Yang, R.K.; Fellman, B.M.; Lawson, B.C.; Adjei, N.N.; Celestino, J.; Wani, K.M.; Singh, B.; et al. Long-Term Follow-Up of Levonorgestrel Intrauterine Device for Atypical Hyperplasia and Early Endometrial Cancer Reveals Relapse Characterized by Immune Exhaustion. Clin. Cancer Res. 2024, 30, 5073–5082. https://doi.org/10.1158/1078-0432.CCR-24-0362

56. Zhai, Z.; Wang, Y.; Li, H.; Kang, N.; Liu, Y.; Wang, J. Analysis of Fertility-Preserving Treatment Outcomes in Patients with POLE-Mutated Endometrioid Carcinoma. J. Gynecol. Oncol. 2025, 36, e101. https://doi.org/10.3802/jgo.2025.36.e101

57. Yang, X.; Xue, Y.; Shao, W.; Shan, W.; Xu, Z.; Wang, Y.; Chen, X. Fertility-Sparing Treatment Outcomes Using Immune Checkpoint Inhibitors in Endometrial Cancer Patients with Lynch Syndrome. J. Gynecol. Oncol. 2025, 36, e59. https://doi.org/10.3802/jgo.2025.36.e59

58. Puechl, A.M.; Spinosa, D.; Berchuck, A.; Secord, A.A.; Drury, K.E.; Broadwater, G.; Wong, J.; Whitaker, R.; Devos, N.; Corcoran, D.L.; et al. Molecular Classification to Prognosticate Response in Medically Managed Endometrial Cancers and Endometrial Intraepithelial Neoplasia. Cancers 2021, 13, 2847. https://doi.org/10.3390/cancers13112847

59. Catena, U.; Della Corte, L.; Raffone, A.; Travaglino, A.; Lucci Cordisco, E.; Teodorico, E.; Masciullo, V.; Bifulco, G.; Di Spiezio Sardo, A.; Scambia, G.; et al. Fertility-Sparing Treatment for Endometrial Cancer and Atypical Endometrial Hyperplasia in Patients with Lynch Syndrome: Molecular Diagnosis after Immunohistochemistry of MMR Proteins. Front. Med. 2022, 9, 948509. https://doi.org/10.3389/fmed.2022.948509

60. van Gent, M.D.; Nicolae-Cristea, A.R.; de Kroon, C.D.; Osse, E.M.; Kagie, M.J.; Trimbos, J.B.; Hazelbag, H.M.; Smit, V.T.; Bosse, T. Exploring Morphologic and Molecular Aspects of Endometrial Cancer under Progesterone Treatment in the Context of Fertility Preservation. Int. J. Gynecol. Cancer 2016, 26, 483–490. https://doi.org/10.1097/IGC.0000000000000629

61. Wang, Y.Q.; Kang, N.; Li, L.W.; Wang, Z.Q.; Zhou, R.; Shen, D.H.; Wang, J.L. Significance of Molecular Classification in Fertility-Sparing Treatment of Endometrial Carcinoma and Atypical Endometrial Hyperplasia. Zhonghua Fu Chan Ke Za Zhi 2022, 57, 692–700. (In Chinese). https://doi.org/10.3760/cma.j.cn112141-20220628-00419

62. Shao, W.Y.; Dong, Y.T.; Lyu, Q.Y.; Liao, J.B.; Xue, Y.; Chen, X.J. Fertility-Preserving Treatment Outcomes in Endometrial Cancer and Atypical Hyperplasia Patients with Different Molecular Profiles. Zhonghua Fu Chan Ke Za Zhi 2023, 58, 742–754. (In Chinese). https://doi.org/10.3760/cma.j.cn112141-20230719-00011

63. Wang, Y.; Kang, N.; Li, L.; Wang, Z.; Zhou, R.; Shen, D.; Wang, J. Characteristics of Molecular Classification in 52 Endometrial Cancer and Atypical Hyperplasia Patients Receiving Fertility-Sparing Treatment. Gynecol. Obstet. Clin. Med. 2023, 3, 38–43. https://doi.org/10.1016/j.gocm.2023.01.006

64. Zhang, X.; Chen, D.; Zhao, X.; Wang, C.; He, Y.; Chen, Y.; Wang, J.; Shen, D. Application of Molecular Classification to Guiding Fertility-Sparing Therapy for Patients with Endometrial Cancer or Endometrial Intraepithelial Neoplasia. Pathol. Res. Pract. 2023, 241, 154278. https://doi.org/10.1016/j.prp.2022.154278

65. Ran, X.; Hu, T.; Li, Z. Molecular Classification in Patients with Endometrial Cancer after Fertility-Preserving Treatment: Application of ProMisE Classifier and Combination of Prognostic Evidence. Front. Oncol. 2022, 12, 810631. https://doi.org/10.3389/fonc.2022.810631

66. Xue, Y.; Dong, Y.; Lou, Y.; Lv, Q.; Shan, W.; Wang, C.; Chen, X. PTEN Mutation Predicts Unfavorable Fertility Preserving Treatment Outcome in the Young Patients with Endometrioid Endometrial Cancer and Atypical Hyperplasia. J. Gynecol. Oncol. 2023, 34, e53. https://doi.org/10.3802/jgo.2023.34.e53
