# Supplementary material for: Fertility-Sparing Treatment of Endometrial Cancer and Atypical Endometrial Hyperplasia by Molecular Classification: A Systematic Review and Meta-Analysis Accounting for Outcome-Assessment Timing and Follow-Up Duration
Source: Cancers (Basel). 2026 Sep 14;18(18):2972. doi: 10.3390/cancers18182972 (PMC13605972; doi:10.3390/cancers18182972)
Supplement: Supplementary file 1 [file cancers-18-02972-s001.zip › Supplementary_Protocol_v1.1.pdf]

Pre-specified Analysis Plan (A Priori Protocol)

Fertility-Sparing Treatment of Endometrial Cancer and Atypical Endometrial Hyperplasia by Molecular Classification: A Systematic Review and Meta-Analysis Accounting for Outcome-Assessment Timing and Follow-Up Duration

Supplementary Material · Version 1.1 (revision) · Version 1.0 dated 02-Jul-2026; version 1.1 dated 20-Aug-2026 (see Section 1.4 and the version history / analysis-status table appended at the end)

Purpose and status of this document. This document records the analysis plan that was specified before the quantitative synthesis of the accompanying systematic review and meta-analysis. It is provided as supplementary material to promote transparency and to allow readers and reviewers to distinguish pre-specified analyses from any post hoc decisions. It is prepared in the format of the PRISMA-P (Preferred Reporting Items for Systematic review and Meta-Analysis Protocols) 2015 statement. This review was NOT prospectively registered in PROSPERO or another registry, and this document is not a time-stamped or third-party-verified registration; it does not claim priority of registration. All core analytic decisions described here were fixed before the pooled analyses were run, and any departures during conduct are reported in the manuscript.

1. Administrative Information

1.1 Title. Fertility-Sparing Treatment of Endometrial Cancer and Atypical Endometrial Hyperplasia by Molecular Classification: A Systematic Review and Meta-Analysis Accounting for Outcome-Assessment Timing and Follow-Up Duration. (Title of the revised manuscript; version 1.0 of this protocol was written under the original title "Fertility-Sparing Treatment in Endometrial Cancer and the Decisive Role of Measurement Timing: A Systematic Review and Timing-Aware Meta-Analysis of Oncologic Outcomes by Molecular Classification", which was changed during peer review at the reviewers' request.)

1.2 Registration. The review was not registered. This document records the pre-specified analysis plan and is submitted as supplementary material for transparency, in lieu of a registry entry.

1.3 Authors. Abeer Alatawi[1], Saleem Almaser[2], Malak Alanazi[2], Amirah Alatawi[3], Seongmin Kim[1] (corresponding author). [1] Department of Obstetrics and Gynecology, Korea University Medicine, Seoul, Republic of Korea. [2] Department of Obstetrics and Gynecology, Maternity and Children Hospital (MCH), Tabuk, Saudi Arabia. [3] Department of Internal Medicine, King Fahad Specialist Hospital, Tabuk, Saudi Arabia.

1.4 Amendments. This document reflects the analysis plan fixed prior to quantitative synthesis. No substantive amendments were made to the core analytic decisions—timepoint-separated pooling of complete response, meta-regression of recurrence on follow-up length, a priori consolidation of overlapping cohorts, proportion-based (rather than odds-ratio-based) synthesis, and the descriptive (non-pooled) treatment of p53-abnormal complete response. Any deviation arising during conduct is described explicitly in the manuscript.

1.5 Support. The review received no external funding. The authors declare no competing interests.

2. Introduction

2.1 Rationale. Fertility-sparing treatment (FST) is an established option for selected reproductive-age women with early-stage endometrial carcinoma (EC), and molecular classification (POLE-mutated, mismatch-repair-deficient [MMRd], no-specific-molecular-profile [NSMP], p53-abnormal [p53abn]) has been proposed to refine patient selection. However, FST cohorts are single-arm, so the quantity of interest is the subtype-specific proportion achieving complete response (CR) or recurrence; and the principal outcomes are time-dependent, because CR accrues over time and recurrence depends on follow-up duration. Pooling these outcomes without

accounting for measurement timing can confound response kinetics and surveillance length with tumour biology. A timing-aware, proportion-based synthesis was therefore planned.

2.2 Objectives. Using a population of reproductive-age women with EC (with or without atypical endometrial hyperplasia [AEH]) undergoing FST, stratified by molecular subtype, the review aimed to (1) estimate subtype-specific CR and post-CR recurrence using proportion-based methods appropriate to single-arm data; (2) determine which subtype differences persist after accounting for the timing of outcome measurement; and (3) define the timing-independent findings that should inform molecular-integrated FST counselling.

3. Methods

3.1 Eligibility criteria. Population: women with histologically confirmed EC, with or without concurrent AEH, seeking fertility preservation. Intervention: FST comprising progestins (megestrol/medroxyprogesterone acetate) or a levonorgestrel-releasing intrauterine device, with or without hysteroscopic resection. Comparison/stratification: outcomes reported by validated molecular classification (TCGA, ProMisE, or immunohistochemistry/sequencing surrogates). Outcomes: CR (primary); recurrence after CR and disease progression (secondary). Study designs: cohort studies and case series with extractable, subtype-resolved outcomes. Exclusions: case reports; series with fewer than 10 participants; reviews; protocols without outcome data; conference abstracts without full text; non-FST modalities; and studies without extractable, subtype-resolved outcomes.

3.2 Information sources. PubMed (MEDLINE) and Embase were searched from January 2015 to August 2025. Reference lists of eligible studies and relevant reviews were hand-searched. (No date or language restriction was imposed beyond the search window.)

3.3 Search strategy. The search combined four concept blocks—endometrial carcinoma; fertility-sparing/conservative treatment; hormonal therapy; and molecular/genomic classification—using controlled vocabulary and free-text terms joined with Boolean operators. Full database-specific search strings were retained by the review team and are available on request; a copy may be provided as an additional supplementary file if required by the editors.

3.4 Study records — data management. Records were de-duplicated and managed in a shared reference/spreadsheet workflow.

Study records — selection process. Titles, abstracts, and full texts were screened in duplicate by two reviewers working independently, with disagreements resolved by consensus.

Study records — data collection process. Data were extracted directly from primary sources by two reviewers; subtype-specific numerators and denominators were independently recalculated for consistency.

3.5 Data items. For each cohort: study design, country, enrollment period, sample size and AEH/EC composition, FIGO stage, treatment modality (including hysteroscopic resection and any adjuncts such as gonadotropin-releasing hormone agonist, letrozole, metformin, or immune checkpoint inhibition), molecular classifier, and median follow-up. Outcome data: subtype-specific numerators/denominators for CR at both 6 months and best/overall response, for recurrence among CR-achievers, and for progression where reported.

3.6 Outcomes and prioritization. The primary outcome was complete response, pre-specified to be pooled separately at two timepoints—(i) 6 months and (ii) best/overall response—because CR accrues over time. Secondary outcomes were recurrence among CR-achievers (analyzed as a function of median follow-up) and disease progression. Reproductive outcomes (pregnancy, live birth) were pre-specified for narrative summary rather than pooling, owing to heterogeneous denominators and definitions.

3.7 Resolution of overlapping cohorts (pre-specified). Because multiple reports may describe the same underlying cohort, overlap clusters were identified a priori from shared institution, senior author, and enrollment window. To prevent double-counting, a single cohort was retained per cluster, prioritizing the report with the most complete subtype distribution, dual CR timepoints, and multivariable or fertility data. Cohorts reporting only mismatch-repair status (MMR-binary) or restricted to a single tumour grade were reserved for sensitivity analyses; a non-FST cohort was excluded from CR pooling.

3.8 Risk of bias in individual studies. Methodological quality of each non-randomized study was assessed with the ROBINS-I tool across its seven domains, with an overall judgment per study.

3.9 Data synthesis — choice of effect measure. Because FST cohorts are single-arm, the clinically meaningful quantity is the subtype-specific proportion; proportions were therefore synthesized directly rather than comparative effect measures, which would require an external comparator that these cohorts do not provide.

Data synthesis — pooling model. Proportions were pooled with a binomial–normal random-intercept generalized linear mixed model (logit-GLMM) fitted by 40-point Gauss–Hermite quadrature, using the exact binomial likelihood and accommodating zero-event and all-event cells without continuity corrections. Heterogeneity was summarized by the between-study standard deviation on the logit scale (τ) and by 95% prediction intervals (considered more informative than I^2 for single-proportion meta-analysis).

Data synthesis — timing analyses (moderators). CR was pooled separately at 6 months and best response. The within-cohort timepoint effect was estimated in cohorts reporting both timepoints using a binomial model with cohort fixed effects and a timepoint indicator (isolating the within-patient change). Recurrence among CR-achievers was meta-regressed on median follow-up (logit-linear, random intercept).

Data synthesis — pre-specified secondary analyses. p53abn CR was summarized descriptively rather than pooled, owing to small, unstable denominators. Occult high-risk prevalence (p53abn and MMRd among apparent FST candidates) was pooled as a proportion, and the number-needed-to-test was computed as the inverse of the combined prevalence.

3.10 Sensitivity analyses (pre-specified). (i) An MMR-binary expansion adding cohorts reporting only MMR status; (ii) a cohort-cluster swap exchanging the retained Zhengzhou cohort for its alternative; and (iii) leave-one-out across all pools and the meta-regression.

3.11 Meta-bias(es). Given the small number of studies per subtype, formal assessment of small-study and publication bias (e.g., funnel-plot asymmetry tests) was not planned; the potential for reporting bias is addressed narratively in the manuscript.

3.12 Confidence in cumulative evidence. Certainty of evidence was rated per outcome using the GRADE framework, beginning at low for observational data and modified for imprecision, inconsistency, indirectness, and upgrading factors (large effect, dose–response, within-cohort design).

Software. Analyses were performed in Python with a custom Gauss–Hermite GLMM implementation and statsmodels; two-sided $p < 0.05$ was considered significant.

Version history

Version 1.0 — 2 July 2026 (before quantitative synthesis; submitted with the original manuscript on 3 July 2026). This version documents the analysis plan fixed before the pooled analyses were run. It was not registered and is not a time-stamped third-party record.

Version 1.1 — 20 August 2026 (revision after peer review). No pre-specified analysis was removed. The following were added in response to reviewers and are labelled post hoc in the manuscript: (a) restriction of the primary syntheses to the four-class-classified cohorts of standard fertility-sparing populations, with the grade-2-only cohort and MMR-binary cohorts confined to sensitivity analyses (this corrects an inconsistency in the original figures); (b) a conditional late-response analysis (late CR among early non-responders) with formal subtype-by-timepoint tests, replacing the cohort-fixed-effects two-timepoint model; (c) recurrence models with follow-up adjustment and a subtype-by-follow-up interaction; (d) EC-only versus mixed AEH/EC cohort comparisons; (e) a descriptive synthesis of disease progression; (f) sensitivity analyses excluding the cohort using immune checkpoint inhibitors and the two cohorts whose early assessment was not at 6 months (32 weeks; 8 months); (g) risk of bias re-assessed with QUIPS instead of ROBINS-I; (h) GRADE re-applied under the prognostic-factor framework with standard categories; (i) adaptive (instead of non-adaptive) Gauss–Hermite quadrature with explicit convergence criteria and validation against metafor/lme4; (j) public deposition of data and code (https://github.com/naiad515/fst-endometrial-molecular-meta; Zenodo DOI 10.5281/zenodo.22006102); (k) re-extraction of every value from the full text of all 15 primary reports (publisher PDFs obtained for the eight subscription-journal reports), with the corrections listed in the response to reviewers; (l) because the database strings as executed in August 2025 were not archived, the search strategy was reconstructed from the concept blocks in Section 3.3, re-executed in PubMed and Embase on 19 August 2026 with the original date limits, verified against the eligible reports and reported in full in Supplementary Table S1a (Section 3.3's statement that the strings were retained by the review team could not be honoured). Model predictions from the follow-up meta-regression are restricted to the observed follow-up range. (m) The manuscript title was changed at the reviewers' request; the title in this document has been updated accordingly (Section 1.1 records the original title). (n) In response to an additional report received after resubmission, a post hoc comparison of the primary one-stage logit-GLMM with two-stage DerSimonian–Laird pooling was added (Supplementary Table S6e; code dl_comparison.py), and the reasons for the absence of PROSPERO registration are stated in Section 2.1 of the manuscript.

Analysis-status table (pre-specified in version 1.0 versus added post hoc in version 1.1)

Analysis	Status	Outcome
Proportion-based synthesis (logit-GLMM) rather than odds ratios	Pre-specified (v1.0)	Performed
CR pooled separately at 6 months and at best/overall response	Pre-specified (v1.0)	Performed; early timepoint defined as 6 months (32 weeks in one cohort; the second 3-monthly evaluation, labelled 8 months, in one cohort) and analysed also within paired cohorts (six cohorts)
Within-cohort timepoint effect (cohort fixed effects + timepoint indicator)	Pre-specified (v1.0)	Replaced by the conditional late-response analysis (post hoc) because the two timepoints are correlated; the aggregate two-timepoint model is reported for completeness only
Meta-regression of recurrence on median follow-up	Pre-specified (v1.0)	Performed; labelled exploratory; predictions restricted to the observed range; leave-one-out reported
Subtype-by-follow-up interaction model for recurrence	Post hoc (v1.1)	Performed
Formal subtype contrasts (mixed models with subtype effects and cohort random intercept)	Post hoc (v1.1)	Performed

Consolidation of overlapping cohorts (one report per cluster); cluster-swap sensitivity	Pre-specified (v1.0)	Performed
Descriptive (non-pooled) treatment of p53abn CR	Pre-specified (v1.0)	Performed
Occult high-risk prevalence and number needed to test	Pre-specified (v1.0)	Performed
MMR-binary expansion; leave-one-out	Pre-specified (v1.0)	Performed; recurrence pooled only from the three MMR-binary cohorts reporting relapse by MMR status (one cohort reports recurrence unstratified and one grade-2 cohort does not report relapse after CR by class)
Disease progression as a secondary outcome	Pre-specified (v1.0)	Quantitative synthesis not performed in the original submission (definitional heterogeneity); descriptive synthesis added post hoc (v1.1)
Reproductive outcomes — narrative	Pre-specified (v1.0)	Performed
EC-only versus mixed AEH/EC cohort comparisons; grade-2 cohort inclusion; ICI-cohort exclusion; exclusion of the two cohorts with a non-6-month early assessment	Post hoc (v1.1)	Performed
Risk of bias: ROBINS-I	Pre-specified (v1.0)	Replaced by QUIPS (post hoc, v1.1)
GRADE certainty	Pre-specified (v1.0)	Re-applied under the prognostic-factor framework with standard categories (v1.1)
Public data and code deposition	Post hoc (v1.1)	Done (https://github.com/naiad515/fst-endometrial-molecular-meta; Zenodo DOI 10.5281/zenodo.22006102)
